# Missense variants in *ANO4* cause sporadic encephalopathic or familial epilepsy with evidence for a dominant-negative effect

**DOI:** 10.1016/j.ajhg.2024.04.014

**Published:** 2024-05-13

**Authors:** Fang Yang, Anais Begemann, Nadine Reichhart, Akvile Haeckel, Katharina Steindl, Eyk Schellenberger, Ronja Fini Sturm, Magalie Barth, Sissy Bassani, Paranchai Boonsawat, Thomas Courtin, Bruno Delobel, EuroEPINOMICS-RES Dravet working group, Boudewijn Gunning, Katia Hardies, Mélanie Jennesson, Louis Legoff, Tarja Linnankivi, Clément Prouteau, Noor Smal, Marta Spodenkiewicz, Sandra P. Toelle, Koen Van Gassen, Wim Van Paesschen, Nienke Verbeek, Alban Ziegler, Markus Zweier, Anselm H.C. Horn, Heinrich Sticht, Holger Lerche, Sarah Weckhuysen, Olaf Strauß, Anita Rauch

**Affiliations:** 1 Experimental Ophthalmology, Department of Ophthalmology, Charité – Universitätsmedizin Berlin, a Corporate Member of Freie Universität, Humboldt-University, the Berlin Institute of Health, Berlin, Germany; 2 Institute of Medical Genetics, University of Zurich, Schlieren-Zurich, Switzerland; 3 Institute for Radiology and Children’s Radiology, Charité–Universitätsmedizin Berlin, a Corporate Member of Freie Universität, Humboldt-University, the Berlin Institute of Health, Berlin, Germany; 4 University Hospital of Angers, Department of Genetics, Angers, France; 5 Sorbonne Université, INSERM, CNRS, Institut du Cerveau - Paris Brain Institute - ICM, 75013 Paris, France; 6 Hôpital Pitié-Salpêtrière, DMU BioGe'M, AP-HP, 75013 Paris, France; 7 Service de Cytogénétique, GH de l'Institut Catholique de Lille, Hopital Saint Vincent de Paul, Lille, France; 8 Stichting Epilepsie Instellingen Nederland, Zwolle, the Netherlands; 9 Applied & Translational Neurogenomics Group, VIB Center for Molecular Neurology, VIB, University of Antwerp, 2610 Antwerp, Belgium; 10 Department of Pediatrics, CHU, Reims, France; 11 Epilepsia Helsinki, University of Helsinki and Helsinki University Hospital, 00029 HUS Helsinki, Finland; 12 Department of Pediatric Neurology and Pediatric Research Center, New Children’s Hospital, Helsinki University Hospital and University of Helsinki, 00029 HUS Helsinki, Finland; 13 Department of Genetics, La Réunion University Hospital, Saint-Pierre, France; 14 Department of Pediatric Neurology, Children’s University Hospital Zurich, Zurich, Switzerland; 15 University Medical Center Utrecht, Department of Genetics, Utrecht, the Netherlands; 16 Laboratory for Epilepsy Research, KU Leuven, and Neurology Department, University Hospitals Leuven, 3000 Leuven, Belgium; 17 Division of Bioinformatics, Institute of Biochemistry, Friedrich-Alexander-Universität Erlangen-Nürnberg, Erlangen, Germany; 18 Department of Neurology and Epileptology, Hertie-Institute for Clinical Brain Research, University of Tübingen, Tübingen, Germany; 19 Department of Neurology, Antwerp University Hospital, Antwerp, Belgium; 20 Translational Neurosciences, Faculty of Medicine and Health Science, University of Antwerp, 2610 Antwerp, Belgium; 21 Children’s University Hospital Zurich, Zurich, Switzerland

**Keywords:** *ANO4*, *TMEM16D*, anoctamin, developmental and epileptic encephalopathy, temporal lobe epilepsy, GEFS+, Ca^2+^-dependent ion channel, phospholipid scramblase

## Abstract

Anoctamins are a family of Ca^2+^-activated proteins that may act as ion channels and/or phospholipid scramblases with limited understanding of function and disease association. Here, we identified five *de novo* and two inherited missense variants in *ANO4* (alias *TMEM16D*) as a cause of fever-sensitive developmental and epileptic or epileptic encephalopathy (DEE/EE) and generalized epilepsy with febrile seizures plus (GEFS+) or temporal lobe epilepsy. *In silico* modeling of the ANO4 structure predicted that all identified variants lead to destabilization of the ANO4 structure. Four variants are localized close to the Ca^2+^ binding sites of ANO4, suggesting impaired protein function. Variant mapping to the protein topology suggests a preliminary genotype-phenotype correlation. Moreover, the observation of a heterozygous *ANO4* deletion in a healthy individual suggests a dysfunctional protein as disease mechanism rather than haploinsufficiency. To test this hypothesis, we examined mutant ANO4 functional properties in a heterologous expression system by patch-clamp recordings, immunocytochemistry, and surface expression of annexin A5 as a measure of phosphatidylserine scramblase activity. All *ANO4* variants showed severe loss of ion channel function and DEE/EE associated variants presented mild loss of surface expression due to impaired plasma membrane trafficking. Increased levels of Ca^2+^-independent annexin A5 at the cell surface suggested an increased apoptosis rate in DEE-mutant expressing cells, but no changes in Ca^2+^-dependent scramblase activity were observed. Co-transfection with ANO4 wild-type suggested a dominant-negative effect. In summary, we expand the genetic base for both encephalopathic sporadic and inherited fever-sensitive epilepsies and link germline variants in *ANO4* to a hereditary disease.

## Teaser

This paper establishes missense variants in the anoctamin family member *ANO4* (or *TMEM16D*) as a cause of distinct epileptic disorders. Resulting phenotypes depend on the location in the protein and the functional impact of the substitution such as loss of cation channel function and increased apoptosis signaling.

## Introduction

During the last decade, the important contribution of Mendelian disorders to the etiology of epilepsies became increasingly evident. According to a current meta-analysis, overall diagnostic yields are 24% for exome sequencing and 48% for genome sequencing^1^ but are highly variable depending on cohort selection and date of the study. Moreover, since exome sequencing has improved over time, the current diagnostic yield of exome sequencing is expected to be closer to genome sequencing as pointed out by Grether et al.^2^ The diagnostic yield of genetic testing is highest in early-onset developmental and epileptic or epileptic encephalopathy (DEE/EE), which are severe disorders combining early-onset epilepsy and neurodevelopmental delay, but still about half of the cases remain without diagnosis.^3^^,^^4^ Undiagnosed cases may be explained by variants that are difficult to detect, variants of uncertain significance, or novel disease-associated genes. Here, we present evidence that variants in *ANO4* (anoctamin 4 or *TMEM16D* [MIM: 610111]) cause sporadic encephalopathic and inherited epilepsy.

Anoctamins (ANOs) are highly conserved calcium-activated proteins. The ANO family encompasses ten members (ANO1 to ANO10, alias TMEM16A-K) that assemble as dimers^5^ and function as Ca^2+^-dependent ion channels and Ca^2+^-dependent phospholipid scramblases.^5^^,^^6^^,^^7^^,^^8^^,^^9^^,^^10^^,^^11^ Phospholipid scrambling refers to the translocation of phospholipids between the two leaflets of the lipid bilayer membrane, leading to loss of lipid asymmetry and to the externalization of phosphatidylserine (PS). Exposure of PS is essential for platelet activation and promotion of blood coagulation, suppression of inflammatory responses, and functions as a marker of apoptotic cells for recognition and clearance.^12^ ANO1 (MIM: 610108), ANO2 (MIM: 610109), ANO4, ANO6 (MIM: 608663), and ANO7 (MIM: 605096) are predominantly located in the plasma membrane, while ANO8 (MIM: 610216), ANO9 (MIM: 619963), and ANO10 (MIM: 613726) are mostly expressed in the cytosol.^13^^,^^14^ ANO1 and ANO2 have been shown to function as Ca^2+^-dependent Cl^−^ channels.^15^^,^^16^^,^^17^^,^^18^^,^^19^ ANO3 (MIM: 610110), ANO4, ANO5 (MIM: 608662), ANO6, ANO7, and ANO9 were reported as phospholipid scramblases and/or ion channels and possibly regulate the activity of other endogenous ion channels due to their phospholipid scramblase activity.^8^^,^^9^^,^^11^^,^^20^^,^^21^^,^^22^^,^^23^^,^^24^

ANO proteins show multiple tissue-specific isoforms, which display distinct gating properties.^9^^,^^23^^,^^25^^,^^26^^,^^27^^,^^28^^,^^29^^,^^30^ ANO proteins are involved in the control of neuronal cell excitability, olfaction, nociception, epithelial fluid secretion, contraction of smooth muscle, repair of the skeletal muscle membrane, and tumorigenesis. The broad spectrum of tissue expression explains the wide range of disorders associated with their respective disrupted function. Several ANO family members have been associated with a variety of human diseases or increased risks for diseases such as intestinal dysmotility (MIM: 620045)^31^^,^^32^ and moyamoya disease (MIM: 620687)^33^ with *ANO1*, dystonia (MIM: 615034)^34^^,^^35^ with *ANO3*, gnathodiaphyseal dysplasia (MIM: 166260)^36^^,^^37^^,^^38^^,^^39^ and muscular dystrophies (MIM: 613319, 611307)^40^^,^^41^^,^^42^^,^^43^^,^^44^ with *ANO5*, disturbed blood coagulation (Scott syndrome, MIM 262890)^24^^,^^45^ with *ANO6*, spinocerebellar ataxia (MIM: 613728)^46^^,^^47^^,^^48^ with *ANO10*, and pancreatic and colorectal cancer^49^^,^^50^ with *ANO9*. *ANO4* has not been linked to a Mendelian phenotype so far, but genome-wide association studies showed an association between single-nucleotide polymorphisms near *ANO4* and various neurological diseases, such as schizophrenia, Alzheimer disease, and anxiety disorder.^51^^,^^52^^,^^53^^,^^54^

Compared to well-studied members of the ANO family (ANO1, ANO2, and ANO6), the physiological functions and pathophysiology of ANO4 remain poorly investigated and have long been debated. ANO4 is mainly expressed in the central nervous system, particularly in inhibitory neurons and oligodendrocytes, and in endocrine and reproductive tissues.^55^ During human embryonic development, *ANO4* mRNA is expressed in the human neocortex and cerebellar cortex with its peak in the mid-fetal development stages, then levels decrease during postnatal development and remain relatively stable in adulthood (Figure S1).^56^ In contrast, hippocampus, amygdala, mediodorsal nucleus of the thalamus, and striatum display relatively low *ANO4* mRNA levels during prenatal development, with maximal detection during the postnatal periods.

Brown and colleagues indicated that ANO4 might participate in the regulation of aldosterone secretion and refuted its putative role as a Ca^2+^-dependent chloride channel.^57^^,^^58^ Furthermore, the levels of ANO4 expression were increased in active myelin lesions in multiple sclerosis.^59^ A meta-analysis of genome-wide association studies in breast cancer identified variants around the ANO4 locus as risk factors.^60^ Recent work more precisely described the function of ANO4 as being a Ca^2+^-dependent cation channel involved in the regulation of Ca^2+^ signaling in both plasma membranes and membranes of organelles when heterologously expressed in human embryonal kidney (HEK293) cells or when endogenously expressed in retinal pigment epithelial cells.^61^ Potential scramblase activity of ANO4 remains uncertain.^21^^,^^61^ More recent investigations revealed that currents facilitated by the ANO4 channel are required to activate glucose-inhibited neurons situated in the ventromedial hypothalamic nucleus (VMH) of mice in reaction to diminished glucose levels. Knockout of *Ano4* within the VMH exhibited a dual outcome: a decrease in blood glucose levels and a compromised ability to mount counterregulatory responses to hypoglycemic episodes.^62^ A further mouse model carrying a homozygous intragenic deletion in *Ano4* presented with hyperactivity, reduced bone mineral content, decreased body weight, and altered cholesterol ratio compared to wild-type mice (Mouse Genome Database [MGD]; MGI: 2443344). Ano4 overexpression has been shown to attenuate calcium-mediated aldosterone secretion and reduce cell proliferation in a human adenocarcinoma cell line (H295R).^57^ Furthermore, ANO4 was found upregulated in human tumor samples compared to normal kidney tissue, suggesting a potential role as a prognostic biomarker in non-metastasized clear cell renal cell carcinoma.^31^

In this paper, we describe seven *ANO4* missense variants identified in either sporadic individuals with DEE or EE or families with genetic epilepsy with febrile seizures plus (GEFS+) or temporal lobe epilepsy (TLE). We evaluated functional effects of the variants by overexpressing wild-type or mutant *ANO4* constructs in HEK293 cells. We measured the plasma membrane and subcellular localization by immunostaining and ion conductance using whole-cell patch clamping, as well as scramblase activity by binding of fluorescently labeled annexin A5 to exposed PS. We further employed structural modeling to gain insights into genotype-phenotype correlation.

## Material and methods

### Study cohort and genetic analyses

Through the GeneMatcher platform,^63^ we recruited/identified five individuals (I1–I5) with epilepsy and intellectual disability (ID) harboring *de novo ANO4* missense variants identified by trio exome sequencing at the respective center as the only candidate causative variants. I1 was studied within the cohort published by Papuc et al.,^4^ remaining without definite diagnosis. I4 was exome sequenced as part of the efforts of the EuroEPINOMICS-RES consortium. All detected rare variants (minor allele frequency [MAF] <0.001) with fitting inheritance pattern of I1, I2, I4, and I5 are provided in Table S1 (data from I3 not available). Family 6 (F6) consists of a previously published large pedigree with 23 family members affected by GEFS+ (formerly described as familial TLE with febrile seizures) with autosomal-dominant inheritance pattern (Figure S2).^64^ By linkage analysis in this family, the disease gene locus was previously mapped to 12q22–q23.3, narrowing down the candidate region to a 10.35 cM (8.7 Mb) interval between D12S101 and D12S360 with the highest two-point LOD score of 6.94 and multipoint LOD score of 8.87 for marker D12S1706.^65^ Whole-genome sequencing in two affected family members revealed a single candidate missense variant in *ANO4* that segregated within the family. Only one other rare variant was present in the linked locus in *PAH*, which is not a candidate variant for epilepsy since it is associated with the well-established autosomal-recessive disease phenylketonuria (Table S1). Following this finding, I7 with a phenotype of TLE was identified through candidate gene sequencing in a cohort of individuals with febrile seizures and/or TLE. All rare variants with MAF <0.001 detected by an exome-based epilepsy panel performed in I7 are provided in the Table S1. Individual ID: 272667 was recruited through the database of genomic variation and phenotype in humans using ensembl resources (DECIPHER).^66^ The procedures followed were in accordance with the ethical standards of the respective responsible committees on human experimentation, and proper informed consent was obtained. *ANO4* variant nomenclature is given according to reference transcript GenBank: NM_001286615.1 (corresponding to ENST00000392977.3) and reference protein GenBank: NP_001273544.1 throughout the manuscript.

### Structural analysis of ANO4 variants

The membrane topology model of ANO4 (GRCh37 [GenBank: NM_001286615.1]) in Figure 2A was created based on sequence alignment to the experimentally observed topology of the ortholog in *N. haematococca*.^5^ The indicated dimerization domain was determined by alignment to the published dimerization domain in mouse Ano1.^67^ The figure was drawn with BioRender online software.

The structural analysis of ANO4 was based on a model available in the AlphaFold Protein Structure Database (Q32M45).^68^^,^^69^ The subunit orientation and location of ion binding sites was adopted from the experimental structure of murine Ano6 (Protein Data Bank [PDB]: 6QP6).^70^ VMD^71^ was used for structure analysis and visualization.

### Cell culture and transfection

All experiments were performed in HEK293 cells (CRL-1573; ATCC, Wesel, Germany) with low passage numbers. These cells are a standard model to test ion channel variants, and as shown in our previous work, they do not show endogenous ANO4-induced currents.^61^ HEK293 cells were grown in 25-cm^2^ tissue culture flasks in Dulbecco’s Modified Eagle’s Medium (Gibco, Karlsruhe, Germany) supplemented with 10% (v/v) fetal bovine serum (Biochrom, Berlin, Germany) and 1% (v/v) penicillin-streptomycin (Biochrom, Berlin, Germany). Cells were maintained at 37°C and 5% CO2 in an incubator with 95% relative humidity. For functional assays, HEK293 cells were seeded on 12-well plates with sterilized 15-mm glass cover slips previously coated with 0.01% (w/v) Poly-L-lysine (Sigma-Aldrich, Co, St. Louis, USA) and transfected with the *ANO4* plasmids the following day using Lipofectamine 2000 Transfection Reagent (Invitrogen, Darmstadt, Germany), according to the manufacturer’s protocol. For transfection, we mixed 0.2 μg plasmid DNA with 0.4 μL Lipofectamine. In the case of wild-type and mutant combined transfection, 0.2 μg contained 50% wild-type and 50% mutant Ano4 plasmid.

### Site-directed mutagenesis of *ANO4*

The full-length coding sequence of mouse *Ano4* was acquired with the previously described method.^61^ Sequence alignments of *ANO4* were performed using Clustal Omega.^72^ Locations of human disease-associated *ANO4* variants were identified as homologous nucleotide sites in mouse *Ano4* by sequence alignment between human *ANO4* and mouse *Ano4* (Figure S3). *ANO4* variants were inserted by site-directed mutagenesis into the full-length *Ano4* background*.* Site-directed mutagenesis was accomplished using the QuickChange site-directed mutagenesis kit (Agilent Technologies, Santa Clara, CA, USA), according to the manufacturer’s recommendation. The sequences of primer pairs are shown in Table 1. *ANO4* mutants Met563Lys, Asn558Lys, Ile562Phe, Asn603Asp, Asn129Lys, Val528Met, and Ile725Thr were generated. All final mutagenized *ANO4* constructs were fully sequenced before expression to confirm the accuracy of targeted mutagenesis and to exclude any unexpected sequence alterations.

**Table 1:** The missense variants were generated using wild-type *ANO4* as template and the following primers

Met563Lys	F: GCACATTCAGCAGCTTAATGATACAAAAGTTGATGCATACTG
	R: CAGTATGCATCAACTTTTGTATCATTAAGCTGCTGAATGTGC
Asn558Lys	F: GCAGCATAATGATACAAAACTTGATGCATACTGCAGTCC
	R: GGACTGCAGTATGCATCAAGTTTTGTATCATTATGCTGC
Ile562Phe	F: AGAGCACATTCAGCAGCATAAAGATACAAAAGTTGATGCATAC
	R: GTATGCATCAACTTTTGTATCTTTATGCTGCTGAATGTGCTCT
Asn603Asp	F: GTAAAAAGTGGAGCTGTTCAGATCGACAAACTGAAAAAGAAACATTT
	R: AAATGTTTCTTTTTCAGTTTGTCGATCTGAACAGCTCCACTTTTTAC
Asn129Lys	F: CTTTTCAGTCTGAGGCTTGGATTTTCTGTACACAAGAATG
	R: CATTCTTGTGTACAGAAAATCCAAGCCTCAGACTGAAAAG
Val528Met	F: GGATCGTCATTTACAGGATGGTGACTGTGAGCACT
	R: AGTGCTCACAGTCACCATCCTGTAAATGACGATCC
Ile725Thr	F: GAAAAGCTGCCACAAAGGTCGTTGTGAATCCGAAC
	R: GTTCGGATTCACAACGACCTTTGTGGCAGCTTTTC

### Patch-clamp recordings

Membrane currents of wild-type or mutant *ANO4* transfected cells were measured in the whole-cell configuration of the patch-clamp technique. Recordings were performed using single transfected cells that were identified by detection of co-transfected GFP fluorescence. Transfected HEK293 cells were kept in a serum-free medium for 24 h prior to patch-clamp analysis. Patch pipettes were pulled from borosilicate glass capillaries with a pipette resistance ranging from 3 to 5 MΩ using a DMZ Universal Puller (Zeitz, Augsburg, Germany). Whole-cell currents were recorded with an EPC 9 patch-clamp amplifier, and data acquisition and analysis were performed with TIDA hardware and software (HEKA Electronics, Lambrecht, Germany). Current signals were filtered at 2.9 kHz with a low-pass Bessel filter. The mean membrane capacitance was 21 pF (*n* = 77). Access resistance was compensated for a voltage error of 3 mV per 1nA. While recording, the 15 mm glass cover slips with transfected cells were superfused with an extracellular Ringer solution containing (in mM): NaCl 145, KH_2_PO_4_ 0.4, K_2_HPO_4_ 1.6, and Ca-gluconate 1.3. Pipettes were filled with a pipette solution containing 95 mM K-gluconate, 30 mM KCl, 4.8 mM Na_2_HPO_4_, 1.2 mM NaH_2_PO_4_, 0.3 mM Ca-gluconate, and 1 mM EGTA. The pH value of both solutions was adjusted to 7.2 by Tris base. Potentials were corrected for a liquid junction potential of +10.7 mV.

### Immunostaining and co-localization analysis

24 h after transfection, HEK293 cells were fixed with 4% (w/v) paraformaldehyde for 10 min and then washed three times with 1 × Tris-buffered saline (TBS), permeabilized with 0.5% (v/v) Triton X-100 for 10 min at room temperature. After three washes in TBS, cells were then incubated with a blocking solution (5% bovine serum albumin in TBS) for 45 min. Cells were co-labeled overnight at 4°C with primary antibody against ANO4 (rabbit polyclonal, 1:100; Abcam, ab170008) and primary antibody against pan-cadherin (mouse polyclonal, 1:200; Abcam, ab6528), which was used to stain the cell membrane, or primary antibody against early endosome antigen 1 (EEA1) (sheep polyclonal, 1:100; R&D, AF8047). After a 45 min incubation with fluorescence conjugated appropriate species-specific secondary antibodies, donkey anti-rabbit Alexa Fluor 546 (1:1000; Invitrogen by Thermo Fisher Scientific, A10040) and donkey anti-mouse Alexa Fluor 647 (1:1000; Invitrogen by Thermo Fisher Scientific, A-31571) or donkey anti-sheep Alexa Fluor 488 (1:1000; Invitrogen by Thermo Fisher Scientific, A-11015), DAPI (4′,6-diamidino-2-phenylindole) (Sigma-Aldrich, Co, St. Louis, USA), was used to stain nuclear DNA. Cells were mounted onto glass slides with a fluorescence mounting medium (Dako, Germany) and examined by confocal microscopy (Leica SPE, Germany). Confocal fluorescence microscopy images were quantitatively analyzed using an ImageJ software package (Rasband, W.S., ImageJ, US National Institutes of Health, Bethesda, Maryland, USA, 1997–2015). The quantitative co-localization analysis of ANO4 and pan-cadherin or EEA1 were performed by Pearson’s correlation coefficient (PCC) to evaluate the degree of co-localization. Values of PCC range from −1 to 1, where a negative value (−1) indicates no overlap, and 1 is a complete co-localization.

### Measurement of scramblase activity

To explore the influence of mutant ANO4 on scramblase activity, PS exposure was examined by Fluorescence-Activated Cell Sorting (FACS). Annexin A5 is a Ca^2+^-binding protein that can bind to PS, and therefore fluorescently labeled annexin A5 can be used to detect PS exposed to the outside of the cells. Scramblase activity was determined by measuring the fluorescently labeled annexin A5 binding to exposed PS. ANO4 mutant transfected HEK293 cell pellets (0.5–1 × 10^6^ cells) were collected and divided equally into two tubes, one of which was treated with 1 μM ionomycin for 10 min. After washing once with 500 μL PBS, the cells were resuspended in 500 μL annexin A5 binding buffer (1.3 mM CaCl_2_, 10 mM HEPES, 150 mM NaCl, 5 mM KCl, 1 mM MgCl_2_, pH 7.4), each sample was incubated with 0.25 μg of cys-anxA5-6S-IDCC in the dark for 10 min. Cys-anxA5-6S-IDCC comes from cys-annexin-A5 (NeXins Research, the Netherlands) labeled with maleimide-6S-IDCC (Mivenion GmbH, Berlin, Germany) at the singular cysteins as described elsewhere.^73^ Data acquisition and analysis of annexin A5 binding and GFP-expression were performed using a BD Accuri C6 flow cytometer with BD Accuri C6 software (BD Biosciences, Heidelberg, Germany), according to the manufacturer’s instructions.

### Statistical analysis

All experiments were repeated independently at least three times, and one representative result from each group was displayed. Mean values were given as mean ± SEM; n represents the number of experiments. Statistical differences between groups were tested using GraphPad Prism (GraphPad Software, Inc, USA) by multicomparisons with ANOVA and post hoc analysis (either Dunn’s or Tukey’s multiple comparisons post hoc test) with confidence intervals of 0.05. *p* values of less than 0.05 were considered statistically significant. *p* values are indicated in all figures according to convention: ^∗^*p* < 0.05; ^∗∗^*p* < 0.01; ^∗∗∗^*p* < 0.001; ^∗∗∗∗^*p* < 0.0001; and ns, not significant.

## Results

### Study cohort and genetic variants

Clinical details and nomenclature of detected variants are provided in Tables 2, 3, and 4 and the case reports in the supplemental note.

**Table 2:** Overview of observed *ANO4* variants in our study cohort

**Individual**	**I1**	**I2**	**I3**	**I4**	**I5**	**F6**	**I7**
*ANO4* variant on gDNA level on NC_000012.11 (hg19)	g.101480589T>A	g.101480575C>A	g.101480585A>T	g.101490382 A>G	g.101336244C>G	g.101480483G>A	g.101504206T>C
cDNA level on NM_001286615.1	c.1688T>A	c.1674C>A	c.1684A>T	c.1807A>G	c.387C>G	c.1582G>A	c.2174T>C
Protein level on NP_001273544.1	p.Met563Lys	p.Asn558Lys	p.Ile562Phe	p.Asn603Asp	p.Asn129Lys	p.Val528Met	p.Ile725Thr
ClinVar^a^	SCV004809186	SCV004809187	SCV004809188	SCV004809189	SCV004809190	SCV004809191	SCV004812191
rs number (dbSNP)	unreported	unreported	unreported	unreported	unreported	unreported	rs200708403
Allele frequency (gnomAD v.2.1.1)	unreported	unreported	unreported	unreported	unreported	unreported	All: 0.00081% - NFE: 0.0018%
Amino acid conservation	highly conserved	highly conserved	highly conserved	highly conserved	highly conserved	highly conserved	highly conserved
*In silico* prediction tools^b^	SIFT: deleterious, MutationTaster: deleterious, PolyPhen2: possibly damaging, CADD: 28.4, AlphaMissense: 0.9899	SIFT: deleterious, MutationTaster: deleterious, PolyPhen2: probably damaging, CADD: 24.9, AlphaMissense: 0.9982	SIFT: deleterious, MutationTaster: deleterious, PolyPhen2: probably damaging, CADD: 29.4, AlphaMissense: 0.9521	SIFT: deleterious, MutationTaster: deleterious, PolyPhen2: probably damaging, CADD: 27.8, AlphaMissense: 0.9928	SIFT: deleterious, MutationTaster: benign, PolyPhen2: benign, CADD: 19.15, AlphaMissense: 0.4986	SIFT: deleterious, MutationTaster: deleterious, PolyPhen2: possibly damaging, CADD: 25.8, AlphaMissense: 0.6859	SIFT: deleterious, MutationTaster: benign, PolyPhen2: probably damaging, CADD: 27.1, AlphaMissense: 0.9291
Inheritance	*de novo*	*de novo*	*de novo*	*de novo*	*de novo*	AD inheritance with 73% penetrance	inherited from asymptomatic mother
Genetic testing revealing *ANO4* variant	trio WES	trio WES	trio WES	trio WES	trio WES	linkage + WGS	candidate gene screening
Previous genetic testing	chromosomal microarray normal, skewed X-inactivation 15:85	standard karyotype normal, chromosomal microarray normal, epilepsy panel normal	chromosomal microarray normal, epilepsy panel normal	chromosomal microarray normal, karyotype normal, SCN1A sequencing + MLPA normal	chromosomal microarray with paternal duplication of 132 kb in 21q22.3	–	chromosomal microarray normal
Other relevant genetic findings	compound heterozygous variants in *GJB2*c.35del (GenBank: NM_004004.5) and c.101T>C potentially associated with late onset hearing loss	none	–	–	heterozygous variant in *SLC25A22* associated with autosomal recessive DEE	–	inherited heterozygous *GCH1* gene deletion associated with Dopa-responsive dystonia

AD, autosomal dominant; dbSNP, Single-Nucleotide Polymorphism Database; gnomAD, The Genome Aggregation Database; SIFT, sorting intolerant from tolerant; CADD, combined annotation dependent depletion; NFE, non-Finnish European; WES, whole-exome sequencing; WGS, whole-genome sequencing.

a ClinVar accession number of record submitted by the authors.

b SIF, MutationTaster, PolyPhen2, and CADD scores as provided by Alamut Visual Plus v1.7.1 on June 21, 2023 (Sophia Genetics SA, Lausanne, Switzerland).

**Table 3:** Clinical features of individuals with disease-associated *ANO4* variants (I1-7) and a likely benign deletion (DECIPHER ID 272667)

ADL, activities of daily living; ASD, autism spectrum disorder; cMRI, cerebral magnetic resonance imaging; CPAP, continuous positive airway pressure; DEE, developmental and epileptic encephalopathy; EE, epileptic encephalopathy; GEFS+, genetic epilepsy with febrile seizures plus; H, height; ID, intellectual disability; IQ, intelligence quotient; L, length; Mb, megabases; NA, not assessed; NR, not reported; OFC, occipitofrontal circumference; SUDEP, sudden unexplained death in epilepsy; TLE, temporal lobe epilepsy; W, weight.

**Table 4:** Characterization of epilepsy phenotypes associated with *ANO4* variants

AED, antiepileptic drug; CBZ, carbamazepine; CLB, clobazam; CLP, clonazepam; DEE, developmental and epileptic encephalopathy; EE, epileptic encephalopathy; EEG, electroencephalography; GEFS+, genetic epilepsy with febrile seizures plus; GTCS, generalized tonic clonic seizures; LCM, lacosamide; LEV, levetiracetam; LTG, lamotrigine; OXC, oxcarbazepin; PB, phenobarbital; PHT, phenytoin; RFM, rufinamide; STP, stiripentol; TLE, temporal lobe epilepsy; TPM, topiramate; VPA, valproic acid; ZNS, zonisamide.

**Figure 1:**
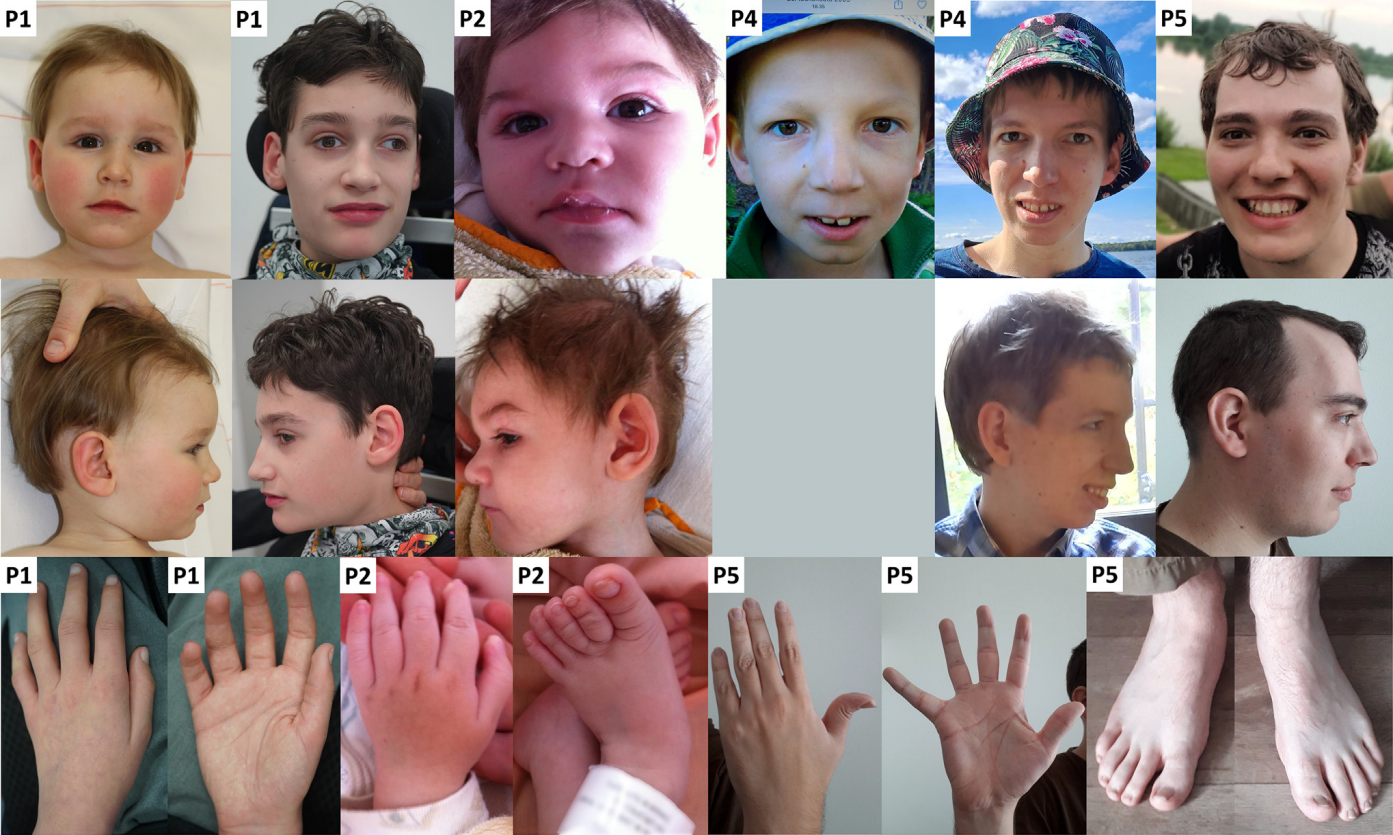
Morphology of individuals with *ANO4* variants Facial appearances of four individuals with *de novo* variants in *ANO4* associated with DEE or EE (I1 at age 12 months and 12 years 2 months, I2 at age 15 months, I4 at age 9 years, 18 years, and 20 years, respectively, and I5 at age 22 years 9 months) and hands/feet of three individuals (I1 at age 12 years 2 months, I2 at age 15 months, and I5 at age 22 years 9 months).

I1–I5 were sporadic cases diagnosed with developmental and epileptic or epileptic encephalopathy (DEE/EE) that harbored the following heterozygous *de novo ANO4* missense variants, respectively: I1, c.1688T>A (p.Met563Lys) (M563K); I2, c.1674C>A (p.Asn558Lys) (N558K); I3, c.1684A>T (p.Ile562Phe) (I562F); I4, c.1807A>G (p.Asn603Asp) (N603D); and I5, c.387C>G (p.Asn129Lys) (N129K). All detected variants are not reported in the Genome Aggregation Database (gnomAD v.2.1.1) and the Single-Nucleotide Polymorphism Database (dbSNP) and affect a highly conserved amino acid (Figure S3). *In silico* prediction tools SIFT (sorting intolerant from tolerant),^74^ MutationTaster,^75^ PolyPhen2,^76^ and AlphaMissense^77^ predict a deleterious or possibly/probably damaging effect for variants detected in I1–I4, while predictions for c.387C>G (p.Asn129Lys) are ambiguous. The phenotype was classified as DEE in I1–I3 and as EE in I4 and I5. Seizure types were variable, including both focal and generalized seizures, and began in the first year of life in individuals I1–I4 and at 41 months of age in I5. Reported seizure-provoking factors were hyperthermia in all and pain, fatigue, or exercise in one individual each. Four of five DEE/EE individuals are not seizure-free despite multiple antiepileptic medications, with a seizure frequency ranging from several per day to several per year. I4 has been seizure-free since age 17 years with valproic acid and topiramate. He previously showed a good response to bromide. Aggravation with carbamazepine occurred in one individual. All five also have mild to severe muscular hypotonia. Cranial MRI revealed nonspecific changes such as atrophy, hypomyelination, or bilateral periventricular leukomalacia in all five individuals with DEE/EE, though initial scans were sometimes reported as normal. Two of five had secondary microcephaly. Hyperkinesia was described in two of five and tremor in one child. Development was profoundly impaired in three of five, with no independent skills in activities of daily living. Language development was also severely impaired in four of five (non-verbal or few words only). I4 showed normal early development with stagnation occurring by the age of 2–3 years. He had moderate ID in formal neuropsychological examinations and was able to perform activities of daily living during childhood, then regressed in early adulthood to severe ID and needing constant supervision. I5 developed age appropriately until about 7 years old and then regressed with increased seizure activity; at age 14, he had an IQ of 51. Motor development was variable, ranging from lack of independent sitting and walking to motor milestones within the normal range. Behavioral difficulties such as autistic features, attention deficit, or aggressiveness were described in two affected individuals. Scoliosis developed in two individuals; a funnel chest was observed in one. Eye anomalies occurred in three of five individuals and included bilateral optic atrophy, strabismus, and hypermetropia. One child had asymmetric hearing loss. Feeding difficulties were reported in two children, one of which required a gastrostomy. One child had hypothyroidism, susceptibility to infection, and increased need for sleep. Two were diagnosed with sleep apnea. There was no specific recognizable morphological phenotype, though shared features included apparent hyper- or hypotelorism, long nose with bulbous tip, and a thin upper lip (Figure 1).

Family 6 (F6) consists of a large pedigree of 23 affected individuals with TLE or GEFS+ (Figure S2) in which the heterozygous *ANO4* variant c.1582G>A (p.Val528Met) (V528M) was segregating in an autosomal-dominant manner. One individual had only febrile seizures, 11 only afebrile seizures, and ten had a combination of febrile and afebrile seizures. Most affected family members had focal onset seizures, and ten had clear TLE. Two had generalized epilepsy with generalized epileptic discharges on electroencephalography (EEG). Although the family was at that time reported as having familial TLE with febrile seizures, nowadays it would be classified as GEFS+ given the presence of different seizure types within the same family. Three family members died of prolonged febrile seizures, and one of sudden unexplained death in epilepsy (SUDEP). The identified segregating ANO4 variant was present in all 20 affected family members for whom DNA was available and had a 73% penetrance (22 affected [obligate] carriers/30 total [obligate] carriers). I7 had normal neurodevelopment and adult-onset TLE. The heterozygous variant c.2174T>C (p.Ile725Thr) (I725T) was inherited from the unaffected mother who had a maternal uncle who died of epilepsy at the age of 16 years. Maternal grandparents were not available for further segregation testing.

The individual with DECIPHER ID 272667 harbored the 307.65-kb-sized deletion (g.101184062-101491712 [GenBank: NC_000012.11] [GRCh37]) affecting exons 1–19 of 28 of *ANO4* (GenBank: NM_001286615.1), likely leading to a complete loss of allele product. This deletion was inherited from the healthy father, and therefore it was considered of unknown significance for the individual’s phenotype. After an uneventful pregnancy and postnatal period, the individual showed developmental delay and was diagnosed with autism spectrum disorder. The child remained nonverbal at age 7 years. There is no history of seizures, and growth parameters were normal.

### Homology modeling of ANO4 variants

**Figure 2:**
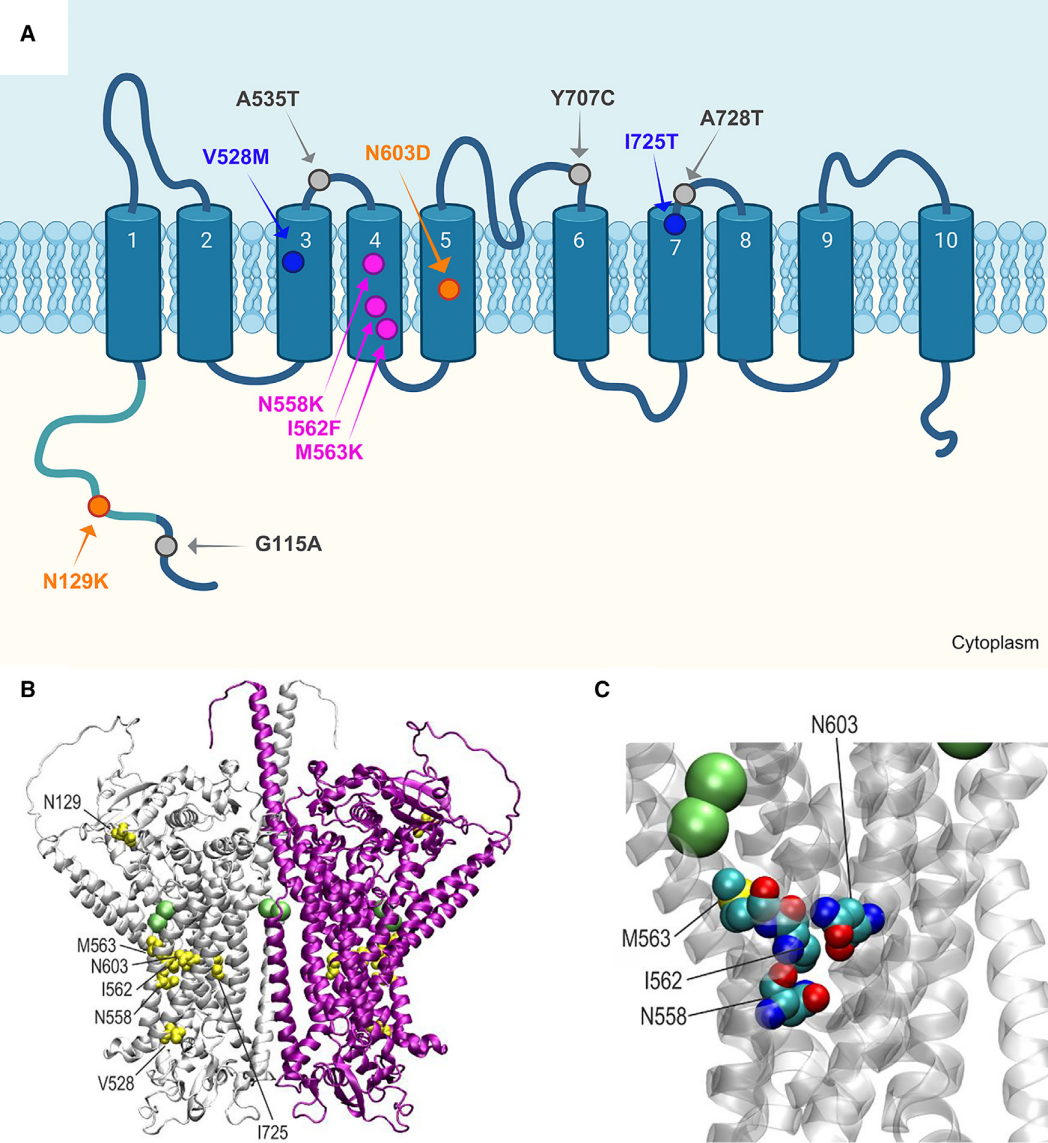
Membrane topology and 3D structure of ANO4 indicating the disease-associated variants and selected population variants (A) Membrane topology of ANO4 based on experimental data in the *N. haematococca* ortholog, drawn using Biorender software. The TMs numbered from 1 to 10 are represented with barrels and located in the plasma membrane. The N-terminal dimerization domain is labeled in turquois. The disease-associated variants are colored according to the phenotype: Asn558Lys (N558K), Ile562Phe (I562F), Met563Lys (M563K) in red with DEE; Asn603Asp (N603D) and Asn129Lys (N129K) in orange with EE; and Val528Met (V528M) and Ile725Thr (I725T) in blue with TLE/GEFS+. The most frequent population variants with deleterious *in silico* predictions Gly115Ala (G115A), Ala535Thr (A535T), Tyr707Cys (Y707C), and Ala728Thr (A728T) are depicted in gray. (B) Overall structure of the ANO4 dimer with the subunits shown as white and purple ribbon. Residues affected by mutation are shown in yellow space-filled presentation and are labeled for one subunit. The position of bound Ca^2+^ ions is indicated by green balls. The disordered residues 1–40 have been omitted for clarity. (C) Enlargement of the ANO4 region that represents a mutational hotspot in the present study. The residues affected by mutation are shown in space-filled representation (atom-type coloring) and are labeled. The position of adjacent Ca^2+^ ions is indicated by green balls.

Mapping of the variants according to the published experimentally determined transmembrane domains (TMs)^5^ is provided in Figure 2A. To better understand the effects of the missense variants on ANO4, we performed a structural protein analysis. Since no experimental structure of ANO4 is available yet, we used a model generated by AlphaFold-2 for the structural interpretation of the variants. All seven variants discovered in the present study are located in the globular part of ANO4 (Figure 2B), and our analysis suggests that they destabilize the ANO4 structure. The most prominent effects involve DEE/EE variants and are steric clashes due to the appearance of larger sidechains (e.g., Ile562Phe, Asn129Lys, Asn558Lys) and/or the emergence of novel charges (e.g., Met563Lys, Asn129Lys, Asn558Lys, Asn603Asp). Of note, four DEE/EE variants (Asn558Lys, Ile562Phe, Met563Lys, and Asn603Asp) cluster in the immediate spatial proximity of a Ca^2+^ ion binding site (Figures 2B and 2C).

### Functional studies

The results of functional studies are summarized in Table 5.

**Table 5:** Summary of results of functional studies

**Individual**	**ANO4 mutant**	**Phenotype**	**Heterogenic expression analysis**					
			**Basal conductance**	**Ca^2+^-dependent cation conductance**	**Membrane location**	**Colocalization with EEA1**	**Apoptosis**	
							**– Ionomycin**	**+ Ionomycin**
I1	Met563Lys	DEE	ns	⬇∗∗∗∗	⬇∗	⬇∗∗∗∗	⬆∗	ns
I2	Asn558Lys	DEE	ns	⬇∗∗∗∗	⬇∗∗∗∗	⬇∗∗∗∗	⬆∗∗	ns
I3	Ile562Phe	DEE	⬇∗	⬇∗∗∗∗	⬇∗∗∗∗	⬇∗∗∗	⬆∗∗	ns
I4	Asn603Asp	EE	ns	⬇∗∗∗∗	⬇∗∗∗∗	⬇∗∗∗∗	ns	ns
I5	Asn129Lys	EE	ns	⬇∗∗∗∗	⬇∗∗∗∗	⬇∗∗∗∗	ns	ns
F6	Val528Met	GEFS+	ns	⬇∗∗∗∗	ns	⬇∗∗∗	ns	ns
I7	Ile725Thr	TLE	⬇∗∗	⬇∗∗∗∗	ns	ns	ns	ns
I2	WT/Asn558Lys	DEE	⬇∗∗	⬇∗∗∗∗	ns	⬇∗∗∗∗	⬆∗∗∗∗	not tested
F6	WT/Val528Met	GEFS+	⬇∗∗∗	⬇∗∗∗∗	⬇∗∗∗	⬇∗∗∗∗	⬆∗∗∗	not tested

DEE, developmental and epileptic encephalopathy; EE, epileptic encephalopathy; EEA1, early endosome antigen 1; GEFS+, genetic epilepsy with febrile seizures plus; ns, not significant; TLE, temporal lobe epilepsy; WT, wild-type**.** ∗p **<** 0.05; ∗∗*p* < 0.01, ∗∗∗*p* < 0.001, ∗∗∗∗*p* < 0.0001.

### Mutant ANO4 show reduced Ca^2+^-dependent cation conductance and plasma membrane localization

**Figure 3:**
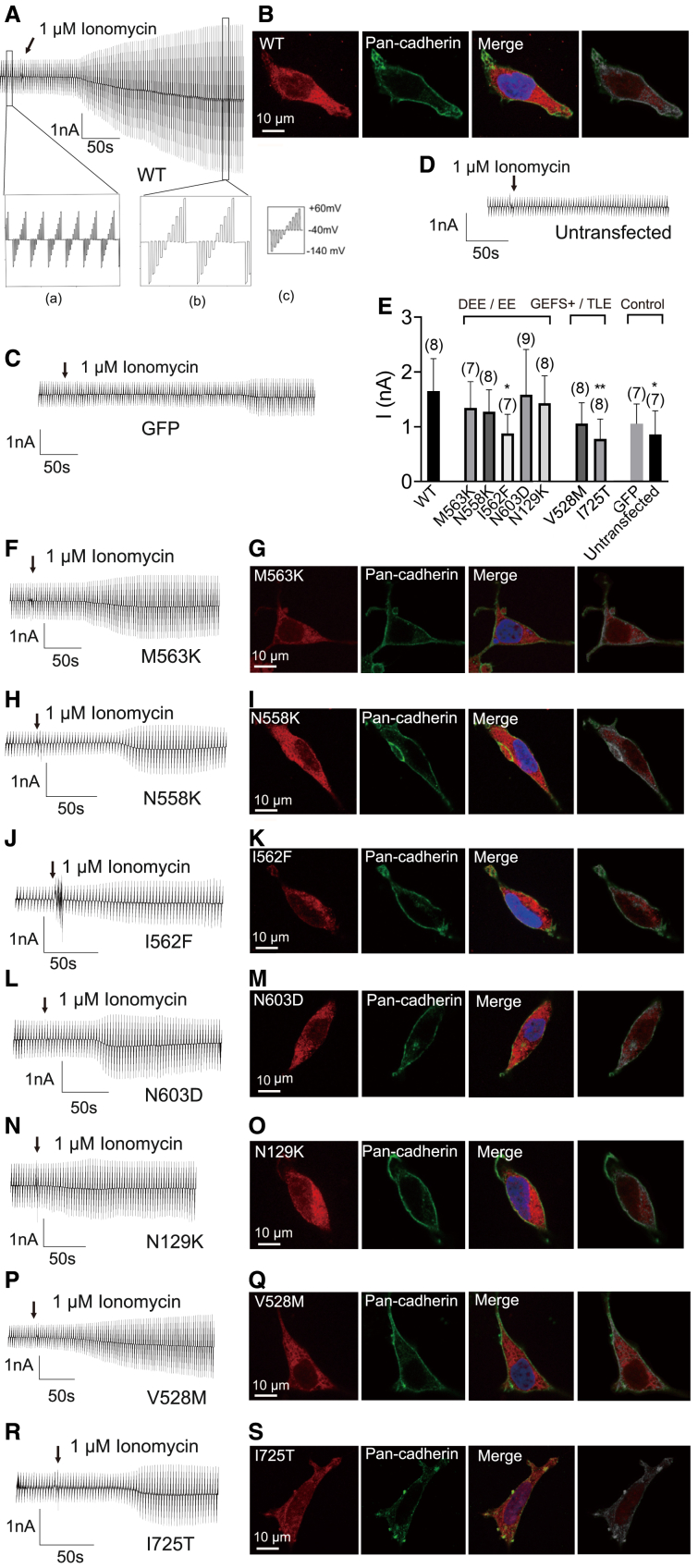
Analysis of the Ca^2+^-dependent cation conductance of heterologously expressed wild-type and mutant ANO4 (A) Raw current recording in HEK293 cells expressing wild-type ANO4 before and after application of ionomycin (1 μM; arrow); (a): the baseline currents before ionomycin application, (b): the currents after the application of 1 μM ionomycin, (c): stimulation protocol with 10 voltage steps between −140 mV and +60 mV, for a duration of 50 ms applied every 2.5 s. (B) Immunostaining of a HEK293 cell expressing wild-type ANO4 against ANO4 (red) and the membrane marker pan-cadherin (green). From the left panel: ANO4 only, pan-cadherin only, merged ANO4 and pan-cadherin, and display of pixels that co-localize red and green in white. (C and D) Control experiments using cells expressing GFP (C) and untransfected cells (D). Raw currents before and after application of ionomycin (arrow). (E, F, H, J, L, N, P, and R) Summary of maximal current amplitudes estimated from a voltage difference between −120 mV and +60 mV measured before application of ionomycin to compare basal membrane conductance between controls, wild-type ANO4, and seven mutant ANO4. Raw currents measured in a cell expressing ANO4 mutants Met563Lys (M563K; F), Asn558Lys (N558K; H), Ile562Phe (I562F; J), Asn603Asp (N603D; L), Asn129Lys (N129K; N), Val528Met (V528M; P), or Ile725Thr (I725T; R) before and after ionomycin application (arrow). (G, I, K, M, O, Q, and S) Immunostaining of a HEK293 cell expressing ANO4 mutants Met563Lys (M563K; G), Asn558Lys (N558K; I), Ile562Phe (I562F; K), Asn603Asp (N603D; M), Asn129Lys (N129K; O), Val528Met (V528M; Q), or Ile725Thr (I725T; S) with antibody against ANO4 (red) and the membrane marker pan-cadherin (green). From the left panel: ANO4 only, pan-cadherin only, merged ANO4 and pan-cadherin, and display of pixels that co-localize red and green in gray. Values are given as mean ± SEM. Multiple comparisons were performed by ANOVA with Dunn’s post hoc test. Data points that were statistical outliers were eliminated by the Grubbs outlier test. ^∗^*p* < 0.05; ^∗∗^*p* < 0.01; *n* is given in parentheses. DAPI was used to counterstain the nucleus (blue) in (B, G, I, K, M, O, Q, and S).

To investigate whether disease-associated *ANO4* variants alter Ca^2+^-dependent cation conductance, whole-cell patch-clamp recordings were performed on HEK293 cells that heterologously express either wild-type ANO4 or one of the ANO4 mutants. To ignite Ca^2+^-dependent cation membrane conductance, intracellular free Ca^2+^ was increased by the extracellular application of the Ca^2+^ ionophore ionomycin (1 μM). In the first step, we verified the basal properties of wild-type ANO4 and transfection controls (Figures 3A–3C, and 3D). Cells overexpressing wild-type ANO4 showed a robust current activation after application of 1 μM ionomycin (Figure 3A). In control experiments with GFP-transfected cells (Figure 3C) or untransfected cells (Figure 3D), we saw weak activation of currents after ionomycin application that were, however, much smaller than those with wild-type ANO4. In the second step, we investigated the Ca^2+^-dependent cation membrane conductance in cells transfected with ANO4 mutants. Thereby, we noted that the baseline of current amplitude was significantly lower in mutants Ile562Phe and Ile725Thr compared to wild-type (*p* < 0.05) and similar to the control baselines. The other ANO4 mutants showed no significant difference in the baseline of current amplitude between the membrane potentials of −140 mV to +60 mV (Figure 3E). After application of ionomycin, all ANO4 mutants showed a relatively mild increase in current amplitude with an increased membrane conductance after a latency of approximately 100 s that turned the holding current at −40 mV into an inward current corresponding with cell depolarization and weak or no rectification (Figures 3F–3H, 3J, 3L, 3N, 3P, and 3R). Given our experimental conditions with a difference of extracellular Cl^−^ (145 mM) and intracellular Cl^−^ (30 mM), these findings correspond with the activation of a non-selective cation current because activation of Cl^−^ channels would not change the holding current (holding potential equals the Nernst potential for Cl^−^) and would show outward rectification.

**Figure 4:**
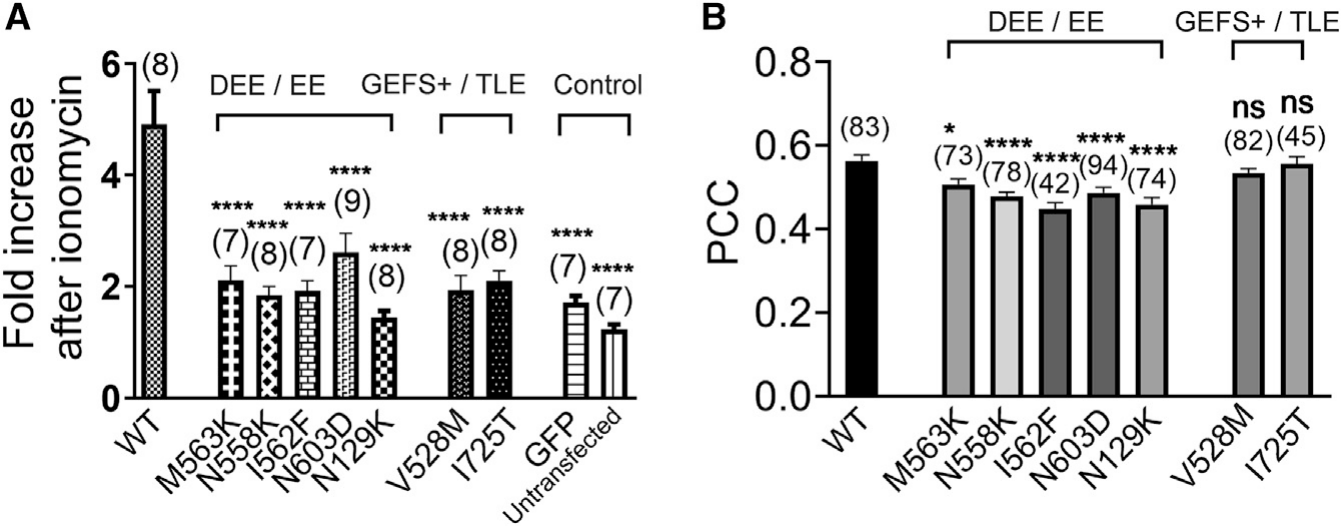
Comparison of ANO4-dependent membrane conductance and ANO4 surface expression (A) Comparison of changes in membrane conductance estimated as currents of a voltage difference between −140 mV and +60 mV from cells with different conditions of heterologous expression after reaching a maximal steady-state level of the ionomycin effect, given as fold increase from baseline. (B) Comparison of Pearson’s correlation coefficient (PCC) of ANO4 and pan-cadherin positive pixels from immunostainings of HEK293 cells expressing ANO4 wild-type or mutants Met563Lys (M563K), Asn558Lys (N558K), Ile562Phe (I562F), Asn603Asp (N603D), Asn129Lys (N129K), Val528Met (V528M), or Ile725Thr (I725T). Values are given as mean ± SEM. Multiple comparisons were performed by ANOVA with Dunn’s post hoc test. Data points that were statistical outliers were eliminated by the Grubbs outlier test. ^∗^*p* < 0.05; ^∗∗∗∗^*p* < 0.0001; ns, not significant; *n* is given in parentheses; in (B), *n* gives the number of analyzed cells from three independent experiments.

For quantitative statistical comparison, we estimated the amplitude of ionomycin-induced steady-state currents and calculated the fold current increase in response to ionomycin (Figure 4A). Ionomycin application resulted in a 1- to 2.5-fold increase in current density in all ANO4 mutants, which was significantly lower compared to the 5-fold increase in wild-type ANO4 (*p* < 0.0001) (Figure 4A) suggesting a loss of channel function for all mutants. Of note, ANO4 mutant Asn603Asp showed the smallest decrease in current density of all mutants to about half of that of wild-type ANO4.

**Figure 5:**
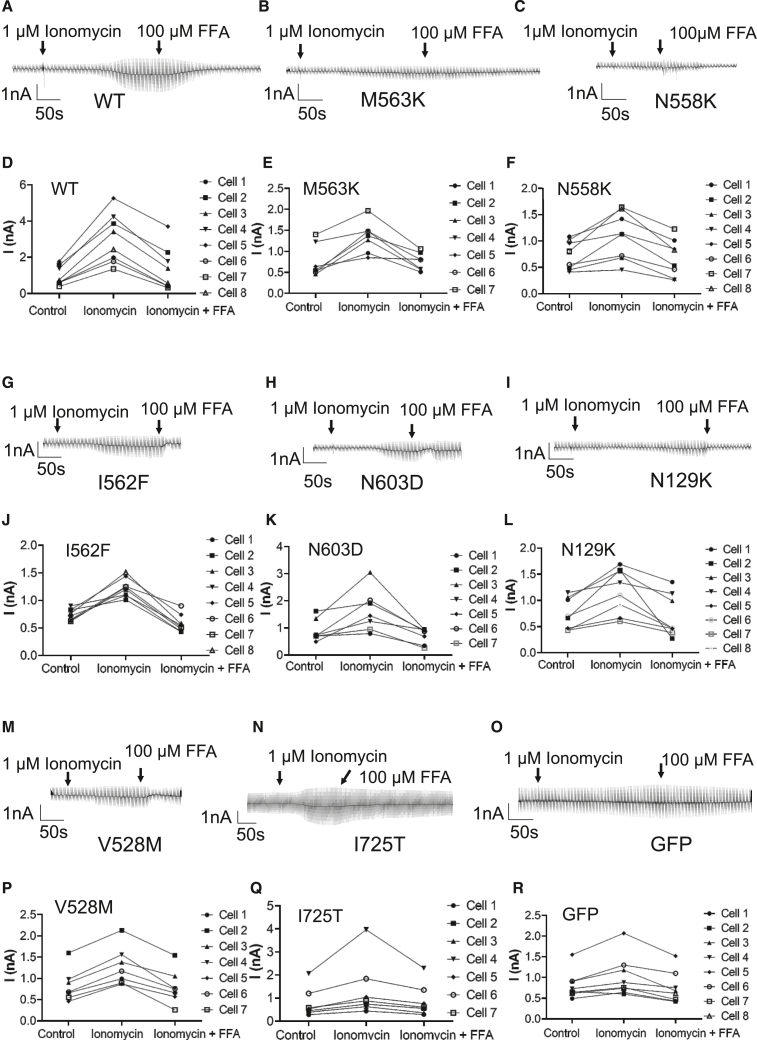
The Ca^2+^-dependent cation current elicited by heterologously expressed mutant ANO4 is blocked by the application of the flufenamic acid (A–C, G–I, and M–O) When ionomycin-mediated current density of HEK293 cells expressing wild-type or mutant ANO4 reached its peak, the flufenamic acid (100 μM) was applied to the bath solution (arrows). Raw current recordings of cells before and after the application of ionomycin and flufenamic acid (FFA) are shown. The HEK293 cells over-expressing wild-type ANO4 (A) showed increased current density after extracellular application of 1 μM ionomycin, which can be blocked by 100 μM FFA; the same result can be observed in the cells expressing ANO4 with the mutations Met563Lys (M563K; B), Asn558Lys (N558K; C), Ile562Phe (I562F; G), Asn603Asp (N603D; H), Asn129Lys (N129K; I), Val528Met (V528M; M), and Ile725Thr (I725T; N). GFP alone transfected cells in (O). (D–F, J–L, and P–R) X axis indicates the baseline, presence of ionomycin and flufenamic acid; y axis shows the raw currents in nanoampere (nA). Effects of ionomycin and flufenamic acid (FFA) on each cell can be seen. The effect plots from HEK293 cells expressing ANO4 wild-type (D), ANO4 mutant Met563Lys (M563K; E), Asn558Lys (N558K; F), Ile562Phe (I562F; J), Asn603Asp (N603D; K), Asn129Lys (N129K; L), Val528Met (V528M; P), Ile725Thr (I725T; Q), and cells transfected with GFP alone (R).

To confirm that these results are indeed due to anoctamin dysfunction, we applied flufenamic acid (100 μM), a broad range blocker for ANOs, to the extracellular solution once the currents reached a steady-state value in response to 1 μM ionomycin. The ionomycin-activated currents of both wild-type and mutant *ANO4* transfected cells were sensitive to flufenamic acid, indicating that the observed currents depend on ANOs (Figure 5).

To investigate whether the observed loss-of-function (LoF) effect occurs through malfunction of the pore or reduced trafficking into the plasma membrane, we assessed plasma membrane trafficking using immunocytochemistry (Figures 3B–3G, 3I, 3K, 3M, 3O, 3Q, 3S and S4). As expected, wild-type ANO4 was detected at the plasma membrane as indicated by co-localization with pan-cadherin, resulting in a PCC of 0.568 (Figure 4B). This indicates that 57% of wild-type ANO4 is located at the plasma membrane, and a large proportion was present in the cytosol. Contrarily, the proportion of plasma membrane localization of DEE/EE-associated ANO4 mutants (Met563Lys, Asn558Lys, Ile562Phe, Asn603Asp, and Asn129Lys) was significantly reduced (Figure 4B). Notably, ANO4 mutants Val528Met and Ile725Thr, associated with the less-severe phenotypes of GEFS+ and TLE, didn’t show any significant difference to the wild type (Figures 4B and S4).

**Figure 6:**
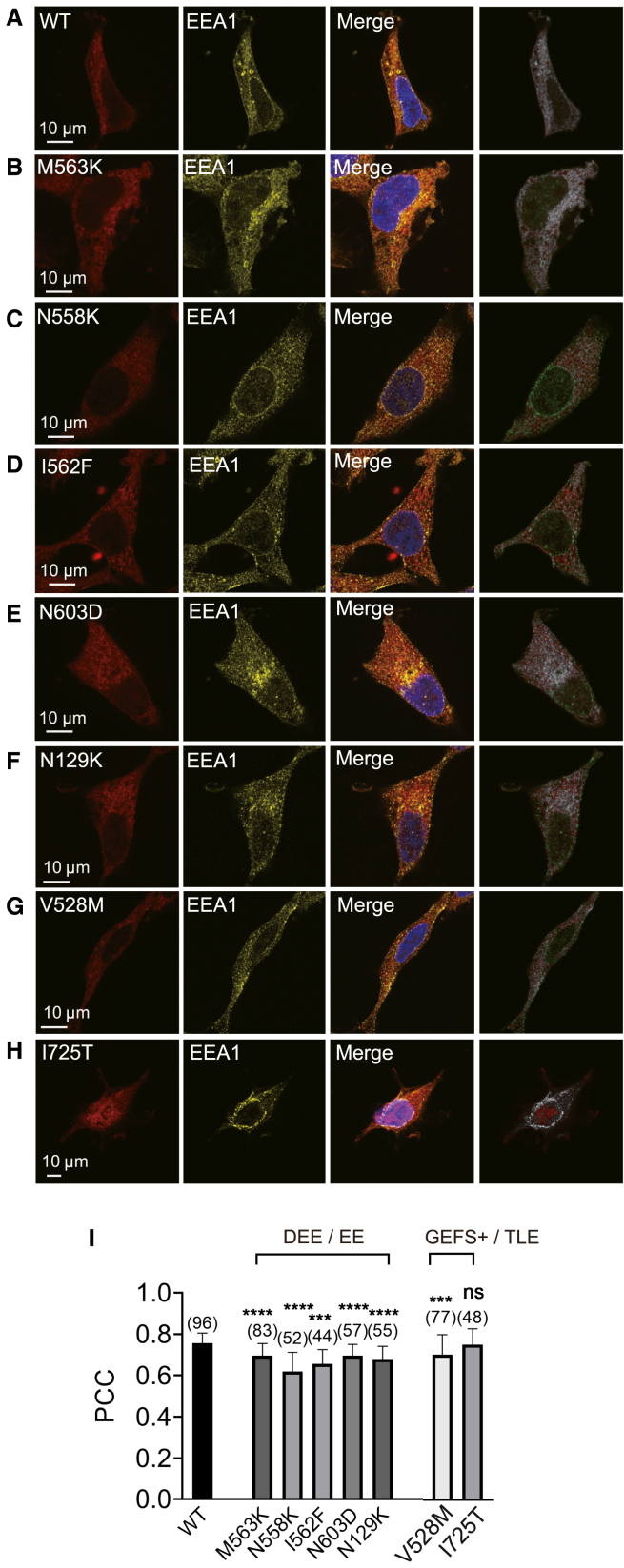
Co-localization of wild-type and mutant ANO4 with the early endosome marker EEA1 in HEK293 cells (A–H) HEK293 cells expressing wild-type (A) and various mutated ANO4 constructs were stained with antibodies for ANO4 (left, red) and EEA1 (left-middle, yellow). Merged images (right-middle) show the co-localization of mutant ANO4 and EEA1. The gray dot images (right) represent the density of co-localization of mutant ANO4 and EEA1. Nuclei were stained with DAPI. ANO4 mutants included Met563Lys (M563K; B), Asn558Lys (N558K; C), Ile562Phe (I562F; D), Asn603Asp (N603D; E), Asn129Lys (N129K; F), Val528Met (V528M; G) and Ile725Thr (I725T; H). Scale bar represents 10 μm. (I) PCC analysis of EEA1 and mutant ANO4 (transfection and immunostaining according to A–H). The number inside the parentheses represents n per group. Whiskers represent SEM. Values are given as mean ± SEM. Multiple comparisons were performed by ANOVA with Dunn’s post hoc test. Data points that were statistical outliers were eliminated by the Grubbs outlier test. ^∗∗∗^*p* < 0.001; ^∗∗∗∗^*p* < 0.0001; ns, not significant; the number in parentheses represents the number of analyzed cells from three independent experiments.

To investigate whether ANO4 variants affect subcellular trafficking, co-localization with early endosomes was assessed using the early endosome marker EEA1 (Figures 6A–6H). All ANO4 mutants except Ile725Thr showed significantly reduced co-localization with EEA1 compared to wild-type ANO4 (*p* < 0.0001; Figures 6I and S4). Thus, the lower surface expression of mutant ANO4 correlates with reduced trafficking through early endosomes and therefore does not suggest aberrant clogging as pathomechanism.

### Assessment of phospholipid scramblase activity by annexin A5 surface expression in HEK293 cells overexpressing wild-type or mutant ANO4

**Figure 7:**
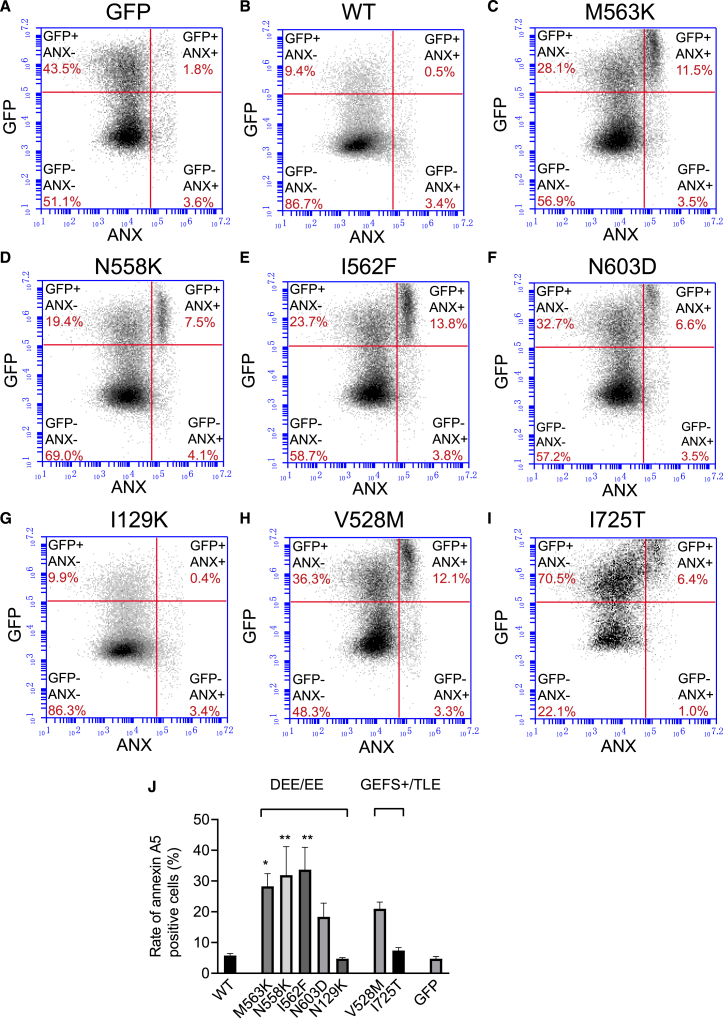
Scramblase activity in HEK293 cells expressing wild-type or mutant ANO4 at physiological Ca^2+^ levels (A–I) Scramblase activity was assessed by FACS sorting of annexin A5-labeled HEK293 cells that were not treated by ionomycin and transfected with GFP alone (A), with wildtype ANO4 plus GFP (B) and ANO4 mutants plus GFP under control conditions. ANO4 mutants included Met563Lys (M563K; C), Asn558Lys (N558K; D), Ile562Phe (I562F; E), Asn603Asp (N603D; F), Asn129Lys (N129K; G), Val528Met (V528M; H), and Ile725Thr (I725T; I). X axis indicates fluorescence intensity of Anx A5-6S-IDCC (log); y axis indicates fluorescence intensity of GFP (log). The right-upper square represents the ANO4 transfected, annexin A5-positive cell fraction. (J) Comparison of annexin A5 surface expression between different transfection conditions. The experiments were carried out four times (*n* = 4). Values are given as mean ± SEM. Multiple comparisons were performed by ANOVA with Dunn’s post hoc test. Data points that were statistical outliers were eliminated by the Grubbs outlier test. ^∗^*p* < 0.05; ^∗∗^*p* < 0.01.

Since ANO4 may also have phospholipid scramblase activity, we investigated binding of fluorescently labeled annexin A5 to exposed PS. An increased surface expression of PS can indicate activated apoptosis in stressed cells. At physiological levels of intracellular Ca^2+^, cells expressing Met563Lys, Asn558Lys, and Ile562Phe showed significantly increased annexin A5 surface binding compared to wild-type ANO4 (Figures 7C, 7D, 7E, 7F, 7H, and 7J), suggesting higher rates of apoptosis. There was no significant difference in annexin A5 binding between mutants Asn603Asp, Val528Met, Asn129Lys, and Ile725Thr and wild-type ANO4 or GFP (Figures 7A, 7B, 7G, 7I, and 7J).

**Figure 8:**
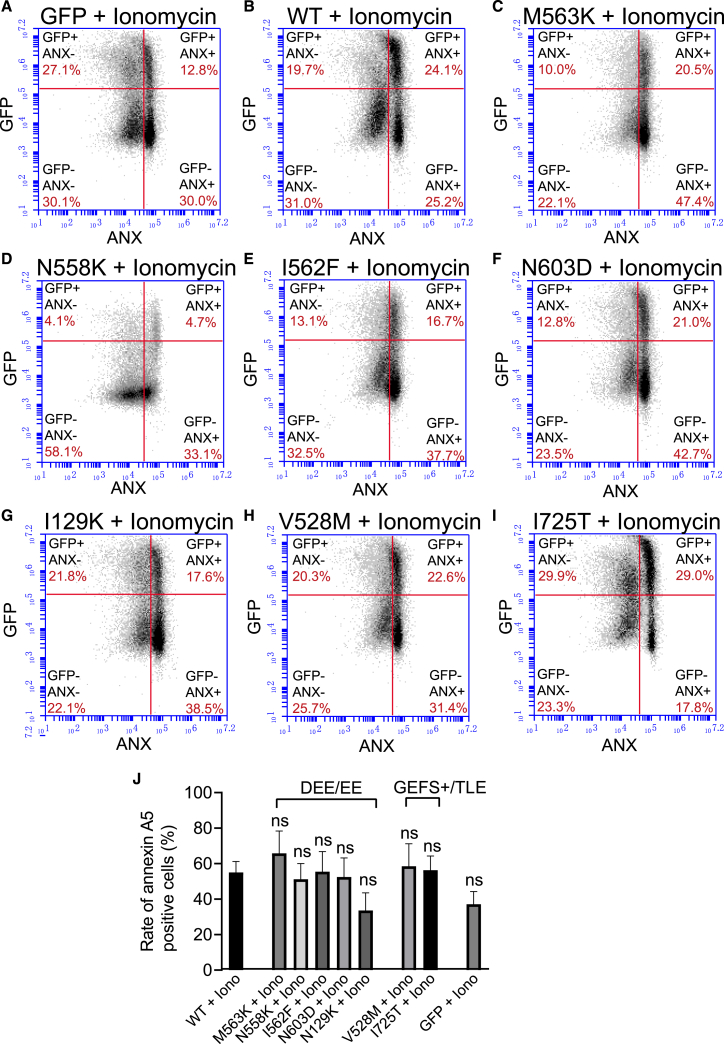
Ca^2+^-dependent scramblase activity in HEK293 cells expressing wild-type or mutant ANO4 Ca^2+^-dependent scramblase activity was assessed by FACS sorting of annexin A5-labeled HEK293 cells that were incubated with ionomycin (Iono, 1 μM) for 10 min and prior to the experiment transfected with GFP alone (A), with wild-type ANO4 plus GFP (B), and ANO4 mutations plus GFP: Met563Lys (M563K; C), Asn558Lys (N558K; D), Ile562Phe (I562F; E), Asn603Asp (N603D; F), Asn129Lys (N129K; G), Val528Met (V528M; H), and Ile725Thr (I725T; I). X axis indicates fluorescence intensity of annexin A5-6S-IDCC (log); y axis indicates fluorescence intensity of GFP (log). The right-upper square represents the ANO4 transfected, annexin A5-positive cell fraction. (J) Comparison of annexin A5 surface expression between different transfection conditions. The experiments were carried out four times (*n* = 4). Values are given as mean ± SEM. Multiple comparisons were performed by ANOVA with Dunn’s post hoc test. Data points that were statistical outliers were eliminated by the Grubbs outlier test. ns, not significant

To evaluate Ca^2+^-dependent scramblase activity, annexin A5 binding was further examined under the condition of increased intracellular Ca^2+^. For this purpose, transfected cells were treated with 1 μM ionomycin for 10 min prior to measurement. There was no significant difference in annexin A5 surface binding between ANO4 wild-type and mutants compared to cells transfected with GFP only (Figures 8A–8J), indicating absence of Ca^2+^-dependent scramblase activity of wild-type or mutant ANO4.

### Wild-type/mutant co-transfection experiments

**Figure 9:**
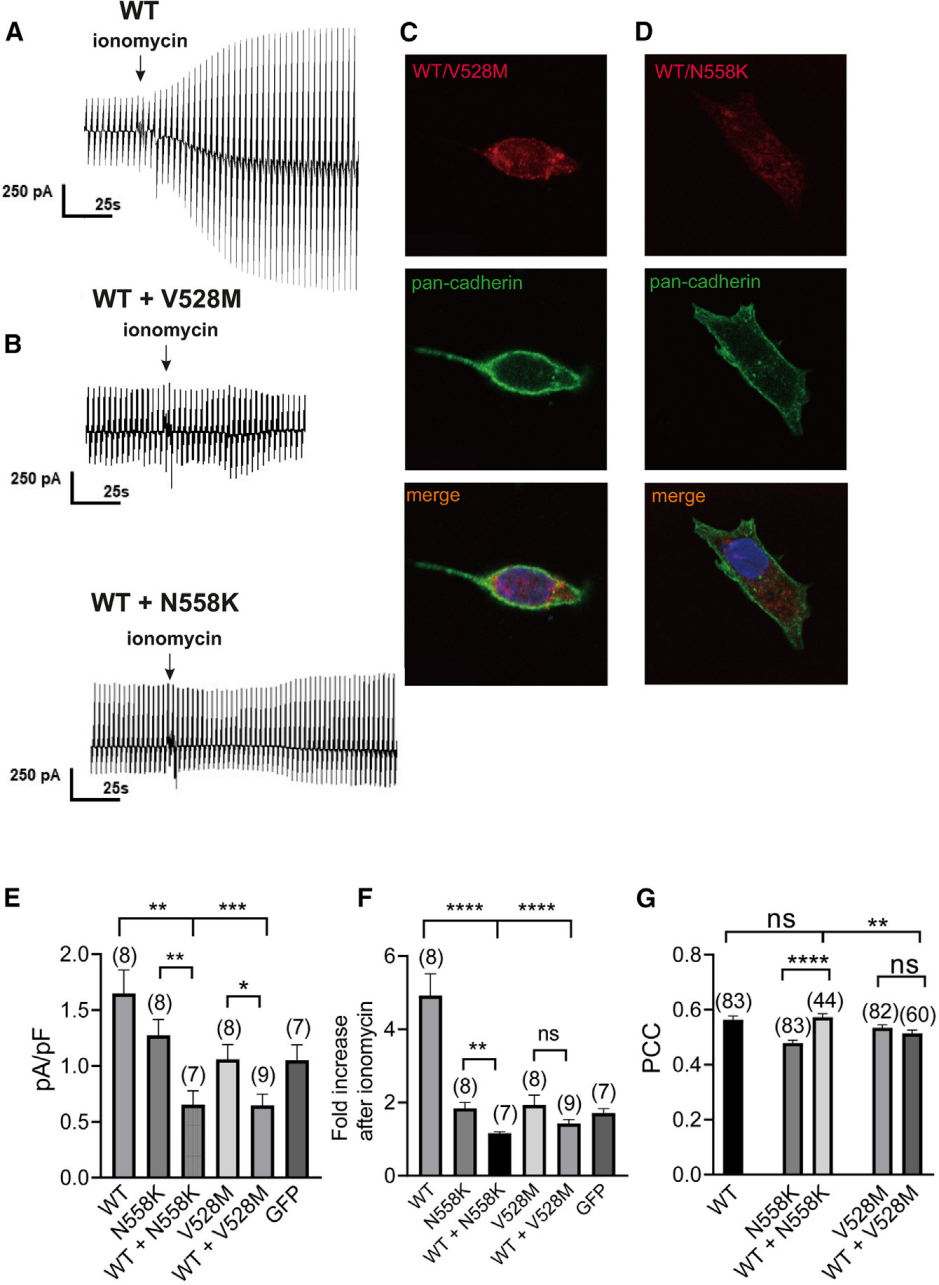
ANO4 currents and ion channel surface expression in co-expression experiments HEK293 cells were co-transfected with either wild-type and DEE mutant Asn558Lys (N588K) or wild-type and GEFS+ mutant Val528Met (V528M) ANO4 and compared to wild-type or mutant transfection alone. (A and B) (A) Raw current recording in HEK293 cells expressing wild-type ANO4 before and after application of ionomycin (1 μM; arrow) and (B) in wild-type/Val528Met and wild-type/Asn558Lys co-expression. (C and D) Immunostaining of a HEK293 cell co-expressing wild-type/Val528Met or wild-type/Asn558Lys against ANO4 (red) and the membrane marker pan-cadherin (green); from the top: ANO4 only, pan-cadherin only, merged ANO4 and pan-cadherin. (E and F) (E) Comparison of peak current densities at physiological Ca^2+^ conditions from cells with different transfection paradigm and (F) changes in membrane conductance after reaching a maximal steady-state level of the ionomycin effect; given as fold increase from baseline. (G) Comparison of Pearson’s correlation coefficient (PCC) of ANO4 and pan-cadherin-positive pixels from immunostainings of HEK293 cells expressing wild-type alone, mutant alone, or co-expressing wild-type/Val528Met or wild-type/Asn558Lys. Values are given as mean ± SEM. Multiple comparisons were performed by ANOVA with Dunn’s post hoc test. Data points that were statistical outliers were eliminated by the Grubbs outlier test. *n* is given in parentheses. ^∗^*p* < 0.05; ^∗∗^*p* < 0.01; ^∗∗∗^*p* < 0.001; ∗∗∗∗*p* < 0.0001; ns, not significant.

In order to investigate a potential dominant-negative effect, we performed additional heterologous expression experiments in which we co-expressed both wild-type and mutant ANO4. For that purpose, we transfected HEK293 cells with a 50/50 combination of wild type with either the DEE-associated Asn558Lys or the GEFS+-associated Val528Met ANO4 mutant and analyzed the functional properties by patch-clamp analysis (Figures 9A, 9B, and 9E), surface expression of ANO4 proteins using immunocytochemistry (Figures 9C, 9D and 9G), early endosome co-localization (Figure S5), and annexin A5 surface expression (Figure S6). In contrast to transfection with mutants only, basal membrane conductance was significantly smaller in cells that had been transfected with wild-type/mutant combinations compared to wild type alone (Figure 9E). Similar to the mutants alone, the Ca^2+^-induced cation conductance was significantly lower in cells with wild-type/mutant co-expression than that of wild-type ANO4 transfection. Regarding cell membrane surface expression, the co-transfected cells showed no difference between Val528Met and wild-type/Val528Met, whereas wild-type/Asn558Lys showed a small increase in ANO4 surface expression compared to Asn558Lys. We also observed small but significant differences in early endosome localization with wild-type/Asn558Lys showing increased and wild-type/Val528Met showing decreased levels of co-localization compared to mutant alone (Figure S5). However, given the small differences, we are cautious to consider these as biologically relevant. Scramblase activity measured by annexin A5 surface expression was similar in the co-transfection and single-transfection paradigm (Figure S6). In summary, we found evidence for a dominant-negative effect since the wild-type protein could not alleviate the deleterious effects of mutant ANO4 in co-expression experiments.

## Discussion

We identified *de novo* heterozygous missense variants in *ANO4* as a cause of fever-sensitive DEE/EE as well as autosomal dominantly inherited missense variants underlying familial GEFS+ and TLE with reduced penetrance.

All individuals of our cohort exhibiting *ANO4*-associated DEE/EE (I1–I5) first presented to the clinic with neurodevelopmental delay or epileptic seizures. Epilepsy was mostly of infantile onset, intractable, and included both generalized (tonic clonic, tonic, clonic, myoclonic, absences) and focal seizures. Fever-sensitivity was present in all. Interestingly, two recent genome-wide association studies have identified common variants near the *ANO3* (or *TMEM16C*) locus as a strong risk factor for febrile seizures.^78^^,^^79^ Three individuals (I1–I3) displayed severe to profound impairment in psychomotor development from birth, whereas two individuals (I4–I5) showed initially normal development before regression to moderate to severe ID. They were therefore clinically classified as EE, but we acknowledge that it is challenging to distinguish a potential developmental impact of the underlying variant from detrimental effects of seizure activity on a vulnerable brain, and we assume that both likely contribute to the regression. All individuals display muscular hypotonia and non-specific anomalies in brain imaging. Other clinical features of this condition are variable and encompass secondary microcephaly and various neurological issues such as hyperkinesia or tremor. Additionally, some individuals exhibit feeding difficulties, eye anomalies (e.g., optic atrophy, strabismus, hypermetropia), severe scoliosis, hearing loss, sleep disturbances, hypothyroidism, and increased susceptibility to infections.

Additionally, we observed two inherited missense variants associated with non-syndromic GEFS+ in F6 or TLE in I7 with reduced penetrance and normal psychomotor development. F6 represents a large autosomal dominant GEFS+ pedigree in which we found the *ANO4* missense variant as the sole qualifying variant in the linked disease locus that was present in all 20 tested affected individuals and a disease penetrance of 73% (Figure S2). Of note, several members of F6 died of prolonged seizures or SUDEP, emphasizing that despite the normal psychomotor development, this is still a severe disorder. Strikingly, cortical dysplasia was detected on brain MRI in two members of this large family, as well as in I3. While cortical malformations have been previously reported in several ion channel disorders,^80^ further investigation is necessary to assess their potential relationship to ANO4 dysfunction.

In contrast, the complete loss of one *ANO4* allele in an individual with autism and ID and the healthy father (DECIPHER ID 272667) does not cause any seizure phenotype. Furthermore, constraint metrics in gnomADv2.1.1 show some depletion of LoF variants (63.8 expected predicted loss-of-function [pLoF] variants vs. 19 observed; loss-of-function observed/expected upper bound fraction [LOEUF] = 0.44), but a probability of being LoF intolerant score (pLI) of 0 and no homozygous LoF variants. We therefore assume a variant-specific *ANO4* dysfunction rather than haploinsufficiency as pathomechanism in I1–I7 and hypothesize that haploinsufficiency of *ANO4* is not a cause of an apparent developmental disorder.

Functional analysis of cation conductance by whole-cell patch-clamp experiments demonstrated that all *ANO4* variants identified in our study cohort exhibited a significant reduction of Ca^2+^-dependent cation conductance, with Asn558Lys, Ile562Phe, and Asn129Lys leading to a complete loss of ANO4-mediated cation conductance (Figures 3 and 4). The validity of this finding is supported by our earlier work, where we studied the putative cation selectivity of ANO4 by exchanging negatively charged conserved amino acids in the pore region with positively charged ones and found no LoF.^61^ The sensitivity to inhibition by flufenamic acid supports that the measured currents indeed originate from anoctamin proteins (Figure 5). Immunostainings and co-localization analyses with a plasma membrane marker showed slightly reduced plasma membrane localization (Figures 3 and 4) and reduced early endosome localization of all five DEE/EE-associated ANO4 mutants (Figure 6). This finding indicates that reduced ANO4 membrane trafficking contributes to the loss of ion channel function in the *ANO4* variants associated with the severe encephalopathic phenotype (I1–I5). The two variants associated with the inherited epilepsy phenotypes (F6 and I7) did not show reduced plasma membrane localization. Of note, a considerable proportion of the wild-type as well as mutant protein appeared to be localized within the cytoplasm. Similar observations have been reported in previous studies and were interpreted to imply its intracellular function with relevance for Ca^2+^ store filling processes.^81^ However, we propose that the detected disease-associated variants primarily affect the ion channel pore function of ANO4 since ionic currents were reduced to 40% or less compared to the wild type, while the surface expression was normal or mildly reduced to 90% only (Figure 4).

Furthermore, we examined scramblase activity of ANO4 and its mutants by quantifying surface expression of annexin A5, which binds exposed PS with high affinity and is therefore used as a marker of apoptosis. ANO4 wild-type expression did not substantially change scramblase activity, neither in physiological nor in elevated Ca^2+^ conditions, consistent with our previous findings.^61^ However, all three DEE mutants (Asn558Lys, Ile562Phe, Met563Lys) displayed increased apoptosis at resting conditions (Figure 7) but not at increased Ca^2+^-levels (Figure 8). These findings suggest another potential disease mechanism for the severe phenotype involving increased cell death due to mutant ANO4. This conclusion is supported by studies showing the role of apoptosis signaling in seizure-induced neuronal death and epileptogenesis^82^^,^^83^ and is in line with the phenotype of DEE, where stagnation or worsening correlating with increased seizure activity is observed.

Finally, we studied a potential dominant-negative effect of ANO4 variants on the wild type using a co-transfection protocol in HEK293 cells. We selected one DEE and one GEFS+ variant with a strong LoF (Asn558Lys and Val528Met) and observed that in the presence of wildtype ANO4 the mutant variants Val528Met and Asn558Lys still suppressed Ca^2+^-induced membrane conductance in a similar way as the mutants alone (Figure 9). Similarly, wild-type co-transfection did not alleviate the mutant effects on scramblase activity (as measured by the annexin A5 expression assays) but restored subcellular localization (Figures 9, S3 and S4). Moreover, a reduction in basal membrane conductance for these two variants was only observed when co-transfecting wild-type and mutant, but not for mutants alone (Table 5). In summary, the co-transfection experiments suggest a strong dominant-negative effect of the mutants on wild-type ANO4.

Structural analysis revealed that all seven variants identified in the present study are localized within the globular domain of ANO4 with predicted destabilization of its structure (Figure 2). Concerning preliminary genotype-phenotype correlation, we noted that the two inherited variants associated with the less severe phenotypes of GEFS+ and TLE are the only variants that do not introduce new large side chains or changes in charge. Moreover, these are the only variants that did not show reduced plasma membrane location. Notably, both Val528Met and Ile725Thr are located in TMs that are not in proximity to the Ca^2+^ binding site (TM3 and TM7, respectively). In contrast, all three variants occurring *de novo* in the most severe DEE phenotypes (Asn558Lys, Ile562Phe, and Met563Lys) cluster within transmembrane domain 4 close to the Ca^2+^ binding site indicating that they not only disrupt the ANO4 structure but also likely impact Ca^2+^ binding. The three DEE variants are also the only ones increasing ANO4 scramblase activity indicating increased apoptosis. Of the two *de novo* variants observed in EE, Asn603Asp detected in the child with the earlier onset of regression and more severe cognitive impairment is also located close to the Ca^2+^ binding site but in a different TM (TM5, Figure 2A). Finally, the Asn129Lys variant observed in the individual with childhood-onset regression and moderate ID at teenage age is the only variant located in the N-terminal intracellular region within the predicted dimerization domain (PF16178).^67^ In further support of this suggested genotype-phenotype correlation, four population variants with deleterious *in silico* predictions G115A, A535T, Y707C, and A728T, (dbSNP: rs34162417, rs150353677, rs143089752, and rs200450110, respectively)^84^ are either rare and located in extracellular loops (A535T, Y707C, A728T) or very frequent (G115A, MAF 0.0476 with 390 homozygotes in gnomAD v2.1.1) and located in the N-terminal intracellular region but, in contrast to the EE-associated Asn129Lys variant, outside the dimerization domain and without introduction of a charged side chain (Figure 2A).

In conclusion, this study identified missense variants in *ANO4* as a cause of fever-sensitive DEE/EE, GEFS+, and TLE and suggests possible pathophysiological mechanisms. The data suggest that the disease-associated variants act in a dominant-negative manner and lead to a decreased Ca^2+^-dependent cation conductance due to reduced surface expression and impaired cation channel function of ANO4. Increased apoptosis rates observed for three ANO4 mutants may contribute to the disease mechanism. Additional studies are needed to further explore and support the pathomechanisms and genotype-phenotype correlation proposed here and unravel essential information about the localization of ANO4 and its functional role in neurons.

## Data and code availability

The accession numbers for the *ANO4* variants reported in this study are ClinVar: SCV004809186, SCV004809187, SCV004809188, SCV004809189, SCV004809190, SCV004809191, and SCV004812191. There are restrictions to the availability of further genomic data of the individuals studied here due to the consent given by them or their legal guardians. All sharable genomic data are published in the supplement. Other data will be provided upon legitimate requests.

## Web resources

AlphaFold Protein Structure Database, alphafold.ebi.ac.uk/entry/Q32M45
Biorender online software, biorender.com
Genome Aggregation Database (gnomAD), gnomad.broadinstitute.org
Human Brain Transcriptome database, hbatlas.org
Mouse Genome Database (MGD), informatics.jax.org
Protein Data Bank (PDB), rcsb.org/structure/6qp6
The Human Protein Atlas, proteinatlas.org/ENSG00000151572-ANO4

## Supplemental information

Document S1. Supplemental note and Figures S1–S6

Table S1. Rare variants with MAF < 0.001 detected in the study cohort

Document S2. Article plus supplemental information
