# Supplementary material for: Missense variants in ANO4 cause sporadic encephalopathic or familial epilepsy with evidence for a dominant-negative effect
Source: Am J Hum Genet. 2024 May 13;111(6):1184–205. doi: 10.1016/j.ajhg.2024.04.014 (PMC11179416; doi:10.1016/j.ajhg.2024.04.014)
Supplement: Document S1. Supplemental note and Figures S1–S6 [file mmc1.pdf]

The American Journal of Human Genetics, Volume *111*

Supplemental information

Missense variants in *ANO4* cause sporadic encephalopathic or familial epilepsy with evidence for a dominant-negative effect

Fang Yang, Anais Begemann, Nadine Reichhart, Akvile Haeckel, Katharina Steindl, Eyk Schellenberger, Ronja Fini Sturm, Magalie Barth, Sissy Bassani, Paranchai Boonsawat, Thomas Courtin, Bruno Delobel, EuroEPINOMICS-RES Dravet working group, Boudewijn Gunning, Katia Hardies, Mélanie Jennesson, Louis Legoff, Tarja Linnankivi, Clément Prouteau, Noor Smal, Marta Spodenkiewicz, Sandra P. Toelle, Koen Van Gassen, Wim Van Paesschen, Nienke Verbeek, Alban Ziegler, Markus Zweier, Anselm H.C. Horn, Heinrich Sticht, Holger Lerche, Sarah Weckhuysen, Olaf Strauß, and Anita Rauch

Supplemental Note: Case Reports

Individual 1

Individual 1 was born to healthy non-consanguinous parents after an uneventful pregnancy. He was a floppy child and developed epilepsy at age 2m. The initial seizure type was multifocal clonic with cloni of eyebrows, arms, tongue and unresponsiveness. Initial EEG showed rare sharp waves median frontal and central region as well in the right centroparietal region.

During disease course, observed seizure semiology was an initial cry, stiff legs then cloni of arms and cyanosis, occurring often during sleep, about 1x/week. Another documented seizure type was shorter hypermotoric seizures with multifocal cloni occurring about 1-2x/week, sometimes in clusters. Multiple EEG's at follow ups showed multifocal sharp waves, polyspike waves, and abnormal background activity. Seizures were fever sensitive. Until the age of 4-5 years, he had a strong susceptibility to infections, which improved afterwards.

Individual 1 was first referred for genetic evaluation at age 12 months due to severe muscular hypotonia, developmental delay and epilepsy, but no diagnosis could be established. We saw the child again at the age of 2y 11m. Growth parameters were weight 12.8 kg (P50-75), length 100 cm (P90) and head circumference 48.8 cm (P3-10). He showed pectus excavatum and finger pads as minor morphologic features. He was able to grip better and turn from the supine to the prone position on his own, but continued to have marked hypotonia. He was not able to sit or eat independently. He did not speak and pointed to things he wanted. He had at that moment a seizure-free period on antiseizure treatment with Levetiracetam. EEG did not show epileptic discharges. Sequencing of *POLG* and chromosomal microarray analysis and metabolic workup were unremarkable. He was included in a study of genetic causes of epileptic encephalopathy and a *de novo* missense variant in *ANO4* NM_001286615.1:c.1688T>A p.(Met563Lys) was detected. Additionally, he had an incidental finding of compound-heterozygous variants in *GJB2* NM_004004.5:c.[35del];[101T>C] potentially associated with late-onset hearing loss.

At last investigation at age 12 2/12 years, he showed a content demeanour. Head circumference was 52.1 cm (P3-10, z -1.75). He could neither move nor speak. He had a hyperkinetic movement disorder with ataxia and myoclonia, a pectus excavatum, and a mild flexion contracture of the right knee. Some

but not all of the myoclonia had a correlation in the EEG. He had several EEG's without any epileptic activity despite observed pathologic movements of the limbs. Myoclonia were therefore interpreted as being due to a movement disorder with myoclonia rather than myoclonic epilepsy. The parents reported that he had generalized tonic-clonic seizures with head rotation to the side, cyanosis, drooling and then cloni of the arms, occurring on average 2 times a week, and more frequently during teething and infections. Furthermore, he had reflux with occasional vomiting. While selective eating habits were present in early childhood, he now ate with appetite and ate almost everything in small pieces. He received a laxative because of mild constipation. He had hippotherapy which led to significant improvement of his truncal muscle tone, so that he could now be transferred to the toilet chair. He had hypermetropia and a strabism corrected with glasses. His hearing was not tested formally but was good in daily life, e.g. he responded to whispering. He had a pronounced need for sleep with a night sleep of about 14 hours, and difficulties to wake up in the morning. A detailed endocrinological examination was unremarkable except for central hypothyroidism.

Individual 2

Individual 2 was the second boy from non-consanguineous parents and was born at 41 gestational weeks by primary C-section after an uneventful pregnancy. Growth parameters at birth were: weight 3310g, length 49.5 cm and OFC 36cm. APGAR score 10/10/10. In the neonatal period, axial hypotonia and peripheral hypertonia, sporadic rolling movements of the limbs, lack of eye contact, and feeding difficulties were noted. Seizure onset was at age 1 month. Brain MRI at that time was normal. Reported seizure types included generalized tonic clonic and focal impaired awareness. Seizures were therapy resistant typically occuring multiple times per week, and repeatedly excacerbated during infections. Antiepileptic treatments employed over the years included Phenobarbital, Valproate, Carbamazepine, Levetiracetam, Clonazepam, Phenytoin, and Topiramate. Ketogenic diet was given from age 1y 6m until 4y 4m when considered uneffective. At age 9y the child was reported to be seizure free during 1.5 years. At last follow up, antiepileptic treatment consisted of Levetiracetam, Clobazam, and Valproate.

Development was severely delayed from birth. Eye contact and babbling was noted at age 1y 6m. The child never acquired sitting and remained non-verbal.

Brain MRI at age 1y 2m showed severely delayed myelinisation, and follow up at age 2y 7m showed a cortico-subcortical atrophy with some white matter anomalies and cerebellar atrophy. The child suffered from severe spasticity and dystonia, treated with Hydrocortisone, Baclofen, Levodopa / Benserazide, and Clonidine. Associated pain was treated with Morphine, 5-Hydroxytryptophan and Amitriptyline. At age 6.5 months chronic vomiting and feeding difficulties led to gastrostomy. Vomiting episodes continued and could not be controlled by various medications. At age 1y 2m, failure to thrive was still a problem. Exacerbation of spasms and dystonia were reported during vomiting episodes.

At age 3y, optic atrophy and severe kyphosis were first reported. The child developed very severe scoliosis at the T5-T11 levels resulting in ventilation troubles i.e. a hypercapnia requiring oxygen therapy. Sacrum angulation S3-S4 is at 90°. At the pelvic level, the left hip is dislocated with a flexion of 60° and the right hip flexion is completely abolished. Furthermore, the child has bilateral knee contractures and feet deformation. At age 9y diffuse osteoporosis was diagnosed after a pathological femoral shaft fracture.

Trio exome sequencing analysis showed a *de novo* heterozygous NM_001286615.1:c.1674C>A p.(Asn558Lys) variant in *ANO4*.

The child deceased at age 12y 6m due to neurological and respiratory decompensation.

Individual 4

This 22-year-old male is the second child of healthy non consanguineous parents. He was born at term and his early developmental milestones were normal.

At the age of 11 months he had the first cluster of tonic/tonic- clonic seizures during a febrile infection. Afebrile seizures followed and focal impaired awareness seizures and myoclonias were observed from age 16 months. Seizures were triggered by fever, heat, infection and later by physical exercise. His psychomotor development was normal until 1,5 years of age, then slowed and stagnated during the third year of life, speech production being the most affected area.

EEG was normal at seizure onset. From 1,5 years of age, slowing of background activity and scarse interictal epileptiform findings, including frontocentral spikes independently form both hemispheres and generalized spike- and polyspike-and-wave discharges during sleep, were observed. At 3 and 9 years of

age, several seizures were caught in a video-EEG; 1. Focal impaired awareness seizures, evolving to right or left sided motor seizures. 2. sequential seizures starting with impaired awareness, followed by right-sided tonic-clonic and then generalized tonic-clonic and finally left sided motor seizure. EEG discharge was first non- lateralizing —then evolved to left hemisphere — generalized and finally shifted to right hemisphere. Myoclonia were associated with generalized polyspike-and-wave discharges and arousals from sleep with right centroparietal discharges. A diagnosis of Dravet-like syndrome was given, but no pathogenic variant of *SCN1A* was found.

During childhood, his seizure frequency varied from multiple per week -with clustering- to seizure freedom up to one year despite multiple anti- seizures medications (Table 2). Sodium channel blockers were not effective and were associated with gait instability. He has been seizure- free since age 17 years, when bromide was added to valproate, topiramate and clobazam. Presently, he continues with valproate and topiramate.

His psychomotor development was normal until 1,5 years of age, then slowed and stagnated during the third year of life. Speak production even regressed and he used signs and gestures to communicate. He learned to run and jump but had muscular hypotonia and balance problems. In a neuropsychological examination at the age of 5 years he performed at the level of mild intellectual disability and by 12 years of age he had declined cognitively to the level of moderate intellectual disability. According to parents, his skills have further regressed during early adulthood years; speech production is only a few words, speech reception is impaired, and he is uncapable of assembling puzzles, which previously used to be the best skill level.

Trio exome sequencing analysis showed a *de novo* heterozygous NM_001286615.1:c.1807A>G p.(Asn603Asp) variant in *ANO4*.

Individual 5

This 20-year-old male is the first child of healthy non-consanguineous parents. He was born at 36+3 gestational weeks. His early development was normal. At the age of 3,5 years, he had a febrile seizure. Soon other non-fever sensitive seizure types occurred, including tonic-clonic seizures (with afterwards shaking of his left hand), tonic seizures, focal seizures with impaired awareness and twitching in his

face, and myoclonia. He has frequent nocturnal seizures. His epilepsy was therapy resistant and classified as probably (multi)focal.

Antiepileptic treatment initially consisted of Valproate, Rufinamide and Clobazam. Ketogenic diet was introduced at age 5y and initially had a beneficial effect on the seizures, until about age 7y when it was no longer effective. At age 8y he received Valproate, Phenytoin, Lacosamide, and Clobazam. He started with vagal nerve stimulation which was effective to stop generalized tonic clonic seizures. After stopping Phenytoin his locomotion improved. Levetiracetam, Topiramate, and Zonisamide had had little effect on his seizures. At age 11y he received ketogenic diet again, making him more alert but without effect on the seizures. At age 16y Rufinamide was added, improving the generalized tonic clonic seizures, so that Lacosamide could be stopped. At last follow up at age 23y he was treated with Valproate, Rufinamide and Clobazam in addition to the vagus nerve stimulation.

MRI scans of his brain showed stable periventricular white matter anomalies. An MRI scan of the brain at age 16 years showed progressive cerebral atrophy.

Until age 4 years his cognitive development was normal. At age 4y 9m he scored an IQ of 100 in formal assessment. At age 7y he no longer benefited from ketogenic diet, and with many more seizures there was a cognitive decline at the age of 8y 1m (verbal IQ of 78 and performance IQ of 72 on WISC-III). At age 8y 6m he was diagnosed with epileptic encephalopathy. At age 14y he was diagnosed with autism spectrum disorder. At age 14 and 16 years his IQ score was 51.

He had low energy level. He used CPAP during the night due to sleep apnea. He had a normal height (186 cm, +0.4 SD) with a relatively large arm span (192 cm; ratio to height 1,03) and a relatively large head circumference (60.5 cm, +1.9 SD). He had (familial) hypertelorism and showed striae around the lower back and armpits.

Genetic analysis showed a *de novo* heterozygous NM_001286615.1:c.387C>G p.(Asn129Lys) variant in *ANO4*.

Individual 7

Individual 7 is a 39-year-old female from a non-consanguineous family. She was known to have a coarcatio aorta and was followed since the age of 8 years at the child neurology department because of

gait difficulties. These were caused by a dopa responsive dystonia due to a heterozygous deletion of the GTP cyclohydrolase I gene, and were controlled with a low dose of levodopa. Neurodevelopment was further normal. From the age of 18 years, she had recurrent short episodes that started with a déjà-vu or intrusive thoughts, followed by staring. Afterwards there was mild confusion, tiredness, and headache. By the age of 23 years, these episodes occurred up to 6 times a month. MRI of the brain showed an unspecific white matter lesion in the right centrum semiovale. EEG and 24-hours EEG were normal. A clinical diagnosis of temporal lobe epilepsy was made, and treatment with carbamazepine was started, after which the episodes disappeared. Carbamazepine was switched to levetiracetam since she planned a pregnancy. She discontinued levetiracetam at the age of 27 and has remained seizure free since. The brother of her maternal grandmother had died at the age of 16 years of severe epilepsy.

Individual 7 had an inconspicuous epilepsy panel analysis and was included in a cohort of individuals with febrile seizures and/or temporal lobe epilepsy for a *ANO4* candidate gene screening, which revealed the rare missense variant NM_001286615.1: c.2174T>C p.(Ile725Thr). The variant was inherited from her asymptomatic mother. DNA of other family members was not available for further segregation analysis.

Supplemental Figures

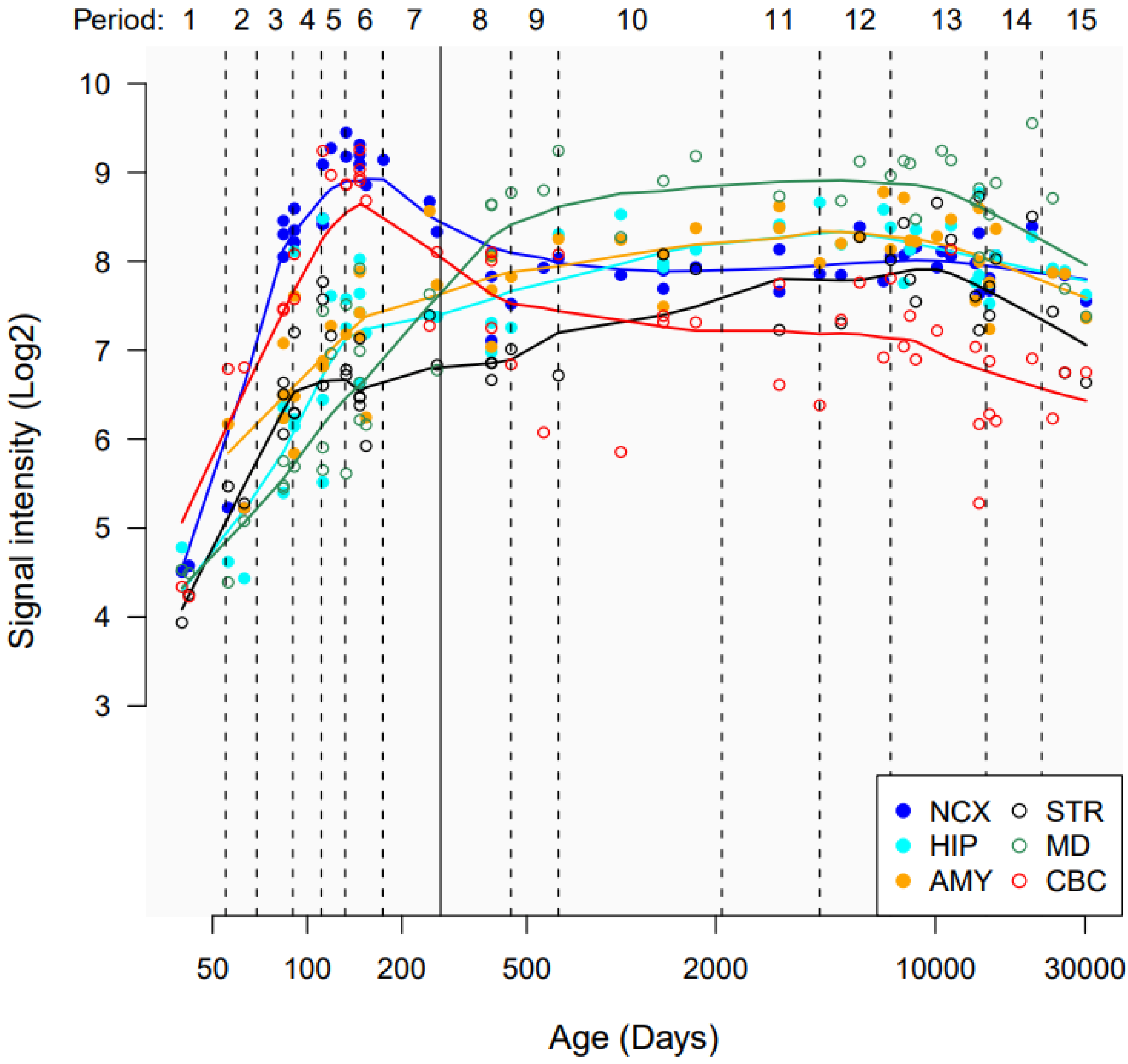

Figure S1. *ANO4* mRNA expression in humans retrieved from the human brain transcriptome database. Levels of human *ANO4* mRNA are depicted in different brain regions during pre- and postnatal periods.

Brain regions are abbreviated as follows: NCX = neocortex, HIP = Hippocampus, AMY = Amygdala, STR = Striatum, MD = Mediodorsal nucleus of the thalamus, CBC = Cerebellar cortex. On the top X axis, periods 1-7 denote embryonic and fetal development, specifically period 1 from post-conceptional weeks (PCW) 4 to 8, period 2 from PCW 8 to 10, period 3 from PCW 10 to 13, period 4 from PCW 13 to 16, period 5 from PCW 16 to 19, period 6 from PCW 19 to 24, and period 7 from PCW 24 to 38. Period 8 denotes neonatal and early infancy until age 6 months, period 9 refers to late infancy from age

6 months to 12 months, period 10 refers to early childhood from age 1 to 6 years, period 11 represents middle and late childhood from age 6 to 12 years, period 12 denotes adolescence from age 12 to 20 years, period 13 refers to young adulthood from age 20 to 40 years, period 14 represents middle adulthood from age 40 to 60 years, and period 15 denotes late adulthood from age 60 years onwards. Figure retrieved from the Human Brain Transcriptome database[1] at hbatlas.org on January 17 2024.

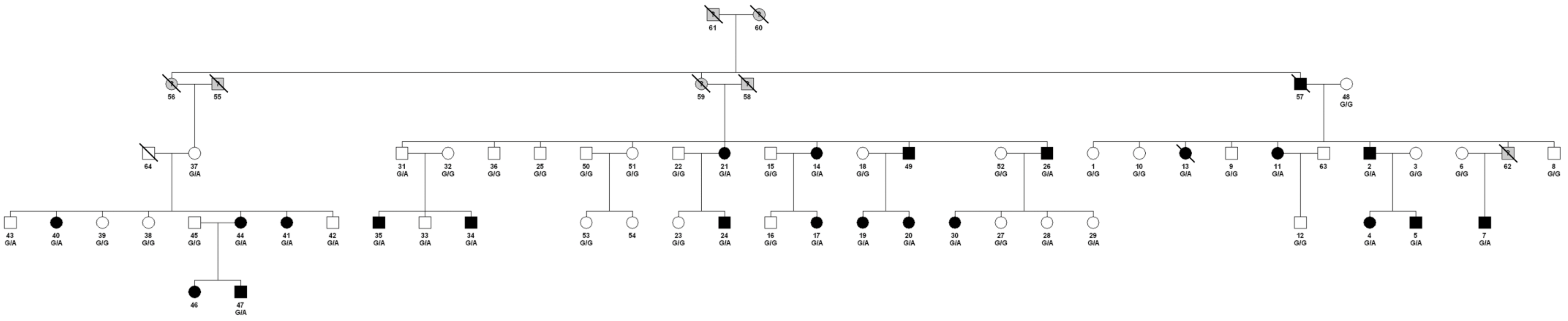

Figure S2. Pedigree of Family 6. Pedigree of members of family 6 that were available for linkage analysis and subsequent *ANO4* variant testing showing 23 individuals affected by GEFS+ (black filled symbols) with an autosomal-dominant inheritance pattern. Clinical information on the extemded family was previously published in Depondt et al. 2002[2]. Grey filled symbols with question mark denote unknown phenotype. If DNA was available for analysis the genotype is given below the individual (G/G wildtype, G/A heterozygous for the *ANO4* variant NM_001286615.1:c.1582G>A). The identified segregating *ANO4* variant was present in all 20 affected family members for whom DNA was available and had an 73% penetrance (22 affected [obligate] carriers/30 total [obligate] carriers).

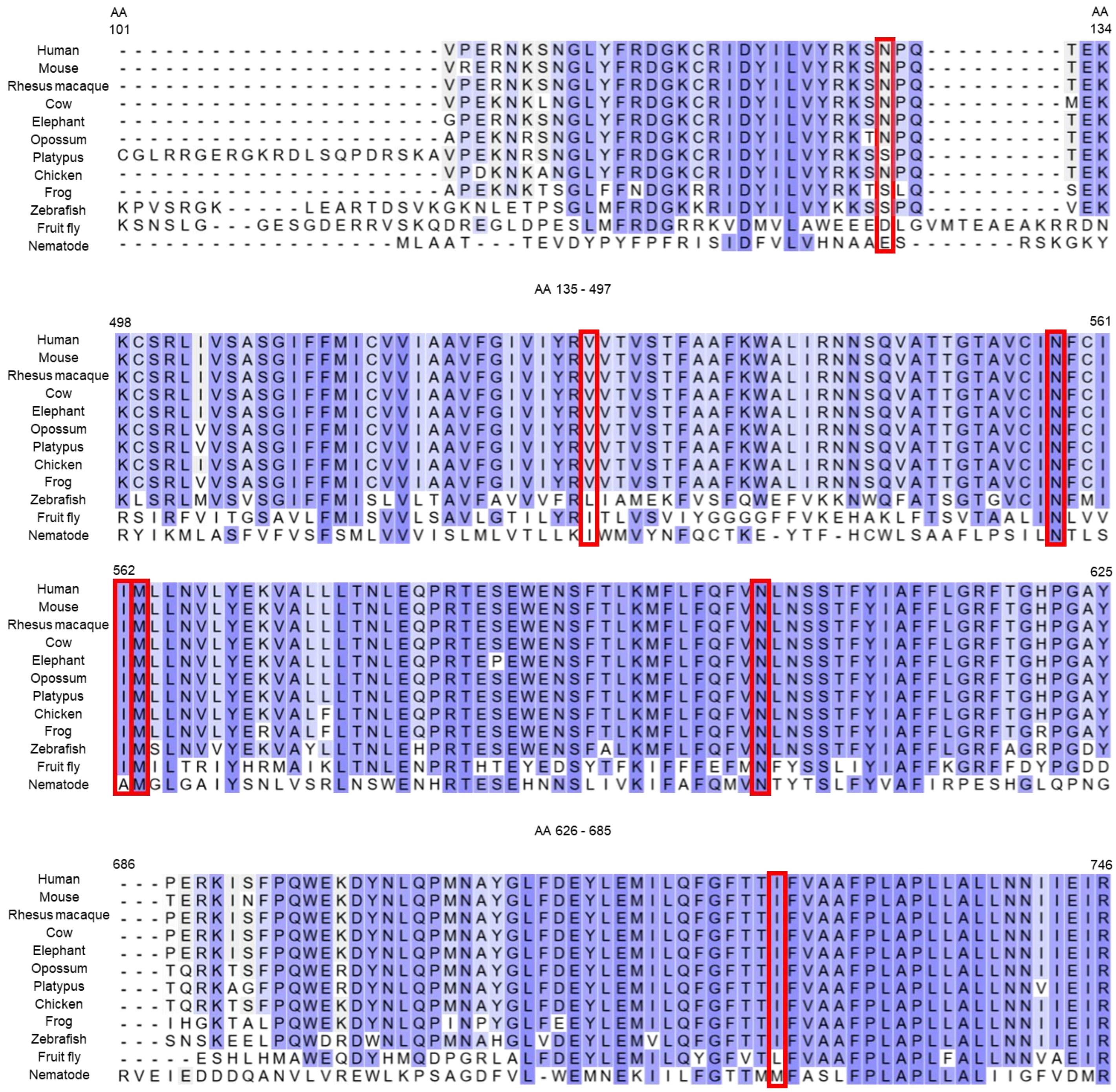

Figure S3. Multiple sequence alignment of human *ANO4* with orthologues. Multiple sequence alignment performed with Clustal Omega[3] between human *ANO4* and orthologues indicates sequence homology with mouse *Ano4* and evolutionary conservation at sites of the seven disease-associated variants investigated in this study highlighted in red.

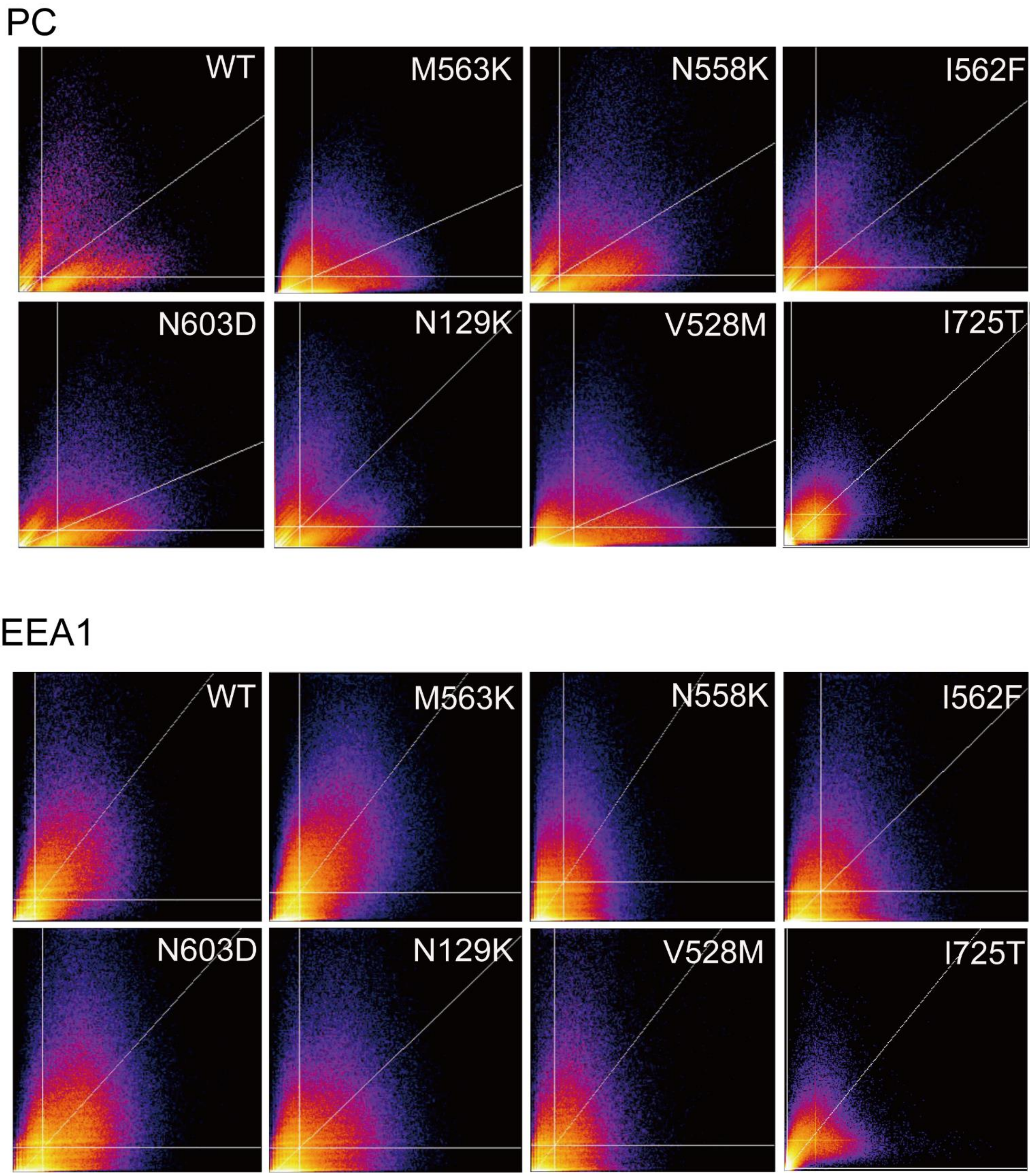

Figure S4. Examples of pixel color spectra used for the calculation of Pearson's co-localization coefficient. Pixel spectra for the co-localization analysis of ANO4 wildtype or mutants Met563Lys (M563K), Asn558Lys (N558K), Ile562Phe (I562F), Asn603Asp (N603D), Asn129Lys (N129K), Val528Met (V528M), and Ile725Thr (I725T) with pan-cadherin (PC) (upper panel) or the early endosome marker EEA1 (lower panel).

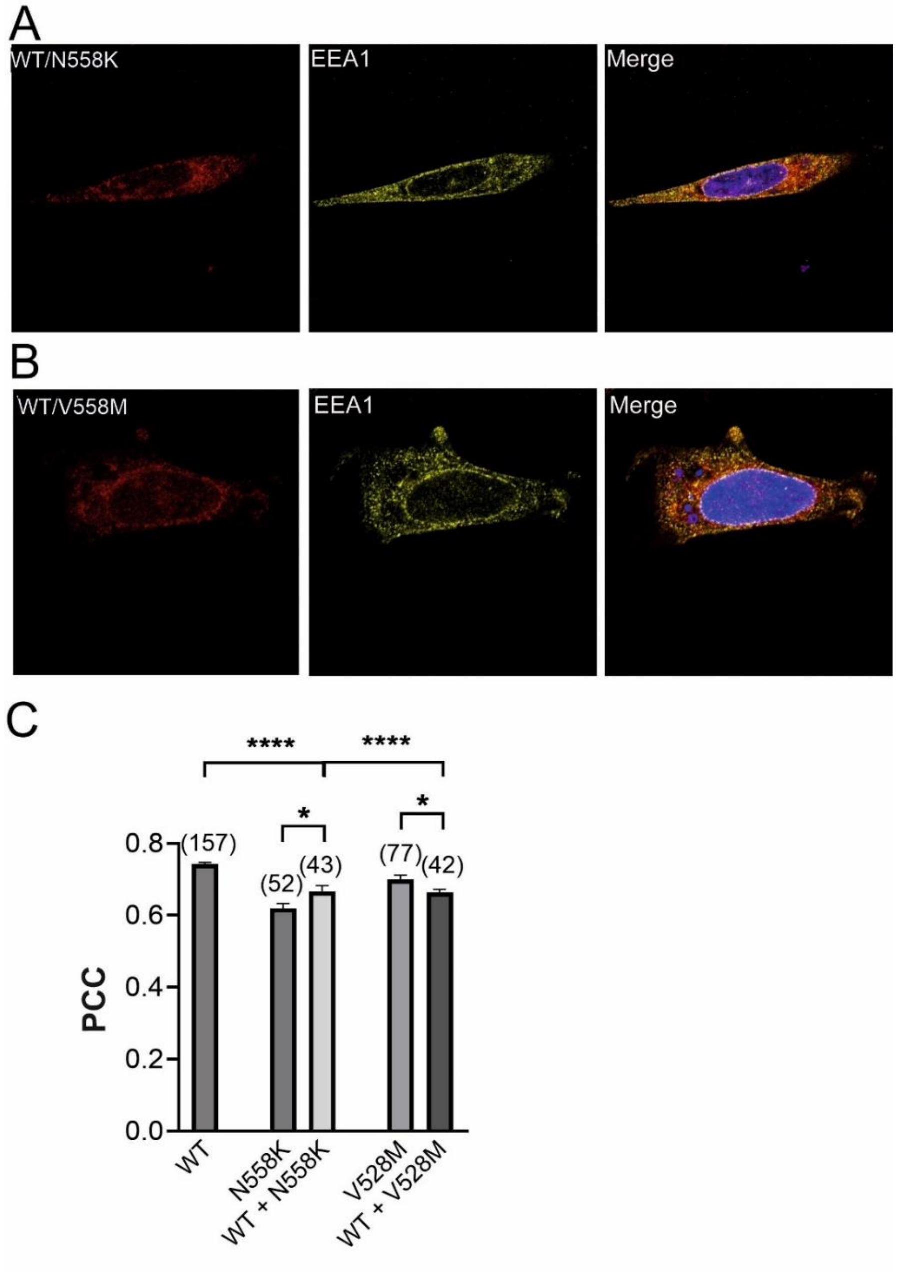

Figure S5. Co-localization of ANO4 wildtype/Asn558Lys or wildtype/Val528Met with the early endosome marker EEA1 in HEK293 cells. (A, B) HEK293 cells co-expressing wildtype and either Asn558Lys (N558K; A) or Val528Met (V528M; B) mutated *ANO4* constructs were stained with antibodies for ANO4 (left panel, red) and EEA1 (middle panel, yellow). Merged images (right panel) show the co-localization of ANO4 and EEA1. Nuclei were counterstained with DAPI (blue). (C) Pearson's correlation coefficient (PCC) analysis of EEA1 and mutant ANO4 (transfection and immunostaining according to (A-H). The number inside each bar represents n per group. Values are given as mean ± SEM. Multiple comparisons were performed by ANOVA with Tuckey's post hoc test. *p < 0.05; **p < 0.01; ***p < 0.001.

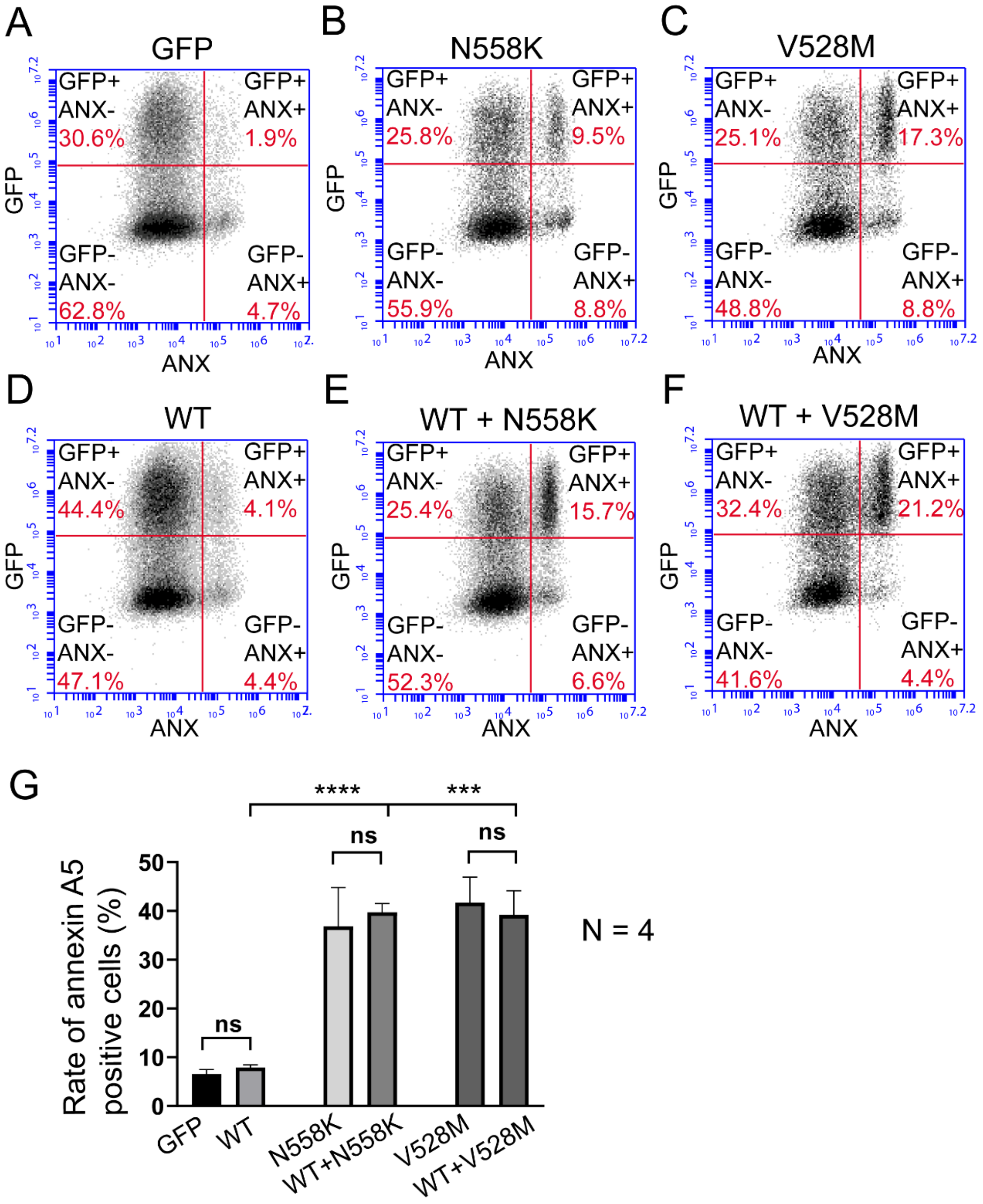

Figure S6. Scramblase activity in HEK293 cells co-expressing wildtype and mutant ANO4 at physiological Ca^{2+} levels. Scramblase activity was assessed by FACS sorting of Annexin A5-labeled HEK293 cells that were not treated by ionomycin and transfected with GFP alone (A), ANO4 mutant Asn558Lys (N558K) plus GFP (B), ANO4 mutant Val528Met (V528M) plus GFP (C), wildtype ANO4 plus GFP (D), ANO4 wildtype and Asn558Lys (N558K) mutant plus GFP (E), or ANO4 wildtype and Val528Met (V528M) mutant plus GFP (F). X-axis: Fluorescence intensity of Anx A5-6S-IDCC (log); Y-axis: Fluorescence intensity of GFP (log). The right upper square represents the ANO4 transfected, Annexin A5-positive cell fraction. (G) Comparison of annexin A5 surface

expression between different transfection conditions. The experiments were carried out four times (N = 4). Values are given as mean ± SEM. Multiple comparisons were performed by ANOVA with Tuckey's post hoc test. *p < 0.05; **p < 0.01; ***p < 0.001.

Supplemental References

1. Kang, H.J., Kawasawa, Y.I., Cheng, F., Zhu, Y., Xu, X., Li, M., Sousa, A.M.M., Pletikos, M., Meyer, K.A., Sedmak, G., et al. (2011). Spatio-temporal transcriptome of the human brain. Nature *478*, 483-489. 10.1038/nature10523.
2. Depondt, C., Van Paesschen, W., Matthijs, G., Legius, E., Martens, K., Demaerel, P., and Wilms, G. (2002). Familial temporal lobe epilepsy with febrile seizures. Neurology *58*, 1429-1433. 10.1212/wnl.58.9.1429.
3. Sievers, F., Wilm, A., Dineen, D., Gibson, T.J., Karplus, K., Li, W., Lopez, R., McWilliam, H., Remmert, M., Soding, J., et al. (2011). Fast, scalable generation of high-quality protein multiple sequence alignments using Clustal Omega. Mol Syst Biol *7*, 539. 10.1038/msb.2011.75.
