# Supplementary material for: Missense variants in ANO4 cause sporadic encephalopathic or familial epilepsy with evidence for a dominant-negative effect
Source: Am J Hum Genet. 2024 May 13;111(6):1184–205. doi: 10.1016/j.ajhg.2024.04.014 (PMC11179416; doi:10.1016/j.ajhg.2024.04.014)
Supplement: Document S2. Article plus supplemental information [file mmc3.pdf]

# Missense variants in *ANO4* cause sporadic encephalopathic or familial epilepsy with evidence for a dominant-negative effect


## Authors

Fang Yang, Anais Begemann,
Nadine Reichhart, ..., Sarah Weckhuysen,
Olaf Strauß, Anita Rauch

## Correspondence

anita.rauch@medgen.uzh.ch



**This paper establishes missense variants in the anoctamin family member *ANO4* (or *TMEM16D*) as a cause of distinct epileptic disorders. Resulting phenotypes depend on the location in the protein and the functional impact of the substitution such as loss of cation channel function and increased apoptosis signaling.**



Check for updates

Yang et al., 2024, The American Journal of Human Genetics *111*, 1184–1205

June 6, 2024 © 2024 The Author(s).

https://doi.org/10.1016/j.ajhg.2024.04.014

CellPress

ARTICLE

# Missense variants in *ANO4* cause sporadic encephalopathic or familial epilepsy with evidence for a dominant-negative effect

Fang Yang,[1,22] Anais Begemann,[2,22] Nadine Reichhart,[1] Akvile Haeckel,[3] Katharina Steindl,[2] Eyk Schellenberger,[3] Ronja Fini Sturm,[1] Magalie Barth,[4] Sissy Bassani,[2] Paranchai Boonsawat,[2] Thomas Courtin,[5,6] Bruno Delobel,[7] EuroEPINOMICS-RES Dravet working group, Boudewijn Gunning,[8] Katia Hardies,[9] Mélanie Jennesson,[10] Louis Legoff,[4] Tarja Linnankivi,[11,12] Clément Prouteau,[4] Noor Smal,[9] Marta Spodenkiewicz,[13] Sandra P. Toelle,[14] Koen Van Gassen,[15] Wim Van Paesschen,[16] Nienke Verbeek,[15] Alban Ziegler,[4] Markus Zweier,[2] Anselm H.C. Horn,[2,17] Heinrich Sticht,[17] Holger Lerche,[18] Sarah Weckhuysen,[9,19,20] Olaf Strauß,[1] and Anita Rauch[2,21,*]

**Summary**

Anoctamins are a family of $Ca^{2+}$-activated proteins that may act as ion channels and/or phospholipid scramblases with limited understanding of function and disease association. Here, we identified five *de novo* and two inherited missense variants in *ANO4* (alias *TMEM16D*) as a cause of fever-sensitive developmental and epileptic or epileptic encephalopathy (DEE/EE) and generalized epilepsy with febrile seizures plus (GEFS+) or temporal lobe epilepsy. *In silico* modeling of the ANO4 structure predicted that all identified variants lead to destabilization of the ANO4 structure. Four variants are localized close to the $Ca^{2+}$ binding sites of ANO4, suggesting impaired protein function. Variant mapping to the protein topology suggests a preliminary genotype-phenotype correlation. Moreover, the observation of a heterozygous *ANO4* deletion in a healthy individual suggests a dysfunctional protein as disease mechanism rather than haploinsufficiency. To test this hypothesis, we examined mutant ANO4 functional properties in a heterologous expression system by patch-clamp recordings, immunocytochemistry, and surface expression of annexin A5 as a measure of phosphatidylserine scramblase activity. All *ANO4* variants showed severe loss of ion channel function and DEE/EE associated variants presented mild loss of surface expression due to impaired plasma membrane trafficking. Increased levels of $Ca^{2+}$-independent annexin A5 at the cell surface suggested an increased apoptosis rate in DEE-mutant expressing cells, but no changes in $Ca^{2+}$-dependent scramblase activity were observed. Co-transfection with ANO4 wild-type suggested a dominant-negative effect. In summary, we expand the genetic base for both encephalopathic sporadic and inherited fever-sensitive epilepsies and link germline variants in *ANO4* to a hereditary disease.

## Introduction

During the last decade, the important contribution of Mendelian disorders to the etiology of epilepsies became increasingly evident. According to a current meta-analysis, overall diagnostic yields are 24% for exome sequencing and 48% for genome sequencing[1] but are highly variable depending on cohort selection and date of the study. Moreover, since exome sequencing has improved over time, the current diagnostic yield of exome sequencing is expected to be closer to genome sequencing as pointed out by Grether et al.[2] The diagnostic yield of genetic testing is highest in early-onset developmental and epileptic or epileptic encephalopathy (DEE/EE), which are severe disorders combining early-onset epilepsy and neurodevelopmental delay, but still about half of the cases remain without diagnosis.[3,4] Undiagnosed cases may be explained by variants that are difficult to detect, variants of uncertain significance, or novel disease-associated genes. Here, we present evidence that variants in *ANO4* (anoctamin 4 or

[1]Experimental Ophthalmology, Department of Ophthalmology, Charité – Universitätsmedizin Berlin, a Corporate Member of Freie Universität, Humboldt-University, the Berlin Institute of Health, Berlin, Germany; [2]Institute of Medical Genetics, University of Zurich, Schlieren-Zurich, Switzerland; [3]Institute for Radiology and Children's Radiology, Charité–Universitätsmedizin Berlin, a Corporate Member of Freie Universität, Humboldt-University, the Berlin Institute of Health, Berlin, Germany; [4]University Hospital of Angers, Department of Genetics, Angers, France; [5]Sorbonne Université, INSERM, CNRS, Institut du Cerveau - Paris Brain Institute - ICM, 75013 Paris, France; [6]Hôpital Pitié-Salpêtrière, DMU BioGe'M, AP-HP, 75013 Paris, France; [7]Service de Cytogénétique, GH de l'Institut Catholique de Lille, Hopital Saint Vincent de Paul, Lille, France; [8]Stichting Epilepsie Instellingen Nederland, Zwolle, the Netherlands; [9]Applied & Translational Neurogenomics Group, VIB Center for Molecular Neurology, VIB, University of Antwerp, 2610 Antwerp, Belgium; [10]Department of Pediatrics, CHU, Reims, France; [11]Epilepsia Helsinki, University of Helsinki and Helsinki University Hospital, 00029 HUS Helsinki, Finland; [12]Department of Pediatric Neurology and Pediatric Research Center, New Children's Hospital, Helsinki University Hospital and University of Helsinki, 00029 HUS Helsinki, Finland; [13]Department of Genetics, La Réunion University Hospital, Saint-Pierre, France; [14]Department of Pediatric Neurology, Children's University Hospital Zurich, Zurich, Switzerland; [15]University Medical Center Utrecht, Department of Genetics, Utrecht, the Netherlands; [16]Laboratory for Epilepsy Research, KU Leuven, and Neurology Department, University Hospitals Leuven, 3000 Leuven, Belgium; [17]Division of Bioinformatics, Institute of Biochemistry, Friedrich-Alexander-Universität Erlangen-Nürnberg, Erlangen, Germany; [18]Department of Neurology and Epileptology, Hertie-Institute for Clinical Brain Research, University of Tübingen, Tübingen, Germany; [19]Department of Neurology, Antwerp University Hospital, Antwerp, Belgium; [20]Translational Neurosciences, Faculty of Medicine and Health Science, University of Antwerp, 2610 Antwerp, Belgium; [21]Children's University Hospital Zurich, Zurich, Switzerland
[22]These authors contributed equally
*Correspondence: anita.rauch@medgen.uzh.ch
https://doi.org/10.1016/j.ajhg.2024.04.014.
© 2024 The Author(s). This is an open access article under the CC BY license (http://creativecommons.org/licenses/by/4.0/).

*TMEM16D* [MIM: 610111]) cause sporadic encephalopathic and inherited epilepsy.

Anoctamins (ANOs) are highly conserved calcium-activated proteins. The ANO family encompasses ten members (ANO1 to ANO10, alias TMEM16A-K) that assemble as dimers[5] and function as $Ca^{2+}$-dependent ion channels and $Ca^{2+}$-dependent phospholipid scramblases.[5–11] Phospholipid scrambling refers to the translocation of phospholipids between the two leaflets of the lipid bilayer membrane, leading to loss of lipid asymmetry and to the externalization of phosphatidylserine (PS). Exposure of PS is essential for platelet activation and promotion of blood coagulation, suppression of inflammatory responses, and functions as a marker of apoptotic cells for recognition and clearance.[12] ANO1 (MIM: 610108), ANO2 (MIM: 610109), ANO4, ANO6 (MIM: 608663), and ANO7 (MIM: 605096) are predominantly located in the plasma membrane, while ANO8 (MIM: 610216), ANO9 (MIM: 619963), and ANO10 (MIM: 613726) are mostly expressed in the cytosol.[13,14] ANO1 and ANO2 have been shown to function as $Ca^{2+}$-dependent $Cl^{-}$ channels.[15–19] ANO3 (MIM: 610110), ANO4, ANO5 (MIM: 608662), ANO6, ANO7, and ANO9 were reported as phospholipid scramblases and/or ion channels and possibly regulate the activity of other endogenous ion channels due to their phospholipid scramblase activity.[8,9,11,20–24]

ANO proteins show multiple tissue-specific isoforms, which display distinct gating properties.[9,23,25–30] ANO proteins are involved in the control of neuronal cell excitability, olfaction, nociception, epithelial fluid secretion, contraction of smooth muscle, repair of the skeletal muscle membrane, and tumorigenesis. The broad spectrum of tissue expression explains the wide range of disorders associated with their respective disrupted function. Several ANO family members have been associated with a variety of human diseases or increased risks for diseases such as intestinal dysmotility (MIM: 620045)[31,32] and moyamoya disease (MIM: 620687)[33] with *ANO1*, dystonia (MIM: 615034)[34,35] with *ANO3*, gnathodiaphyseal dysplasia (MIM: 166260)[36–39] and muscular dystrophies (MIM: 613319, 611307)[40–44] with *ANO5*, disturbed blood coagulation (Scott syndrome, MIM 262890)[24,45] with *ANO6*, spinocerebellar ataxia (MIM: 613728)[46–48] with *ANO10*, and pancreatic and colorectal cancer[49,50] with *ANO9*. *ANO4* has not been linked to a Mendelian phenotype so far, but genome-wide association studies showed an association between single-nucleotide polymorphisms near *ANO4* and various neurological diseases, such as schizophrenia, Alzheimer disease, and anxiety disorder.[51–54]

Compared to well-studied members of the ANO family (ANO1, ANO2, and ANO6), the physiological functions and pathophysiology of ANO4 remain poorly investigated and have long been debated. ANO4 is mainly expressed in the central nervous system, particularly in inhibitory neurons and oligodendrocytes, and in endocrine and reproductive tissues.[55] During human embryonic development, *ANO4* mRNA is expressed in the human neocortex and cerebellar cortex with its peak in the mid-fetal development stages, then levels decrease during postnatal development and remain relatively stable in adulthood (Figure S1).[56] In contrast, hippocampus, amygdala, mediodorsal nucleus of the thalamus, and striatum display relatively low *ANO4* mRNA levels during prenatal development, with maximal detection during the postnatal periods.

Brown and colleagues indicated that ANO4 might participate in the regulation of aldosterone secretion and refuted its putative role as a $Ca^{2+}$-dependent chloride channel.[57,58] Furthermore, the levels of ANO4 expression were increased in active myelin lesions in multiple sclerosis.[59] A meta-analysis of genome-wide association studies in breast cancer identified variants around the ANO4 locus as risk factors.[60] Recent work more precisely described the function of ANO4 as being a $Ca^{2+}$-dependent cation channel involved in the regulation of $Ca^{2+}$ signaling in both plasma membranes and membranes of organelles when heterologously expressed in human embryonal kidney (HEK293) cells or when endogenously expressed in retinal pigment epithelial cells.[61] Potential scramblase activity of ANO4 remains uncertain.[21,61] More recent investigations revealed that currents facilitated by the ANO4 channel are required to activate glucose-inhibited neurons situated in the ventromedial hypothalamic nucleus (VMH) of mice in reaction to diminished glucose levels. Knockout of *Ano4* within the VMH exhibited a dual outcome: a decrease in blood glucose levels and a compromised ability to mount counterregulatory responses to hypoglycemic episodes.[62] A further mouse model carrying a homozygous intragenic deletion in *Ano4* presented with hyperactivity, reduced bone mineral content, decreased body weight, and altered cholesterol ratio compared to wild-type mice (Mouse Genome Database [MGD]; MGI: 2443344). Ano4 overexpression has been shown to attenuate calcium-mediated aldosterone secretion and reduce cell proliferation in a human adenocarcinoma cell line (H295R).[57] Furthermore, ANO4 was found upregulated in human tumor samples compared to normal kidney tissue, suggesting a potential role as a prognostic biomarker in non-metastasized clear cell renal cell carcinoma.[31]

In this paper, we describe seven *ANO4* missense variants identified in either sporadic individuals with DEE or EE or families with genetic epilepsy with febrile seizures plus (GEFS+) or temporal lobe epilepsy (TLE). We evaluated functional effects of the variants by overexpressing wild-type or mutant *ANO4* constructs in HEK293 cells. We measured the plasma membrane and subcellular localization by immunostaining and ion conductance using whole-cell patch clamping, as well as scramblase activity by binding of fluorescently labeled annexin A5 to exposed PS. We further employed structural modeling to gain insights into genotype-phenotype correlation.

## Material and methods

### Study cohort and genetic analyses

Through the GeneMatcher platform,[63] we recruited/identified five individuals (I1–I5) with epilepsy and intellectual disability (ID) harboring *de novo ANO4* missense variants identified by trio exome sequencing at the respective center as the only candidate causative variants. I1 was studied within the cohort published by Papuc et al.,[4] remaining without definite diagnosis. I4 was exome sequenced as part of the efforts of the EuroEPINOMICS-RES consortium. All detected rare variants (minor allele frequency [MAF] $<0.001$) with fitting inheritance pattern of I1, I2, I4, and I5 are provided in Table S1 (data from I3 not available). Family 6 (F6) consists of a previously published large pedigree with 23 family members affected by GEFS+ (formerly described as familial TLE with febrile seizures) with autosomal-dominant inheritance pattern (Figure S2).[64] By linkage analysis in this family, the disease gene locus was previously mapped to 12q22–q23.3, narrowing down the candidate region to a 10.35 cM (8.7 Mb) interval between D12S101 and D12S360 with the highest two-point LOD score of 6.94 and multipoint LOD score of 8.87 for marker D12S1706.[65] Whole-genome sequencing in two affected family members revealed a single candidate missense variant in *ANO4* that segregated within the family. Only one other rare variant was present in the linked locus in *PAH*, which is not a candidate variant for epilepsy since it is associated with the well-established autosomal-recessive disease phenylketonuria (Table S1). Following this finding, I7 with a phenotype of TLE was identified through candidate gene sequencing in a cohort of individuals with febrile seizures and/or TLE. All rare variants with MAF $<0.001$ detected by an exome-based epilepsy panel performed in I7 are provided in the Table S1. Individual ID: 272667 was recruited through the database of genomic variation and phenotype in humans using ensembl resources (DECIPHER).[66] The procedures followed were in accordance with the ethical standards of the respective responsible committees on human experimentation, and proper informed consent was obtained. *ANO4* variant nomenclature is given according to reference transcript GenBank: NM_001286615.1 (corresponding to ENST00000392977.3) and reference protein GenBank: NP_001273544.1 throughout the manuscript.

### Structural analysis of ANO4 variants

The membrane topology model of ANO4 (GRCh37 [GenBank: NM_001286615.1]) in Figure 2A was created based on sequence alignment to the experimentally observed topology of the ortholog in *N. haematococca*.[5] The indicated dimerization domain was determined by alignment to the published dimerization domain in mouse Ano1.[67] The figure was drawn with BioRender online software.

The structural analysis of ANO4 was based on a model available in the AlphaFold Protein Structure Database (Q32M45).[68,69] The subunit orientation and location of ion binding sites was adopted from the experimental structure of murine Ano6 (Protein Data Bank [PDB]: 6QP6).[70] VMD[71] was used for structure analysis and visualization.

### Cell culture and transfection

All experiments were performed in HEK293 cells (CRL-1573; ATCC, Wesel, Germany) with low passage numbers. These cells are a standard model to test ion channel variants, and as shown in our previous work, they do not show endogenous ANO4-induced currents.[61] HEK293 cells were grown in 25-cm$^2$ tissue culture flasks in Dulbecco's Modified Eagle's Medium (Gibco, Karlsruhe, Germany) supplemented with 10% (v/v) fetal bovine serum (Biochrom, Berlin, Germany) and 1% (v/v) penicillin-streptomycin (Biochrom, Berlin, Germany). Cells were maintained at 37°C and 5% CO2 in an incubator with 95% relative humidity. For functional assays, HEK293 cells were seeded on 12-well plates with sterilized 15-mm glass cover slips previously coated with 0.01% (w/v) Poly-L-lysine (Sigma-Aldrich, Co, St. Louis, USA) and transfected with the *ANO4* plasmids the following day using Lipofectamine 2000 Transfection Reagent (Invitrogen, Darmstadt, Germany), according to the manufacturer's protocol. For transfection, we mixed 0.2 μg plasmid DNA with 0.4 μL Lipofectamine. In the case of wild-type and mutant combined transfection, 0.2 μg contained 50% wild-type and 50% mutant Ano4 plasmid.

### Site-directed mutagenesis of *ANO4*

The full-length coding sequence of mouse *Ano4* was acquired with the previously described method.[61] Sequence alignments of *ANO4* were performed using Clustal Omega.[72] Locations of human disease-associated *ANO4* variants were identified as homologous nucleotide sites in mouse *Ano4* by sequence alignment between human *ANO4* and mouse *Ano4* (Figure S3). *ANO4* variants were inserted by site-directed mutagenesis into the full-length *Ano4* background. Site-directed mutagenesis was accomplished using the QuickChange site-directed mutagenesis kit (Agilent Technologies, Santa Clara, CA, USA), according to the manufacturer's recommendation. The sequences of primer pairs are shown in Table 1. *ANO4* mutants Met563Lys, Asn558Lys, Ile562Phe, Asn603Asp, Asn129Lys, Val528Met, and Ile725Thr were generated. All final mutagenized *ANO4* constructs were fully sequenced before expression to confirm the accuracy of targeted mutagenesis and to exclude any unexpected sequence alterations.

### Patch-clamp recordings

Membrane currents of wild-type or mutant *ANO4* transfected cells were measured in the whole-cell configuration of the patch-clamp technique. Recordings were performed using single transfected cells that were identified by detection of co-transfected GFP fluorescence. Transfected HEK293 cells were kept in a serum-free medium for 24 h prior to patch-clamp analysis. Patch pipettes were pulled from borosilicate glass capillaries with a pipette resistance ranging from 3 to 5 MΩ using a DMZ Universal Puller (Zeitz, Augsburg, Germany). Whole-cell currents were recorded with an EPC 9 patch-clamp amplifier, and data acquisition and analysis were performed with TIDA hardware and software (HEKA Electronics, Lambrecht, Germany). Current signals were filtered at 2.9 kHz with a low-pass Bessel filter. The mean membrane capacitance was 21 pF ($n = 77$). Access resistance was compensated for a voltage error of 3 mV per 1nA. While recording, the 15 mm glass cover slips with transfected cells were superfused with an extracellular Ringer solution containing (in mM): NaCl 145, $KH_2PO_4$ 0.4, $K_2HPO_4$ 1.6, and Ca-gluconate 1.3. Pipettes were filled with a pipette solution containing 95 mM K-gluconate, 30 mM KCl, 4.8 mM $Na_2HPO_4$, 1.2 mM $NaH_2PO_4$, 0.3 mM Ca-gluconate, and 1 mM EGTA. The pH value of both solutions was adjusted to 7.2 by Tris base. Potentials were corrected for a liquid junction potential of +10.7 mV.

### Immunostaining and co-localization analysis

24 h after transfection, HEK293 cells were fixed with 4% (w/v) paraformaldehyde for 10 min and then washed three times with

Table 1. **The missense variants were generated using wild-type *ANO4* as template and the following primers**

| | |
|---|---|
| Met563Lys | F: GCACATTCAGCAGCTTAATGATACAAAAGTTGATGCATACTG |
| | R: CAGTATGCATCAACTTTTGTATCATTAAGCTGCTGAATGTGC |
| Asn558Lys | F: GCAGCATAATGATACAAAACTTGATGCATACTGCAGTCC |
| | R: GGACTGCAGTATGCATCAAGTTTTGTATCATTATGCTGC |
| Ile562Phe | F: AGAGCACATTCAGCAGCATAAAGATACAAAAGTTGATGCATAC |
| | R: GTATGCATCAACTTTTGTATCTTTATGCTGCTGAATGTGCTCT |
| Asn603Asp | F: GTAAAAAGTGGAGCTGTTCAGATCGACAAACTGAAAAAGAAACATTT |
| | R: AAATGTTTCTTTTTCAGTTTGTCGATCTGAACAGCTCCACTTTTTAC |
| Asn129Lys | F: CTTTTCAGTCTGAGGCTTGGATTTTCTGTACACAAGAATG |
| | R: CATTCTTGTGTACAGAAAATCCAAGCCTCAGACTGAAAAG |
| Val528Met | F: GGATCGTCATTTACAGGATGGTGACTGTGAGCACT |
| | R: AGTGCTCACAGTCACCATCCTGTAAATGACGATCC |
| Ile725Thr | F: GAAAAGCTGCCACAAAGGTCGTTGTGAATCCGAAC |
| | R: GTTCGGATTCACAACGACCTTTGTGGCAGCTTTTC |

1 × Tris-buffered saline (TBS), permeabilized with 0.5% (v/v) Triton X-100 for 10 min at room temperature. After three washes in TBS, cells were then incubated with a blocking solution (5% bovine serum albumin in TBS) for 45 min. Cells were co-labeled overnight at 4°C with primary antibody against ANO4 (rabbit polyclonal, 1:100; Abcam, ab170008) and primary antibody against pan-cadherin (mouse polyclonal, 1:200; Abcam, ab6528), which was used to stain the cell membrane, or primary antibody against early endosome antigen 1 (EEA1) (sheep polyclonal, 1:100; R&D, AF8047). After a 45 min incubation with fluorescence conjugated appropriate species-specific secondary antibodies, donkey anti-rabbit Alexa Fluor 546 (1:1000; Invitrogen by Thermo Fisher Scientific, A10040) and donkey anti-mouse Alexa Fluor 647 (1:1000; Invitrogen by Thermo Fisher Scientific, A-31571) or donkey anti-sheep Alexa Fluor 488 (1:1000; Invitrogen by Thermo Fisher Scientific, A-11015), DAPI (4′,6-diamidino-2-phenylindole) (Sigma-Aldrich, Co, St. Louis, USA), was used to stain nuclear DNA. Cells were mounted onto glass slides with a fluorescence mounting medium (Dako, Germany) and examined by confocal microscopy (Leica SPE, Germany). Confocal fluorescence microscopy images were quantitatively analyzed using an ImageJ software package (Rasband, W.S., ImageJ, US National Institutes of Health, Bethesda, Maryland, USA, 1997–2015). The quantitative co-localization analysis of ANO4 and pan-cadherin or EEA1 were performed by Pearson's correlation coefficient (PCC) to evaluate the degree of co-localization. Values of PCC range from −1 to 1, where a negative value (−1) indicates no overlap, and 1 is a complete co-localization.

### Measurement of scramblase activity

To explore the influence of mutant ANO4 on scramblase activity, PS exposure was examined by Fluorescence-Activated Cell Sorting (FACS). Annexin A5 is a $Ca^{2+}$-binding protein that can bind to PS, and therefore fluorescently labeled annexin A5 can be used to detect PS exposed to the outside of the cells. Scramblase activity was determined by measuring the fluorescently labeled annexin A5 binding to exposed PS. ANO4 mutant transfected HEK293 cell pellets (0.5–1 × $10^6$ cells) were collected and divided equally into two tubes, one of which was treated with 1 μM ionomycin for 10 min. After washing once with 500 μL PBS, the cells were resuspended in 500 μL annexin A5 binding buffer (1.3 mM $CaCl_2$, 10 mM HEPES, 150 mM NaCl, 5 mM KCl, 1 mM $MgCl_2$, pH 7.4), each sample was incubated with 0.25 μg of cys-anxA5-6S-IDCC in the dark for 10 min. Cys-anxA5-6S-IDCC comes from cys-annexin-A5 (NeXins Research, the Netherlands) labeled with maleimide-6S-IDCC (Mivenion GmbH, Berlin, Germany) at the singular cysteins as described elsewhere.[73] Data acquisition and analysis of annexin A5 binding and GFP-expression were performed using a BD Accuri C6 flow cytometer with BD Accuri C6 software (BD Biosciences, Heidelberg, Germany), according to the manufacturer's instructions.

### Statistical analysis

All experiments were repeated independently at least three times, and one representative result from each group was displayed. Mean values were given as mean ± SEM; n represents the number of experiments. Statistical differences between groups were tested using GraphPad Prism (GraphPad Software, Inc, USA) by multicomparisons with ANOVA and post hoc analysis (either Dunn's or Tukey's multiple comparisons post hoc test) with confidence intervals of 0.05. $p$ values of less than 0.05 were considered statistically significant. $p$ values are indicated in all figures according to convention: *$p < 0.05$; **$p < 0.01$; ***$p < 0.001$; ****$p < 0.0001$; and ns, not significant.

## Results

### Study cohort and genetic variants

Clinical details and nomenclature of detected variants are provided in Tables 2, 3, and 4 and the case reports in the supplemental note.

I1–I5 were sporadic cases diagnosed with developmental and epileptic or epileptic encephalopathy (DEE/EE) that harbored the following heterozygous *de novo ANO4* missense variants, respectively: I1, c.1688T>A (p.Met563Lys)

**Table 2. Overview of observed *ANO4* variants in our study cohort**

| Individual | I1 | I2 | I3 | I4 | I5 | F6 | I7 |
|---|---|---|---|---|---|---|---|
| *ANO4* variant on gDNA level on NC_000012.11 (hg19) | g.101480589T>A | g.101480575C>A | g.101480585A>T | g.101490382 A>G | g.101336244C>G | g.101480483G>A | g.101504206T>C |
| cDNA level on NM_001286615.1 | c.1688T>A | c.1674C>A | c.1684A>T | c.1807A>G | c.387C>G | c.1582G>A | c.2174T>C |
| Protein level on NP_001273544.1 | p.Met563Lys | p.Asn558Lys | p.Ile562Phe | p.Asn603Asp | p.Asn129Lys | p.Val528Met | p.Ile725Thr |
| ClinVar[a] | SCV004809186 | SCV004809187 | SCV004809188 | SCV004809189 | SCV004809190 | SCV004809191 | SCV004812191 |
| rs number (dbSNP) | unreported | unreported | unreported | unreported | unreported | unreported | rs200708403 |
| Allele frequency (gnomAD v.2.1.1) | unreported | unreported | unreported | unreported | unreported | unreported | All: 0.00081% - NFE: 0.0018% |
| Amino acid conservation | highly conserved | highly conserved | highly conserved | highly conserved | highly conserved | highly conserved | highly conserved |
| *In silico* prediction tools[b] | SIFT: deleterious, MutationTaster: deleterious, PolyPhen2: possibly damaging, CADD: 28.4, AlphaMissense: 0.9899 | SIFT: deleterious, MutationTaster: deleterious, PolyPhen2: probably damaging, CADD: 24.9, AlphaMissense: 0.9982 | SIFT: deleterious, MutationTaster: deleterious, PolyPhen2: probably damaging, CADD: 29.4, AlphaMissense: 0.9521 | SIFT: deleterious, MutationTaster: deleterious, PolyPhen2: probably damaging, CADD: 27.8, AlphaMissense: 0.9928 | SIFT: deleterious, MutationTaster: benign, PolyPhen2: benign, CADD: 19.15, AlphaMissense: 0.4986 | SIFT: deleterious, MutationTaster: deleterious, PolyPhen2: possibly damaging, CADD: 25.8, AlphaMissense: 0.6859 | SIFT: deleterious, MutationTaster: benign, PolyPhen2: probably damaging, CADD: 27.1, AlphaMissense: 0.9291 |
| Inheritance | *de novo* | *de novo* | *de novo* | *de novo* | *de novo* | AD inheritance with 73% penetrance | inherited from asymptomatic mother |
| Genetic testing revealing *ANO4* variant | trio WES | trio WES | trio WES | trio WES | trio WES | linkage + WGS | candidate gene screening |
| Previous genetic testing | chromosomal microarray normal, skewed X-inactivation 15:85 | standard karyotype normal, chromosomal microarray normal, epilepsy panel normal | chromosomal microarray normal, epilepsy panel normal | chromosomal microarray normal, karyotype normal, SCN1A sequencing + MLPA normal | chromosomal microarray with paternal duplication of 132 kb in 21q22.3 | – | chromosomal microarray normal |
| Other relevant genetic findings | compound heterozygous variants in *GJB2*c.35del (GenBank: NM_004004.5) and c.101T>C potentially associated with late onset hearing loss | none | – | – | heterozygous variant in *SLC25A22* associated with autosomal recessive DEE | – | inherited heterozygous *GCH1* gene deletion associated with Dopa-responsive dystonia |

AD, autosomal dominant; dbSNP, Single-Nucleotide Polymorphism Database; gnomAD, The Genome Aggregation Database; SIFT, sorting intolerant from tolerant; CADD, combined annotation dependent depletion; NFE, non-Finnish European; WES, whole-exome sequencing; WGS, whole-genome sequencing.
[a]ClinVar accession number of record submitted by the authors.
[b]SIF, MutationTaster, PolyPhen2, and CADD scores as provided by Alamut Visual Plus v1.7.1 on June 21, 2023 (Sophia Genetics SA, Lausanne, Switzerland).

**Table 3. Clinical features of individuals with disease-associated *ANO4* variants (I1-7) and a likely benign deletion (DECIPHER ID 272667)**

| Individual | I1 | I2 | I3 | I4 | I5 | F6 | I7 | DECIPHER ID 272667 |
|---|---|---|---|---|---|---|---|---|
| *ANO4* variant (on NP_001273544.1) | p.(Met563Lys) | p.(Asn558Lys) | p.(Ile562Phe) | p.(Asn603Asp) | p.(Asn129Lys) | p.(Val528Met) | p.(Ile725Thr) | 12q23.1 deletion of 0.3 Mb affecting *ANO4* |
| Inheritance | de novo | de novo | de novo | de novo | de novo | AD inheritance with 73% penetrance | inherited from asymptomatic mother | inherited from healthy father |
| Assumed pathogenicity | Pathogenic | Pathogenic | Pathogenic | Pathogenic | Pathogenic | Pathogenic | Likely pathogenic | Likely benign |
| Individual previously reported | phenotype reported in Papuc et al. 2019[3] | - | - | - | - | Depondt et al. 2002[66] & Claes et al. 2004[67] (pedigree and linkage) | - | - |
| Parental consanguinity | no | no | no | no | no | no | no | |
| Ethnicity | White (Swiss) | White (French) | White (French) | White (Finnish) | White (Dutch) | White (Belgian) | White (Belgian) | |
| Other affected family members | none | none | none | none | none | 1 family with 23 affected individuals | brother of maternal grandmother died of severe epilepsy at age 16y | no |
| Age at last investigation | 12 2/12y | 7y deceased at age 12y 6m due to neurologic and respiratory decompensation | 7y | 22y | 23y | | 39y | 7y |
| Sex | male | male | male | male | male | | female | |
| Gestational age (weeks) | 41 3/7 | 41 | 39 4/7 | 41 | 36 3/7 | | NA | |
| Pre-/perinatal issues | none | none | none | none | premature, otherwise normal | | no | maternal hypertension |
| Growth parameters at birth (L/W/OFC) | z +0.64 / + 0.67 / + 0.99 | z -1.57 / -0.97 / +0.08 | z -0.65 / -0.44 / -0.85 | z +0.39 / +0.27 / +0.46 | NA / z +0.02 / NA | | NA | |
| Growth parameters at last examination (L/W/OFC) | NA / z -1.51 / -1.75 | z -2.22 / -0.16 / -4.26 | z -4.11 / -1.84 / -3.2 | z -1.36 / -2.14 / NA | z +0.4 / NA / +1.9 | | NA | normal |
| Microcephaly | no | yes, secondary | yes, secondary | no | no | | no | |
| Morphological features | pectus excavatum, wide-spaced teeth | severe narrow upper jaw with a | no | hypertelorism, high nasal bridge, thin | hypertelorism, striae | | no | |
| | | retromaxilla, an ogival palate and some misaligned teeth, moderate macroglossia | | upper lip, wide-spaced incisors | | | | |
| Initially normal development | no | no | no | yes | yes | | | |
| Problems in early infancy | hypotonia, floppy infant | hypotonia/ feeding problems | hypotonia | no | no | | no | no |
| Developmental regression | no | no | less interactions after first seizures | stagnation/slowing at 2-3y | yes, after age 7y | no | no | |
| Developmental delay | profound delay | profound delay | profound delay | yes, obvious during 2nd year of life | yes | no | no | yes |
| Age at sitting | not achieved | not achieved | 13m | NA | 6m | | | |
| Age at walking | not achieved | not achieved | not achieved | 15m | 14m | | | 19m |
| Age at first words | not achieved | not achieved | not achieved | 1.5y | 12m | | | |
| Speech | non-verbal | non-verbal | non-verbal | two-word expressions, echolalia | normal until age 7y | | | non-verbal |
| ADLs | not achieved | not achieved | not achieved | yes | assisted living facility | | | |
| Intellectual disability | severe ID | profound ID | severe ID | moderate-severe ID | moderate ID, IQ 100 at 4y 9m, IQ 51 at 14y | no | no | |
| Behavioral problems | no | no | no | autistic features, attention deficit, aggressivity | ASD | no | no | ASD |
| Epilepsy classification | DEE | DEE | DEE | EE | childhood-onset EE | TLE/GEFS+ | TLE | none |
| Brain imaging result / age | unspecific white matter hyperintensities (3m, 2y), elevated lactate peak parietal left (3m) | normal (29d), severely delayed myelinization (1y 2m), diffuse hypomyelination, cortical and cerebellar atrophy (2y 7m) | 1st cMRI normal; right frontal dysplasia (3y) | normal (1y), incomplete myelination (temporal lobes not fully myelinated) (3y), normal (9y) | parietal periventricular leukomalacia, cerebral and cerebellar atrophy (16y) | variable: normal / hippocampal malrotation / cortical dysplasia / nonspecific anomalies | unspecific white matter lesion in right frontal lobe (23y) | |
| Muscle tone | severe hypotonia of the trunk and limbs | severe hypotonia of the trunk, severe hypertonia of limbs | hypotonia | hypotonia | hypotonia | | normal | |
| Other neurological issues | horizontal nystagmus, abnormality of ocular smooth pursuit, ataxia, dysmetria, myoclonus, hyperkinesia | eyelid myoclonia with conjugate eye deviation, dystonia | no | hyperkinesia | tremor | | Dopa responsive dystonia due to *GCH1* deletion | |
| Skeletal | funnel chest | severe scoliosis causing ventilation difficulties, osteopenia, left hip | scoliosis | pes planus, hyperextensible joints, crouch position | no | | no | |
| | | dislocation, joint contractions, feet deformation | | | | | | |
| Gastrointestinal | feeding difficulties in early childhood, reflux, constipation | gastrostomy | gastrostomy | no | no | | no | |
| Hearing loss | no | no | no | no | yes, asymmetric | | no | |
| Ophthalmological | hypermetropia, slightly reduced visus, immature maculae, strabism | abnormal visual potential, bilateral optic atrophy | strabism | no | enlarged c/d-ratio of optic disc | | no | |
| Pulmonary | recurrent respiratory infections, chronic cough, snoring | hypercapnia requiring oxygen therapy due to scoliosis | no | no | no | | no | |
| Sleep disturbances | sleeps a lot, difficult to wake up | obstructive sleep apnea | no | yes | sleep apnea with CPAP therapy | | no | |
| Other anomalies | central hypothyroidism | pyelonephritis, kidney and bladder stones, ectopic testes, hypercalciuria, hypophosphatemia | NR | no | muscle biopsy: reduced mitochondrial energy production, without mtDNA mutations | | coarctatio aortae | |
| Comments | hippotherapy led to improved truncal muscle tone and related functions. Content and friendly disposition | social smiles and eye contact were present | | | low energy level | four family members died of prolonged seizures or SUDEP | | |

ADL, activities of daily living; ASD, autism spectrum disorder; cMRI, cerebral magnetic resonance imaging; CPAP, continuous positive airway pressure; DEE, developmental and epileptic encephalopathy; EE, epileptic encephalopathy; GEFS+, genetic epilepsy with febrile seizures plus; H, height; ID, intellectual disability; IQ, intelligence quotient; L, length; Mb, megabases; NA, not assessed; NR, not reported; OFC, occipitofrontal circumference; SUDEP, sudden unexplained death in epilepsy; TLE, temporal lobe epilepsy; W, weight.

**Table 4. Characterization of epilepsy phenotypes associated with *ANO4* variants**

| Individual | I1 | I2 | I3 | I4 | I5 | F6 | I7 |
|---|---|---|---|---|---|---|---|
| *ANO4* variant (on NP_001273544.1) | p.(Met563Lys) | p.(Asn558Lys) | p.(Ile562Phe) | p.(Asn603Asp) | p.(Asn129Lys) | p.(Val528Met) | p.(Ile725Thr) |
| Inheritance | de novo | de novo | de novo | de novo | de novo | AD inheritance with 73% penetrance | inherited from asymptomatic mother |
| Epilepsy classification | DEE | DEE | DEE | EE | childhood-onset EE | GEFS+ | TLE |
| Age of seizure onset | 2m | 1m | 7m | 11m | 41m | 9m – 35y | 18y |
| Seizure type | multifocal clonic, GTCS atonic, | focal impaired awareness, GTCS | focal, GTCS | febrile seizures, focal impaired awareness, tonic, GTCS, myoclonia | febrile seizures, tonic, GTCS, absences, myoclonia, focal impaired awareness, frequent nocturnal seizures | focal aware, focal impaired awareness, GTCS | focal impaired awareness |
| Seizure provoking factors | hyperthermia | hyperthermia, pain | hyperthermia, fatigue | hyperthermia, exercise | hyperthermia | hyperthermia | none |
| Fever sensitivity | yes | yes | yes | yes | yes | yes | no |
| Current seizure frequency | 2x/week | 2 to 10 per day | 10 per year, focal with possible evolution to GTCS | seizure-free since age 17y | GTCS 5x, tonic 5x, absences/myoclonia 35x/week | seizure-free with or without AED / infrequent / refractory | seizure-free |
| Current antiepileptic treatment | VPA, OXC, CLB | VPA, LEV, CLB | VPA, TPM, STP, CLP | VPA, TPM | VPA, RFM, CLB, vagus nerve stimulation | | CBZ |
| Previous antiepileptic treatment | PB, LEV, LTG, Cannabidiol oil | PB, VGB, CLN, PHT, TPM, ketogenic diet (not efficient) | OXC, CBZ (aggravation) | OXC, LEV, LTG, STP, RFM, Bromide (good response) | ketogenic diet (temporarily beneficial), LEV, TPM, ZNS (all not beneficial), PHT, LCM | | none |
| EEG results | rare sharp waves median frontal and central region as well in the right centroparietal region in sleep-EEG (3m); normal (7m, 1y, 2y 3m, 2y 10m); multifocal sharp waves, polyspike waves and abnormal background activity (at multiple times during follow up) | normal (1m, 4m, 12m); slow background and multifocal spikes/polyspike waves starting at 13m and still at 5y | initially little or no interictal anomaly. Recording of a migrating crisis (1y); centrotemporal points (3y) | normal (1y); multifocal spikes/polyspike waves, slow background (1y 5m); focal onset ictal discharges (3y, 9y) | background slowing, multifocal epileptiform discharges | typical temporal lobe seizures in some, generalized epileptic discharges in some | normal (23y) |

AED, antiepileptic drug; CBZ, carbamazepine; CLB, clobazam; CLP, clonazepam; DEE, developmental and epileptic encephalopathy; EE, epileptic encephalopathy; EEG, electroencephalography; GEFS+, genetic epilepsy with febrile seizures plus; GTCS, generalized tonic clonic seizures; LCM, lacosamide; LEV, levetiracetam; LTG, lamotrigine; OXC, oxcarbazepin; PB, phenobarbital; PHT, phenytoin; RFM, rufinamide; STP, stiripentol; TLE, temporal lobe epilepsy; TPM, topiramate; VPA, valproic acid; ZNS, zonisamide.

(M563K); I2, c.1674C>A (p.Asn558Lys) (N558K); I3, c.1684A>T (p.Ile562Phe) (I562F); I4, c.1807A>G (p.Asn603Asp) (N603D); and I5, c.387C>G (p.Asn129Lys) (N129K). All detected variants are not reported in the Genome Aggregation Database (gnomAD v.2.1.1) and the Single-Nucleotide Polymorphism Database (dbSNP) and affect a highly conserved amino acid (Figure S3). *In silico* prediction tools SIFT (sorting intolerant from tolerant),[74] MutationTaster,[75] PolyPhen2,[76] and AlphaMissense[77] predict a deleterious or possibly/probably damaging effect for variants detected in I1–I4, while predictions for c.387C>G (p.Asn129Lys) are ambiguous. The phenotype was classified as DEE in I1–I3 and as EE in I4 and I5. Seizure types were variable, including both focal and generalized seizures, and began in the first year of life in individuals I1–I4 and at 41 months of age in I5. Reported seizure-provoking factors were hyperthermia in all and pain, fatigue, or exercise in one individual each. Four of five DEE/EE individuals are not seizure-free despite multiple antiepileptic medications, with a seizure frequency ranging from several per day to several per year. I4 has been seizure-free since age 17 years with valproic acid and topiramate. He previously showed a good response to bromide. Aggravation with carbamazepine occurred in one individual. All five also have mild to severe muscular hypotonia. Cranial MRI revealed nonspecific changes such as atrophy, hypomyelination, or bilateral periventricular leukomalacia in all five individuals with DEE/EE, though initial scans were sometimes reported as normal. Two of five had secondary microcephaly. Hyperkinesia was described in two of five and tremor in one child. Development was profoundly impaired in three of five, with no independent skills in activities of daily living. Language development was also severely impaired in four of five (non-verbal or few words only). I4 showed normal early development with stagnation occurring by the age of 2–3 years. He had moderate ID in formal neuropsychological examinations and was able to perform activities of daily living during childhood, then regressed in early adulthood to severe ID and needing constant supervision. I5 developed age appropriately until about 7 years old and then regressed with increased seizure activity; at age 14, he had an IQ of 51. Motor development was variable, ranging from lack of independent sitting and walking to motor milestones within the normal range. Behavioral difficulties such as autistic features, attention deficit, or aggressiveness were described in two affected individuals. Scoliosis developed in two individuals; a funnel chest was observed in one. Eye anomalies occurred in three of five individuals and included bilateral optic atrophy, strabismus, and hypermetropia. One child had asymmetric hearing loss. Feeding difficulties were reported in two children, one of which required a gastrostomy. One child had hypothyroidism, susceptibility to infection, and increased need for sleep. Two were diagnosed with sleep apnea. There was no specific recognizable morphological phenotype, though

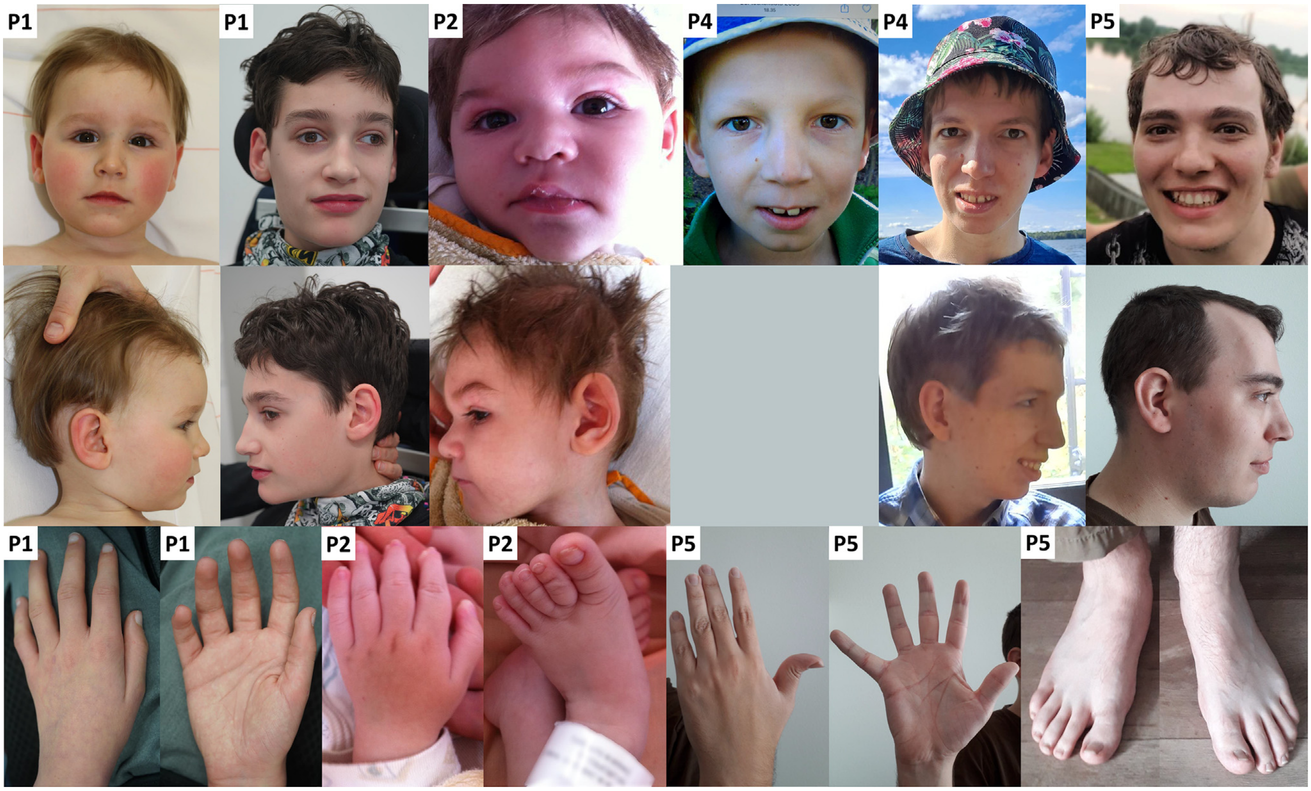


**Figure 1. Morphology of individuals with *ANO4* variants**
Facial appearances of four individuals with *de novo* variants in *ANO4* associated with DEE or EE (I1 at age 12 months and 12 years 2 months, I2 at age 15 months, I4 at age 9 years, 18 years, and 20 years, respectively, and I5 at age 22 years 9 months) and hands/feet of three individuals (I1 at age 12 years 2 months, I2 at age 15 months, and I5 at age 22 years 9 months).

shared features included apparent hyper- or hypotelorism, long nose with bulbous tip, and a thin upper lip (Figure 1).

Family 6 (F6) consists of a large pedigree of 23 affected individuals with TLE or GEFS+ (Figure S2) in which the heterozygous *ANO4* variant c.1582G>A (p.Val528Met) (V528M) was segregating in an autosomal-dominant manner. One individual had only febrile seizures, 11 only afebrile seizures, and ten had a combination of febrile and afebrile seizures. Most affected family members had focal onset seizures, and ten had clear TLE. Two had generalized epilepsy with generalized epileptic discharges on electroencephalography (EEG). Although the family was at that time reported as having familial TLE with febrile seizures, nowadays it would be classified as GEFS+ given the presence of different seizure types within the same family. Three family members died of prolonged febrile seizures, and one of sudden unexplained death in epilepsy (SUDEP). The identified segregating ANO4 variant was present in all 20 affected family members for whom DNA was available and had a 73% penetrance (22 affected [obligate] carriers/30 total [obligate] carriers). I7 had normal neurodevelopment and adult-onset TLE. The heterozygous variant c.2174T>C (p.Ile725Thr) (I725T) was inherited from the unaffected mother who had a maternal uncle who died of epilepsy at the age of 16 years. Maternal grandparents were not available for further segregation testing.

The individual with DECIPHER ID 272667 harbored the 307.65-kb-sized deletion (g.101184062-101491712 [GenBank: NC_000012.11] [GRCh37]) affecting exons 1–19 of 28 of *ANO4* (GenBank: NM_001286615.1), likely leading to a complete loss of allele product. This deletion was inherited from the healthy father, and therefore it was considered of unknown significance for the individual's phenotype. After an uneventful pregnancy and postnatal period, the individual showed developmental delay and was diagnosed with autism spectrum disorder. The child remained nonverbal at age 7 years. There is no history of seizures, and growth parameters were normal.

## Homology modeling of ANO4 variants

Mapping of the variants according to the published experimentally determined transmembrane domains (TMs)[5] is provided in Figure 2A. To better understand the effects of the missense variants on ANO4, we performed a structural protein analysis. Since no experimental structure of ANO4 is available yet, we used a model generated by AlphaFold-2 for the structural interpretation of the variants. All seven variants discovered in the present study are located in the globular part of ANO4 (Figure 2B), and our analysis suggests that they destabilize the ANO4 structure. The most prominent effects involve DEE/EE variants and are

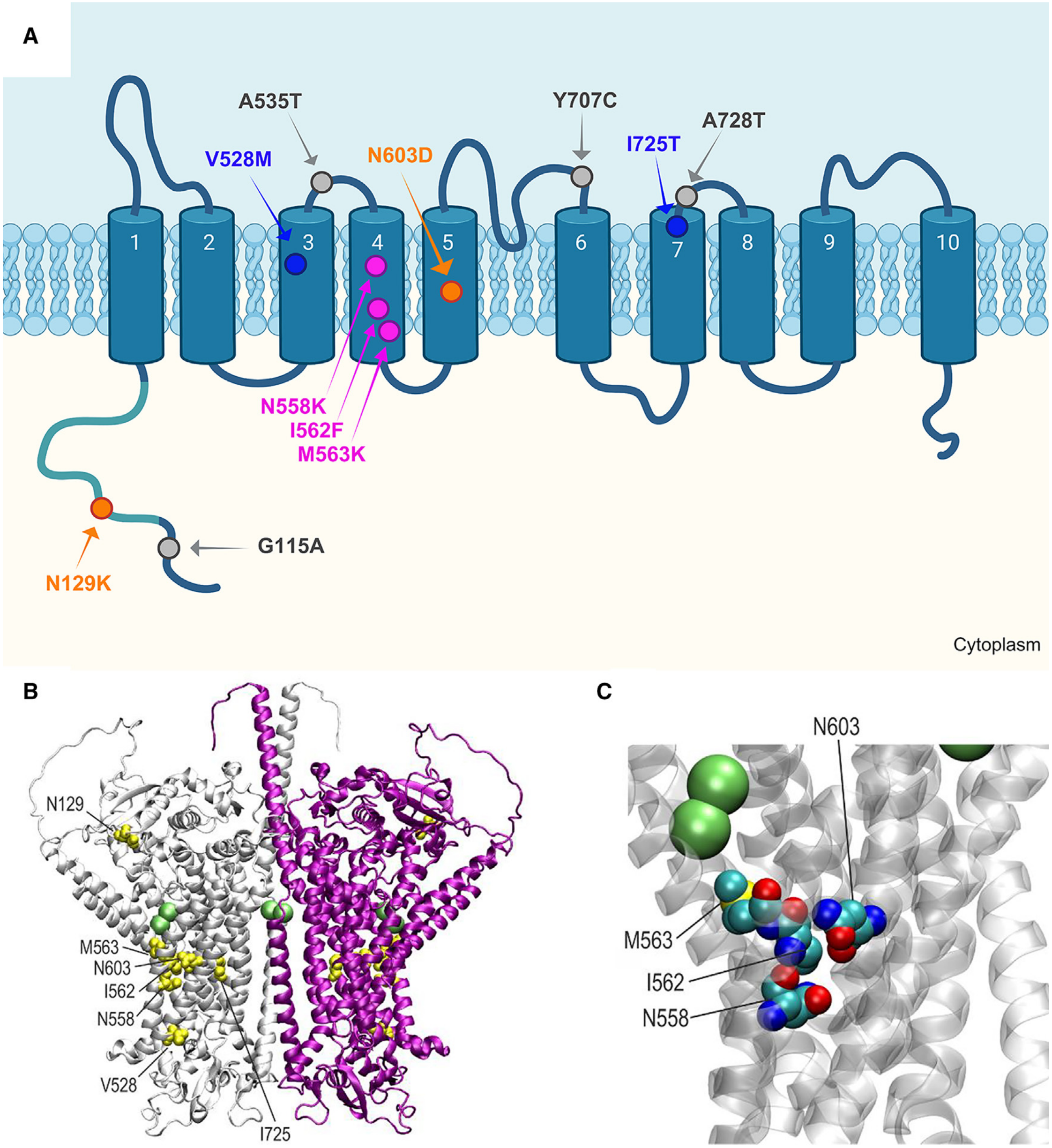


**Figure 2. Membrane topology and 3D structure of ANO4 indicating the disease-associated variants and selected population variants**
(A) Membrane topology of ANO4 based on experimental data in the *N. haematococca* ortholog, drawn using Biorender software. The TMs numbered from 1 to 10 are represented with barrels and located in the plasma membrane. The N-terminal dimerization domain is labeled in turquois. The disease-associated variants are colored according to the phenotype: Asn558Lys (N558K), Ile562Phe (I562F), Met563Lys (M563K) in red with DEE; Asn603Asp (N603D) and Asn129Lys (N129K) in orange with EE; and Val528Met (V528M) and Ile725Thr (I725T) in blue with TLE/GEFS+. The most frequent population variants with deleterious *in silico* predictions Gly115Ala (G115A), Ala535Thr (A535T), Tyr707Cys (Y707C), and Ala728Thr (A728T) are depicted in gray.
(B) Overall structure of the ANO4 dimer with the subunits shown as white and purple ribbon. Residues affected by mutation are shown in yellow space-filled presentation and are labeled for one subunit. The position of bound $Ca^{2+}$ ions is indicated by green balls. The disordered residues 1–40 have been omitted for clarity.
(C) Enlargement of the ANO4 region that represents a mutational hotspot in the present study. The residues affected by mutation are shown in space-filled representation (atom-type coloring) and are labeled. The position of adjacent $Ca^{2+}$ ions is indicated by green balls.

steric clashes due to the appearance of larger sidechains (e.g., Ile562Phe, Asn129Lys, Asn558Lys) and/or the emergence of novel charges (e.g., Met563Lys, Asn129Lys, Asn558Lys, Asn603Asp). Of note, four DEE/EE variants (Asn558Lys, Ile562Phe, Met563Lys, and Asn603Asp) cluster in the immediate spatial proximity of a $Ca^{2+}$ ion binding site (Figures 2B and 2C).

### Functional studies

The results of functional studies are summarized in Table 5.

### Mutant ANO4 show reduced $Ca^{2+}$-dependent cation conductance and plasma membrane localization

To investigate whether disease-associated *ANO4* variants alter $Ca^{2+}$-dependent cation conductance, whole-cell

**Table 5. Summary of results of functional studies**

| | | | Heterogenic expression analysis | | | | | |
|---|---|---|---|---|---|---|---|---|
| | | | | | | | Apoptosis | |
| Individual | ANO4 mutant | Phenotype | Basal conductance | $Ca^{2+}$-dependent cation conductance | Membrane location | Colocalization with EEA1 | − Ionomycin | + Ionomycin |
| I1 | Met563Lys | DEE | ns | ↓**** | ↓* | ↓**** | ↑* | ns |
| I2 | Asn558Lys | DEE | ns | ↓**** | ↓**** | ↓**** | ↑** | ns |
| I3 | Ile562Phe | DEE | ↓* | ↓**** | ↓**** | ↓*** | ↑** | ns |
| I4 | Asn603Asp | EE | ns | ↓**** | ↓**** | ↓**** | ns | ns |
| I5 | Asn129Lys | EE | ns | ↓**** | ↓**** | ↓**** | ns | ns |
| F6 | Val528Met | GEFS+ | ns | ↓**** | ns | ↓*** | ns | ns |
| I7 | Ile725Thr | TLE | ↓** | ↓**** | ns | ns | ns | ns |
| I2 | WT/ Asn558Lys | DEE | ↓** | ↓**** | ns | ↓**** | ↑**** | not tested |
| F6 | WT/ Val528Met | GEFS+ | ↓*** | ↓**** | ↓*** | ↓**** | ↑*** | not tested |

DEE, developmental and epileptic encephalopathy; EE, epileptic encephalopathy; EEA1, early endosome antigen 1; GEFS+, genetic epilepsy with febrile seizures plus; ns, not significant; TLE, temporal lobe epilepsy; WT, wild-type. *p $< 0.05$; **$p < 0.01$, ***$p < 0.001$, ****$p < 0.0001$.

patch-clamp recordings were performed on HEK293 cells that heterologously express either wild-type ANO4 or one of the ANO4 mutants. To ignite $Ca^{2+}$-dependent cation membrane conductance, intracellular free $Ca^{2+}$ was increased by the extracellular application of the $Ca^{2+}$ ionophore ionomycin (1 μM). In the first step, we verified the basal properties of wild-type ANO4 and transfection controls (Figures 3A–3C, and 3D). Cells overexpressing wild-type ANO4 showed a robust current activation after application of 1 μM ionomycin (Figure 3A). In control experiments with GFP-transfected cells (Figure 3C) or untransfected cells (Figure 3D), we saw weak activation of currents after ionomycin application that were, however, much smaller than those with wild-type ANO4. In the second step, we investigated the $Ca^{2+}$-dependent cation membrane conductance in cells transfected with ANO4 mutants. Thereby, we noted that the baseline of current amplitude was significantly lower in mutants Ile562Phe and Ile725Thr compared to wild-type ($p < 0.05$) and similar to the control baselines. The other ANO4 mutants showed no significant difference in the baseline of current amplitude between the membrane potentials of −140 mV to +60 mV (Figure 3E). After application of ionomycin, all ANO4 mutants showed a relatively mild increase in current amplitude with an increased membrane conductance after a latency of approximately 100 s that turned the holding current at −40 mV into an inward current corresponding with cell depolarization and weak or no rectification (Figures 3F–3H, 3J, 3L, 3N, 3P, and 3R). Given our experimental conditions with a difference of extracellular $Cl^-$ (145 mM) and intracellular $Cl^-$ (30 mM), these findings correspond with the activation of a non-selective cation current because activation of $Cl^-$ channels would not change the holding current (holding potential equals the Nernst potential for $Cl^-$) and would show outward rectification.

For quantitative statistical comparison, we estimated the amplitude of ionomycin-induced steady-state currents and calculated the fold current increase in response to ionomycin (Figure 4A). Ionomycin application resulted in a 1- to 2.5-fold increase in current density in all ANO4 mutants, which was significantly lower compared to the 5-fold increase in wild-type ANO4 ($p < 0.0001$) (Figure 4A) suggesting a loss of channel function for all mutants. Of note, ANO4 mutant Asn603Asp showed the smallest decrease in current density of all mutants to about half of that of wild-type ANO4.

To confirm that these results are indeed due to anoctamin dysfunction, we applied flufenamic acid (100 μM), a broad range blocker for ANOs, to the extracellular solution once the currents reached a steady-state value in response to 1 μM ionomycin. The ionomycin-activated currents of both wild-type and mutant *ANO4* transfected cells were sensitive to flufenamic acid, indicating that the observed currents depend on ANOs (Figure 5).

To investigate whether the observed loss-of-function (LoF) effect occurs through malfunction of the pore or reduced trafficking into the plasma membrane, we assessed plasma membrane trafficking using immunocytochemistry (Figures 3B–3G, 3I, 3K, 3M, 3O, 3Q, 3S and S4). As expected, wild-type ANO4 was detected at the plasma membrane as indicated by co-localization with pan-cadherin, resulting in a PCC of 0.568 (Figure 4B). This indicates that 57% of wild-type ANO4 is located at the plasma membrane, and a large proportion was present in the cytosol. Contrarily, the proportion of plasma membrane localization of DEE/EE-associated ANO4 mutants (Met563Lys, Asn558Lys, Ile562Phe, Asn603Asp, and Asn129Lys) was significantly reduced (Figure 4B). Notably, ANO4 mutants Val528Met and Ile725Thr, associated with the less-severe phenotypes of GEFS+ and TLE, didn't show any significant difference to the wild type (Figures 4B and S4).

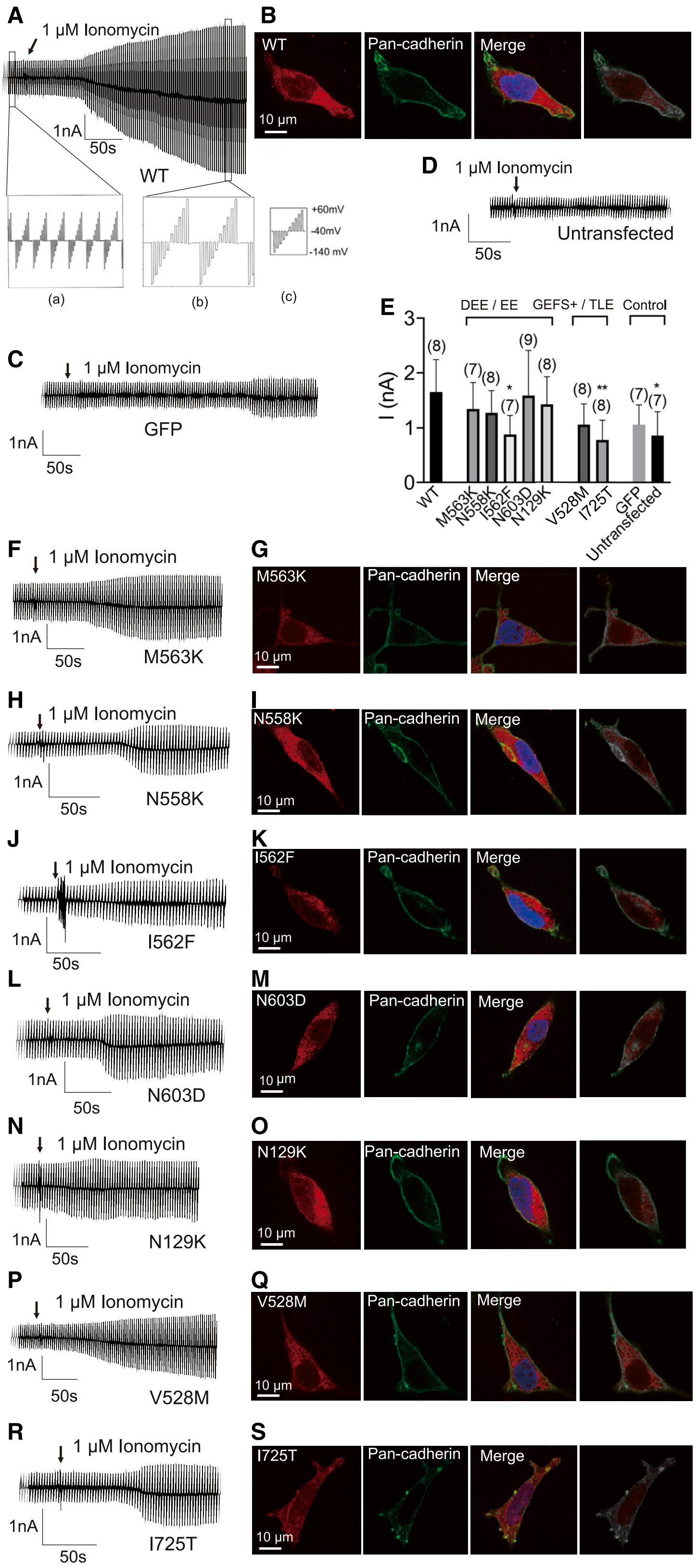


**Figure 3. Analysis of the $Ca^{2+}$-dependent cation conductance of heterologously expressed wild-type and mutant ANO4**

(A) Raw current recording in HEK293 cells expressing wild-type ANO4 before and after application of ionomycin (1 μM; arrow); (a): the baseline currents before ionomycin application, (b): the currents after the application of 1 μM ionomycin, (c): stimulation protocol with 10 voltage steps between −140 mV and +60 mV, for a duration of 50 ms applied every 2.5 s.

(B) Immunostaining of a HEK293 cell expressing wild-type ANO4 against ANO4 (red) and the membrane marker pan-cadherin (green). From the left panel: ANO4 only, pan-cadherin only, merged ANO4 and pan-cadherin, and display of pixels that co-localize red and green in white.

(C and D) Control experiments using cells expressing GFP (C) and untransfected cells (D). Raw currents before and after application of ionomycin (arrow).

(E, F, H, J, L, N, P, and R) Summary of maximal current amplitudes estimated from a voltage difference between −120 mV and +60 mV measured before application of ionomycin to compare basal membrane conductance between controls, wild-type ANO4, and seven mutant ANO4. Raw currents measured in a cell expressing ANO4 mutants Met563Lys (M563K; F), Asn558Lys (N558K; H), Ile562Phe (I562F; J), Asn603Asp (N603D; L), Asn129Lys (N129K; N), Val528Met (V528M; P), or Ile725Thr (I725T; R) before and after ionomycin application (arrow).

(G, I, K, M, O, Q, and S) Immunostaining of a HEK293 cell expressing ANO4 mutants Met563Lys (M563K; G), Asn558Lys (N558K; I), Ile562Phe (I562F; K), Asn603Asp (N603D; M), Asn129Lys (N129K; O), Val528Met (V528M; Q), or Ile725Thr (I725T; S) with antibody against ANO4 (red) and the membrane marker pan-cadherin (green). From the left panel: ANO4 only, pan-cadherin only, merged ANO4 and pan-cadherin, and display of pixels that co-localize red and green in gray.

Values are given as mean ± SEM. Multiple comparisons were performed by ANOVA with Dunn's post hoc test. Data points that were statistical outliers were eliminated by the Grubbs outlier test. *$p < 0.05$; **$p < 0.01$; $n$ is given in parentheses.

DAPI was used to counterstain the nucleus (blue) in (B, G, I, K, M, O, Q, and S).

To investigate whether ANO4 variants affect subcellular trafficking, co-localization with early endosomes was assessed using the early endosome marker EEA1 (Figures 6A–6H). All ANO4 mutants except Ile725Thr showed significantly reduced co-localization with EEA1 compared to wild-type ANO4 ($p < 0.0001$; Figures 6I and S4). Thus, the lower surface expression of mutant ANO4 correlates with reduced trafficking through early endosomes and therefore does not suggest aberrant clogging as pathomechanism.

## Assessment of phospholipid scramblase activity by annexin A5 surface expression in HEK293 cells overexpressing wild-type or mutant ANO4

Since ANO4 may also have phospholipid scramblase activity, we investigated binding of fluorescently labeled annexin A5 to exposed PS. An increased surface expression of PS can indicate activated apoptosis in stressed cells. At physiological levels of intracellular $Ca^{2+}$, cells expressing Met563Lys, Asn558Lys, and Ile562Phe showed significantly increased annexin

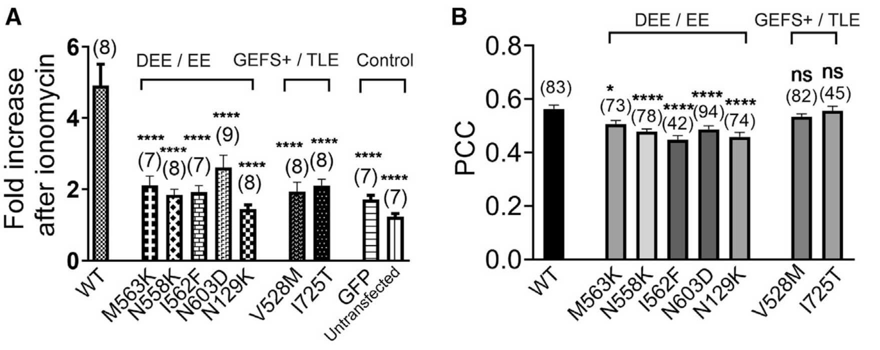


**Figure 4. Comparison of ANO4-dependent membrane conductance and ANO4 surface expression**

(A) Comparison of changes in membrane conductance estimated as currents of a voltage difference between −140 mV and +60 mV from cells with different conditions of heterologous expression after reaching a maximal steady-state level of the ionomycin effect, given as fold increase from baseline.

(B) Comparison of Pearson's correlation coefficient (PCC) of ANO4 and pan-cadherin positive pixels from immunostainings of HEK293 cells expressing ANO4 wild-type or mutants Met563Lys (M563K), Asn558Lys (N558K), Ile562Phe (I562F), Asn603Asp (N603D), Asn129Lys (N129K), Val528Met (V528M), or Ile725Thr (I725T).

Values are given as mean ± SEM. Multiple comparisons were performed by ANOVA with Dunn's post hoc test. Data points that were statistical outliers were eliminated by the Grubbs outlier test. *$p < 0.05$; ****$p < 0.0001$; ns, not significant; *n* is given in parentheses; in (B), *n* gives the number of analyzed cells from three independent experiments.

A5 surface binding compared to wild-type ANO4 (Figures 7C, 7D, 7E, 7F, 7H, and 7J), suggesting higher rates of apoptosis. There was no significant difference in annexin A5 binding between mutants Asn603Asp, Val528Met, Asn129Lys, and Ile725Thr and wild-type ANO4 or GFP (Figures 7A, 7B, 7G, 7I, and 7J).

To evaluate $Ca^{2+}$-dependent scramblase activity, annexin A5 binding was further examined under the condition of increased intracellular $Ca^{2+}$. For this purpose, transfected cells were treated with 1 μM ionomycin for 10 min prior to measurement. There was no significant difference in annexin A5 surface binding between ANO4 wild-type and mutants compared to cells transfected with GFP only (Figures 8A–8J), indicating absence of $Ca^{2+}$-dependent scramblase activity of wild-type or mutant ANO4.

### Wild-type/mutant co-transfection experiments

In order to investigate a potential dominant-negative effect, we performed additional heterologous expression experiments in which we co-expressed both wild-type and mutant ANO4. For that purpose, we transfected HEK293 cells with a 50/50 combination of wild type with either the DEE-associated Asn558Lys or the GEFS+-associated Val528Met ANO4 mutant and analyzed the functional properties by patch-clamp analysis (Figures 9A, 9B, and 9E), surface expression of ANO4 proteins using immunocytochemistry (Figures 9C, 9D and 9G), early endosome co-localization (Figure S5), and annexin A5 surface expression (Figure S6). In contrast to transfection with mutants only, basal membrane conductance was significantly smaller in cells that had been transfected with wild-type/mutant combinations compared to wild type alone (Figure 9E). Similar to the mutants alone, the $Ca^{2+}$-induced cation conductance was significantly lower in cells with wild-type/mutant co-expression than that of wild-type ANO4 transfection. Regarding cell membrane surface expression, the co-transfected cells showed no difference between Val528Met and wild-type/Val528Met, whereas wild-type/Asn558Lys showed a small increase in ANO4 surface expression compared to Asn558Lys. We also observed small but significant differences in early endosome localization with wild-type/Asn558Lys showing increased and wild-type/Val528Met showing decreased levels of co-localization compared to mutant alone (Figure S5). However, given the small differences, we are cautious to consider these as biologically relevant. Scramblase activity measured by annexin A5 surface expression was similar in the co-transfection and single-transfection paradigm (Figure S6). In summary, we found evidence for a dominant-negative effect since the wild-type protein could not alleviate the deleterious effects of mutant ANO4 in co-expression experiments.

## Discussion

We identified *de novo* heterozygous missense variants in *ANO4* as a cause of fever-sensitive DEE/EE as well as autosomal dominantly inherited missense variants underlying familial GEFS+ and TLE with reduced penetrance.

All individuals of our cohort exhibiting *ANO4*-associated DEE/EE (I1–I5) first presented to the clinic with neurodevelopmental delay or epileptic seizures. Epilepsy was mostly of infantile onset, intractable, and included both generalized (tonic clonic, tonic, clonic, myoclonic, absences) and focal seizures. Fever-sensitivity was present in all. Interestingly, two recent genome-wide association studies have identified common variants near the *ANO3* (or *TMEM16C*) locus as a strong risk factor for febrile seizures.[78,79] Three individuals (I1–I3) displayed severe to profound impairment in psychomotor development from birth, whereas two individuals (I4–I5) showed initially normal development before regression to moderate to severe ID. They were therefore clinically classified as EE, but we acknowledge that it is challenging to distinguish a potential developmental impact of the underlying variant from detrimental effects of seizure activity on a vulnerable brain, and we assume that both likely contribute to the regression. All individuals display muscular hypotonia and non-specific anomalies in brain

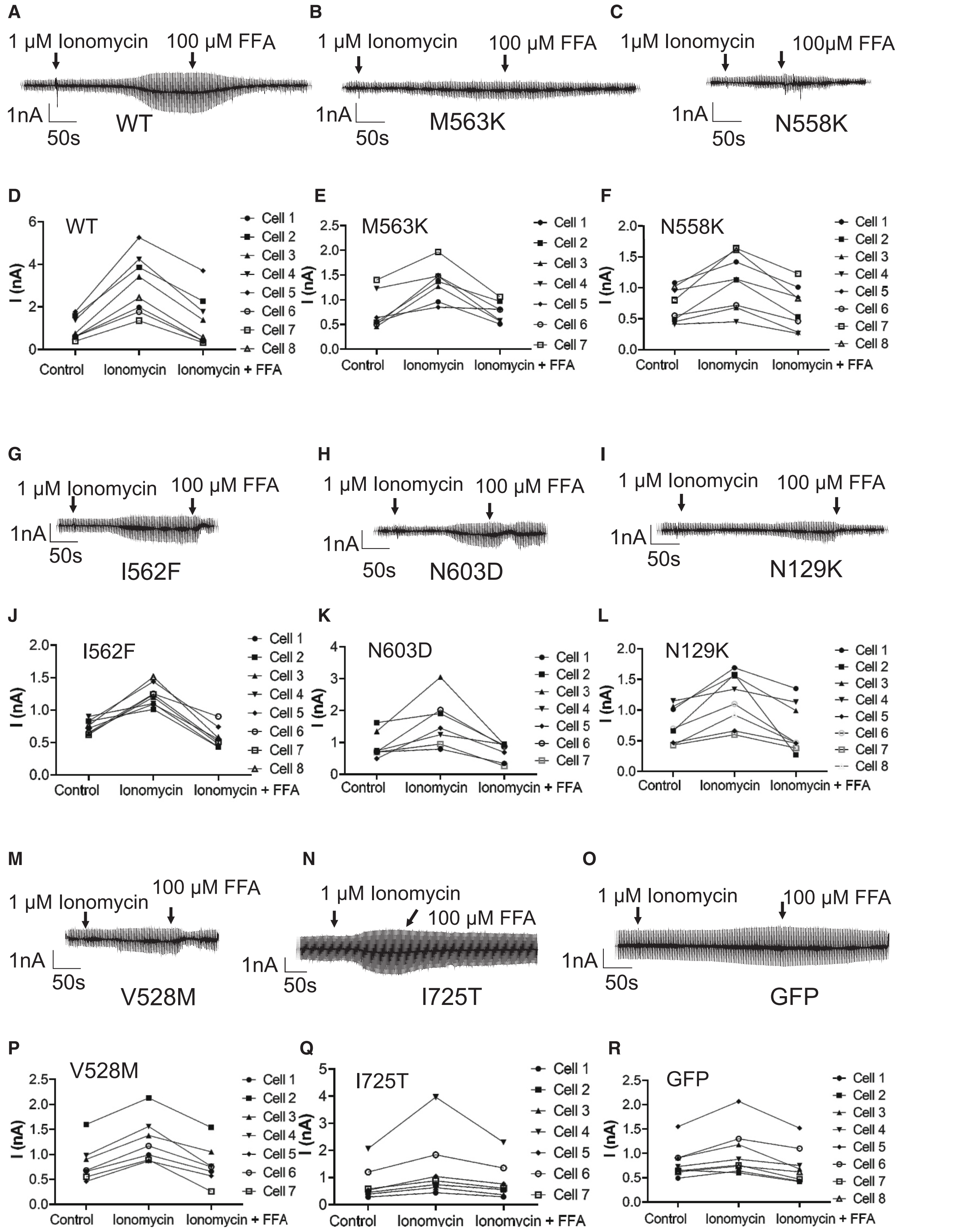


**Figure 5. The $Ca^{2+}$-dependent cation current elicited by heterologously expressed mutant ANO4 is blocked by the application of the flufenamic acid**

(A–C, G–I, and M–O) When ionomycin-mediated current density of HEK293 cells expressing wild-type or mutant ANO4 reached its peak, the flufenamic acid (100 μM) was applied to the bath solution (arrows). Raw current recordings of cells before and after the application of ionomycin and flufenamic acid (FFA) are shown. The HEK293 cells over-expressing wild-type ANO4 (A) showed increased current density after extracellular application of 1 μM ionomycin, which can be blocked by 100 μM FFA; the same result can be observed in

*(legend continued on next page)*

imaging. Other clinical features of this condition are variable and encompass secondary microcephaly and various neurological issues such as hyperkinesia or tremor. Additionally, some individuals exhibit feeding difficulties, eye anomalies (e.g., optic atrophy, strabismus, hypermetropia), severe scoliosis, hearing loss, sleep disturbances, hypothyroidism, and increased susceptibility to infections.

Additionally, we observed two inherited missense variants associated with non-syndromic GEFS+ in F6 or TLE in I7 with reduced penetrance and normal psychomotor development. F6 represents a large autosomal dominant GEFS+ pedigree in which we found the *ANO4* missense variant as the sole qualifying variant in the linked disease locus that was present in all 20 tested affected individuals and a disease penetrance of 73% (Figure S2). Of note, several members of F6 died of prolonged seizures or SUDEP, emphasizing that despite the normal psychomotor development, this is still a severe disorder. Strikingly, cortical dysplasia was detected on brain MRI in two members of this large family, as well as in I3. While cortical malformations have been previously reported in several ion channel disorders,[80] further investigation is necessary to assess their potential relationship to ANO4 dysfunction.

In contrast, the complete loss of one *ANO4* allele in an individual with autism and ID and the healthy father (DECIPHER ID 272667) does not cause any seizure phenotype. Furthermore, constraint metrics in gnomADv2.1.1 show some depletion of LoF variants (63.8 expected predicted loss-of-function [pLoF] variants vs. 19 observed; loss-of-function observed/expected upper bound fraction [LOEUF] = 0.44), but a probability of being LoF intolerant score (pLI) of 0 and no homozygous LoF variants. We therefore assume a variant-specific *ANO4* dysfunction rather than haploinsufficiency as pathomechanism in I1–I7 and hypothesize that haploinsufficiency of *ANO4* is not a cause of an apparent developmental disorder.

Functional analysis of cation conductance by whole-cell patch-clamp experiments demonstrated that all *ANO4* variants identified in our study cohort exhibited a significant reduction of $Ca^{2+}$-dependent cation conductance, with Asn558Lys, Ile562Phe, and Asn129Lys leading to a complete loss of ANO4-mediated cation conductance (Figures 3 and 4). The validity of this finding is supported by our earlier work, where we studied the putative cation selectivity of ANO4 by exchanging negatively charged conserved amino acids in the pore region with positively charged ones and found no LoF.[61] The sensitivity to inhibition by flufenamic acid supports that the measured currents indeed originate from anoctamin proteins (Figure 5). Immunostainings and co-localization analyses with a plasma membrane marker showed slightly reduced plasma membrane localization (Figures 3 and 4) and reduced early endosome localization of all five DEE/EE-associated ANO4 mutants (Figure 6). This finding indicates that reduced ANO4 membrane trafficking contributes to the loss of ion channel function in the *ANO4* variants associated with the severe encephalopathic phenotype (I1–I5). The two variants associated with the inherited epilepsy phenotypes (F6 and I7) did not show reduced plasma membrane localization. Of note, a considerable proportion of the wild-type as well as mutant protein appeared to be localized within the cytoplasm. Similar observations have been reported in previous studies and were interpreted to imply its intracellular function with relevance for $Ca^{2+}$ store filling processes.[81] However, we propose that the detected disease-associated variants primarily affect the ion channel pore function of ANO4 since ionic currents were reduced to 40% or less compared to the wild type, while the surface expression was normal or mildly reduced to 90% only (Figure 4).

Furthermore, we examined scramblase activity of ANO4 and its mutants by quantifying surface expression of annexin A5, which binds exposed PS with high affinity and is therefore used as a marker of apoptosis. ANO4 wild-type expression did not substantially change scramblase activity, neither in physiological nor in elevated $Ca^{2+}$ conditions, consistent with our previous findings.[61] However, all three DEE mutants (Asn558Lys, Ile562Phe, Met563Lys) displayed increased apoptosis at resting conditions (Figure 7) but not at increased $Ca^{2+}$-levels (Figure 8). These findings suggest another potential disease mechanism for the severe phenotype involving increased cell death due to mutant ANO4. This conclusion is supported by studies showing the role of apoptosis signaling in seizure-induced neuronal death and epileptogenesis[82,83] and is in line with the phenotype of DEE, where stagnation or worsening correlating with increased seizure activity is observed.

Finally, we studied a potential dominant-negative effect of ANO4 variants on the wild type using a co-transfection protocol in HEK293 cells. We selected one DEE and one GEFS+ variant with a strong LoF (Asn558Lys and Val528Met) and observed that in the presence of wildtype ANO4 the mutant variants Val528Met and Asn558Lys still suppressed $Ca^{2+}$-induced membrane conductance in a similar way as the mutants alone (Figure 9). Similarly, wild-type co-transfection did not alleviate the mutant effects on scramblase activity (as measured by the annexin A5 expression assays) but restored subcellular localization (Figures 9, S3 and S4). Moreover, a reduction in basal

the cells expressing ANO4 with the mutations Met563Lys (M563K; B), Asn558Lys (N558K; C), Ile562Phe (I562F; G), Asn603Asp (N603D; H), Asn129Lys (N129K; I), Val528Met (V528M; M), and Ile725Thr (I725T; N). GFP alone transfected cells in (O).
(D–F, J–L, and P–R) X axis indicates the baseline, presence of ionomycin and flufenamic acid; y axis shows the raw currents in nanoampere (nA). Effects of ionomycin and flufenamic acid (FFA) on each cell can be seen. The effect plots from HEK293 cells expressing ANO4 wild-type (D), ANO4 mutant Met563Lys (M563K; E), Asn558Lys (N558K; F), Ile562Phe (I562F; J), Asn603Asp (N603D; K), Asn129Lys (N129K; L), Val528Met (V528M; P), Ile725Thr (I725T; Q), and cells transfected with GFP alone (R).

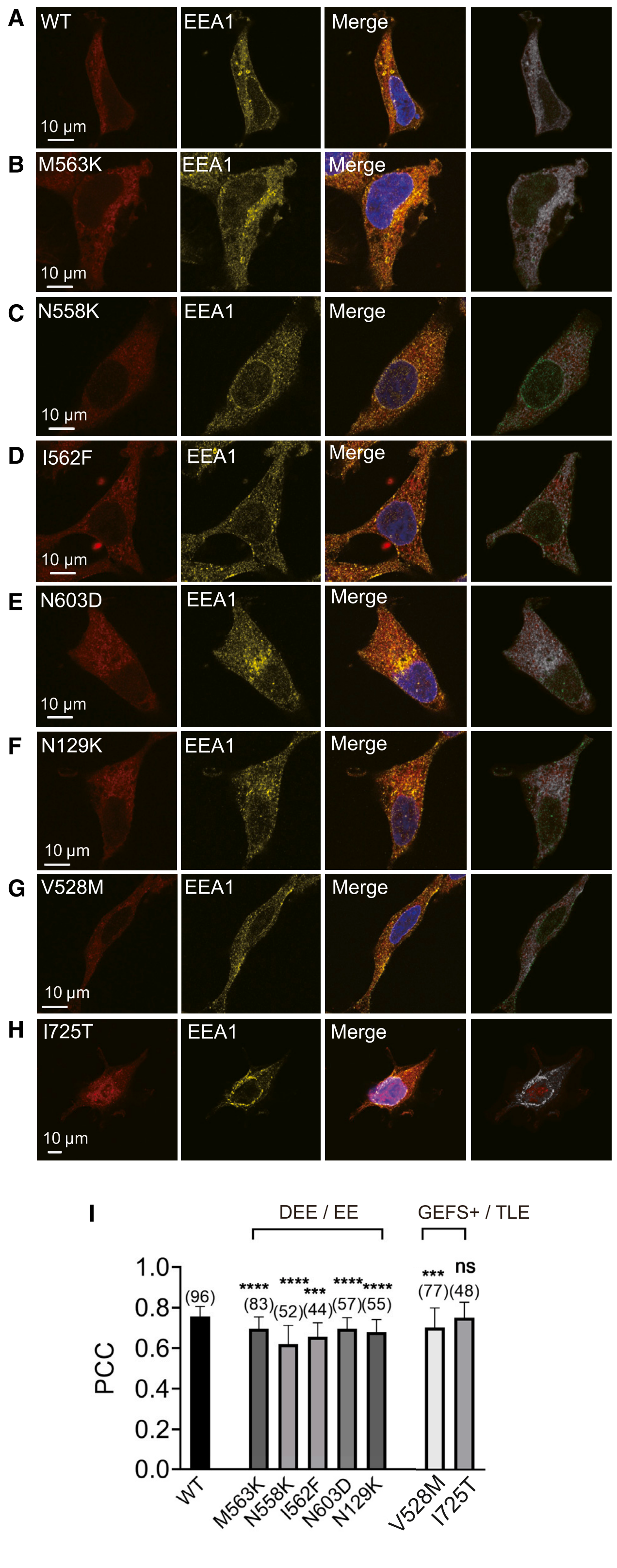


**Figure 6. Co-localization of wild-type and mutant ANO4 with the early endosome marker EEA1 in HEK293 cells**
(A–H) HEK293 cells expressing wild-type (A) and various mutated ANO4 constructs were stained with antibodies for ANO4 (left, red) and EEA1 (left-middle, yellow). Merged images (right-middle) show the co-localization of mutant ANO4 and EEA1. The gray dot images (right) represent the density of co-localization of mutant ANO4 and EEA1. Nuclei were stained with DAPI. ANO4 mutants included Met563Lys (M563K; B), Asn558Lys (N558K; C), Ile562Phe (I562F; D), Asn603Asp (N603D; E), Asn129Lys (N129K; F), Val528Met (V528M; G) and Ile725Thr (I725T; H). Scale bar represents 10 μm.
(I) PCC analysis of EEA1 and mutant ANO4 (transfection and immunostaining according to A–H). The number inside the parentheses represents n per group. Whiskers represent SEM. Values are given as mean ± SEM. Multiple comparisons were performed by ANOVA with Dunn's post hoc test. Data points that were statistical outliers were eliminated by the Grubbs outlier test. ***$p < 0.001$; ****$p < 0.0001$; ns, not significant; the number in parentheses represents the number of analyzed cells from three independent experiments.

membrane conductance for these two variants was only observed when co-transfecting wild-type and mutant, but not for mutants alone (Table 5). In summary, the co-transfection experiments suggest a strong dominant-negative effect of the mutants on wild-type ANO4.

Structural analysis revealed that all seven variants identified in the present study are localized within the globular domain of ANO4 with predicted destabilization of its structure (Figure 2). Concerning preliminary genotype-phenotype correlation, we noted that the two inherited variants associated with the less severe phenotypes of GEFS+ and TLE are the only variants that do not introduce new large side chains or changes in charge. Moreover, these are the only variants that did not show reduced plasma membrane location. Notably, both Val528Met and Ile725Thr are located in TMs that are not in proximity to the $Ca^{2+}$ binding site (TM3 and TM7, respectively). In contrast, all three variants occurring *de novo* in the most severe DEE phenotypes (Asn558Lys, Ile562Phe, and Met563Lys) cluster within transmembrane domain 4 close to the $Ca^{2+}$ binding site indicating that they not only disrupt the ANO4 structure but also likely impact $Ca^{2+}$ binding. The three DEE variants are also the only ones increasing ANO4 scramblase activity indicating increased apoptosis. Of the two *de novo* variants observed in EE, Asn603Asp detected in the child with the earlier onset of regression and more severe cognitive impairment is also located close to the $Ca^{2+}$ binding site but in a different TM (TM5, Figure 2A). Finally, the Asn129Lys variant observed in the individual with childhood-onset regression and moderate ID at teenage age is the only variant located in the N-terminal intracellular region within the predicted dimerization domain (PF16178).[67] In further support of this suggested genotype-phenotype correlation, four population variants with deleterious *in silico* predictions G115A, A535T, Y707C, and A728T, (dbSNP: rs34162417, rs150353677, rs143089752, and rs200450110, respectively)[84] are either rare and located in extracellular loops (A535T, Y707C, A728T) or very frequent (G115A, MAF 0.0476 with 390 homozygotes in gnomAD v2.1.1) and located in the N-terminal intracellular region but, in contrast to the EE-associated Asn129Lys variant, outside the dimerization domain and without introduction of a charged side chain (Figure 2A).

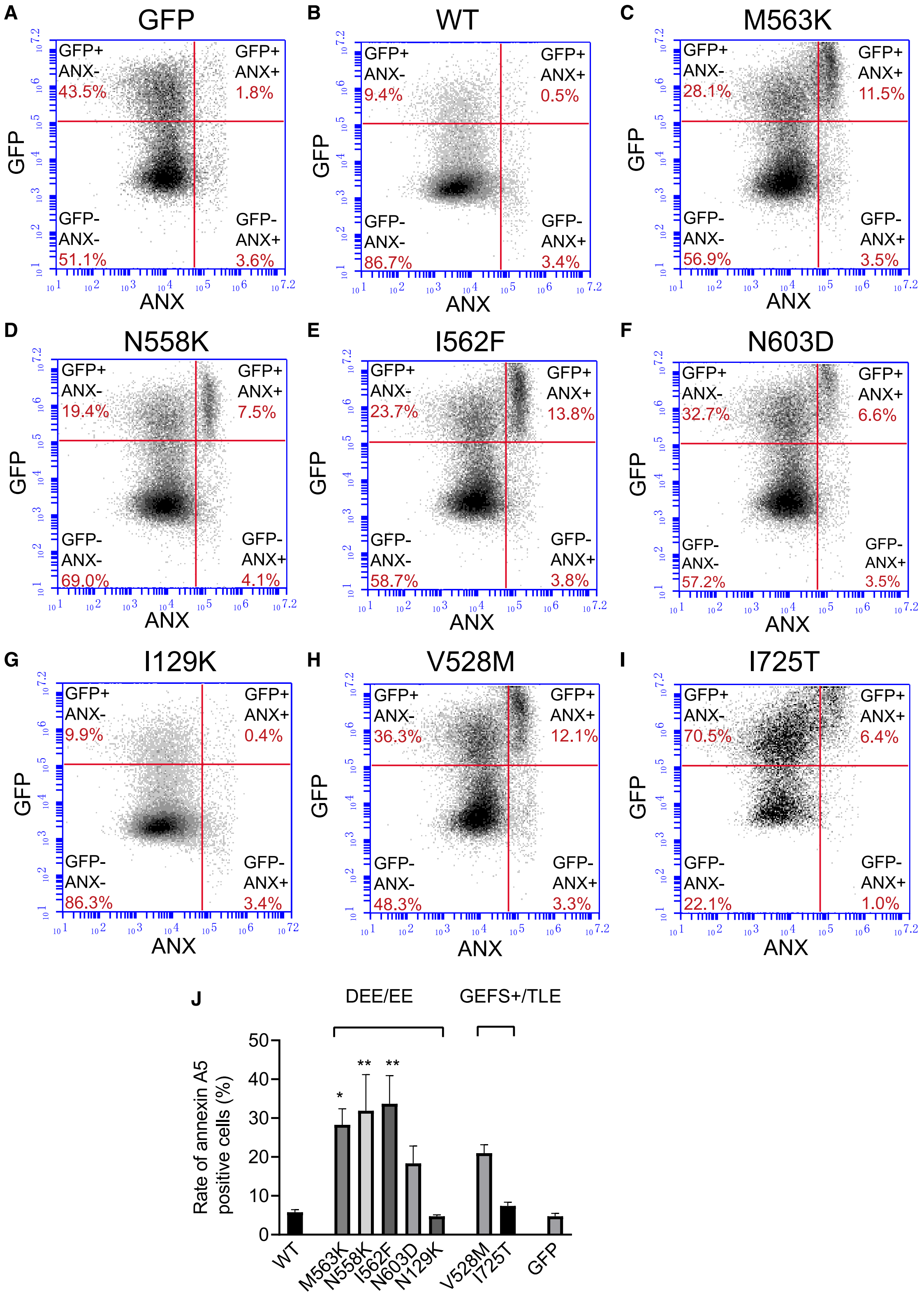


**Figure 7. Scramblase activity in HEK293 cells expressing wild-type or mutant ANO4 at physiological $Ca^{2+}$ levels**

(A–I) Scramblase activity was assessed by FACS sorting of annexin A5-labeled HEK293 cells that were not treated by ionomycin and transfected with GFP alone (A), with wildtype ANO4 plus GFP (B) and ANO4 mutants plus GFP under control conditions. ANO4 mutants included Met563Lys (M563K; C), Asn558Lys (N558K; D), Ile562Phe (I562F; E), Asn603Asp (N603D; F), Asn129Lys (N129K; G), Val528Met (V528M; H), and Ile725Thr (I725T; I). X axis indicates fluorescence intensity of Anx A5-6S-IDCC (log); y axis indicates fluorescence intensity of GFP (log). The right-upper square represents the ANO4 transfected, annexin A5-positive cell fraction.

(J) Comparison of annexin A5 surface expression between different transfection conditions. The experiments were carried out four times ($n = 4$). Values are given as mean ± SEM. Multiple comparisons were performed by ANOVA with Dunn's post hoc test. Data points that were statistical outliers were eliminated by the Grubbs outlier test. *$p < 0.05$; **$p < 0.01$.

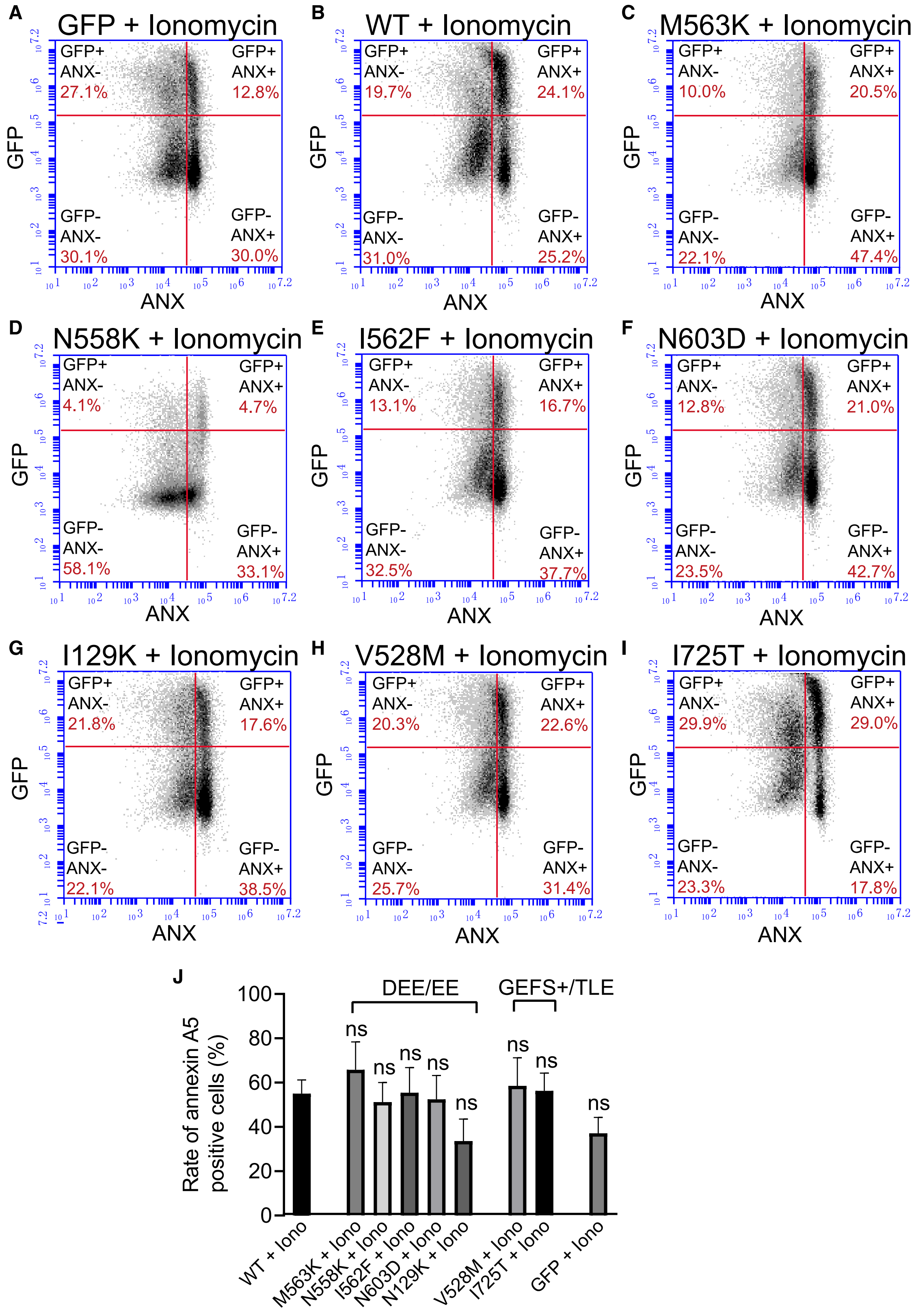


**Figure 8. $Ca^{2+}$-dependent scramblase activity in HEK293 cells expressing wild-type or mutant ANO4**

$Ca^{2+}$-dependent scramblase activity was assessed by FACS sorting of annexin A5-labeled HEK293 cells that were incubated with ionomycin (Iono, 1 μM) for 10 min and prior to the experiment transfected with GFP alone (A), with wild-type ANO4 plus GFP (B), and ANO4 mutations plus GFP: Met563Lys (M563K; C), Asn558Lys (N558K; D), Ile562Phe (I562F; E), Asn603Asp (N603D; F), Asn129Lys (N129K; G), Val528Met (V528M; H), and Ile725Thr (I725T; I). X axis indicates fluorescence intensity of annexin A5-6S-IDCC (log);

*(legend continued on next page)*

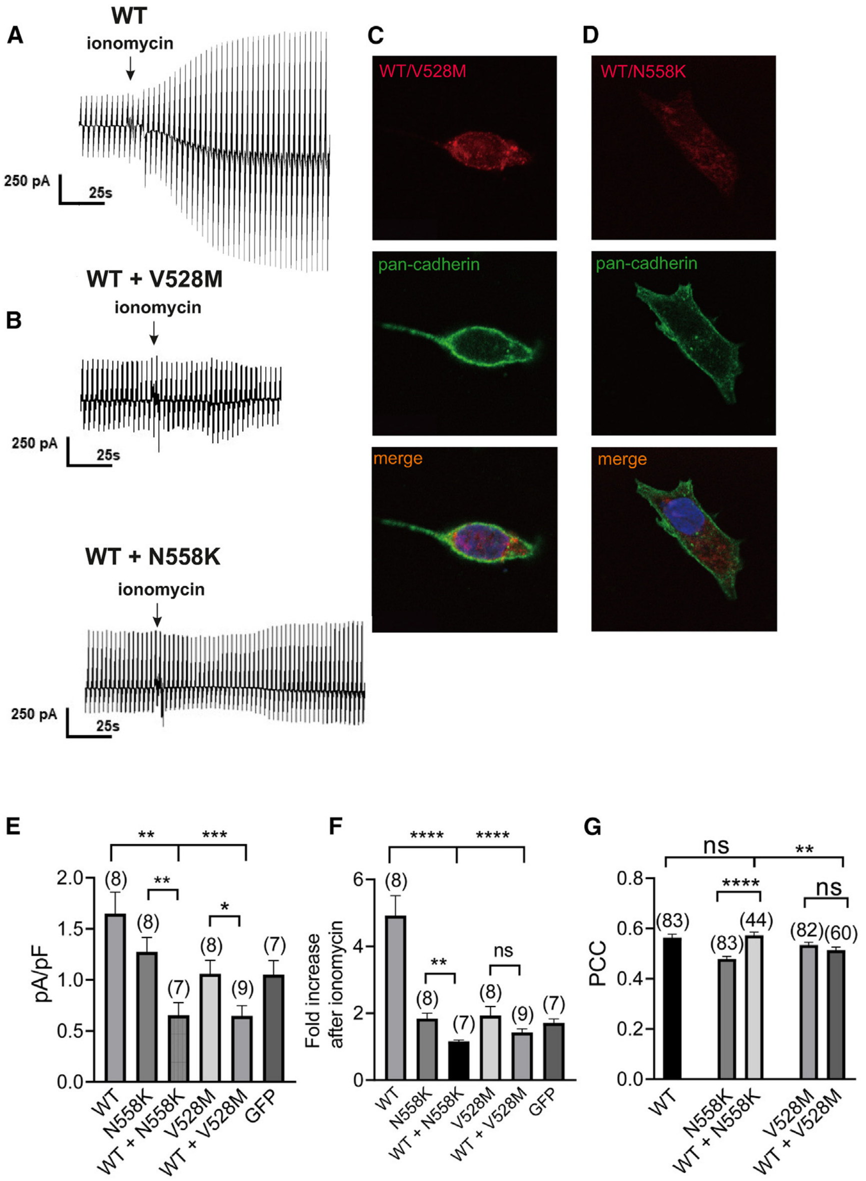


**Figure 9. ANO4 currents and ion channel surface expression in co-expression experiments**

HEK293 cells were co-transfected with either wild-type and DEE mutant Asn558Lys (N588K) or wild-type and GEFS+ mutant Val528Met (V528M) ANO4 and compared to wild-type or mutant transfection alone.

(A and B) (A) Raw current recording in HEK293 cells expressing wild-type ANO4 before and after application of ionomycin (1 μM; arrow) and (B) in wild-type/Val528-Met and wild-type/Asn558Lys co-expression.

(C and D) Immunostaining of a HEK293 cell co-expressing wild-type/Val528Met or wild-type/Asn558Lys against ANO4 (red) and the membrane marker pan-cadherin (green); from the top: ANO4 only, pan-cadherin only, merged ANO4 and pan-cadherin.

(E and F) (E) Comparison of peak current densities at physiological $Ca^{2+}$ conditions from cells with different transfection paradigm and (F) changes in membrane conductance after reaching a maximal steady-state level of the ionomycin effect; given as fold increase from baseline.

(G) Comparison of Pearson's correlation coefficient (PCC) of ANO4 and pan-cadherin-positive pixels from immunostainings of HEK293 cells expressing wild-type alone, mutant alone, or co-expressing wild-type/Val528Met or wild-type/Asn558Lys. Values are given as mean ± SEM. Multiple comparisons were performed by ANOVA with Dunn's post hoc test. Data points that were statistical outliers were eliminated by the Grubbs outlier test. *n* is given in parentheses. $^{*}p < 0.05$; $^{**}p < 0.01$; $^{***}p < 0.001$; $^{****}p < 0.0001$; ns, not significant.

In conclusion, this study identified missense variants in *ANO4* as a cause of fever-sensitive DEE/EE, GEFS+, and TLE and suggests possible pathophysiological mechanisms. The data suggest that the disease-associated variants act in a dominant-negative manner and lead to a decreased $Ca^{2+}$-dependent cation conductance due to reduced surface expression and impaired cation channel function of ANO4. Increased apoptosis rates observed for three ANO4 mutants may contribute to the disease mechanism. Additional studies are needed to further explore and support the pathomechanisms and genotype-phenotype correlation proposed here and unravel essential information about the localization of ANO4 and its functional role in neurons.

## Data and code availability

The accession numbers for the *ANO4* variants reported in this study are ClinVar: SCV004809186, SCV004809187, SCV004809188, SCV004809189, SCV004809190, SCV004809191, and SCV004812191. There are restrictions to the availability of further genomic data of the individuals studied here due to the consent given by them or their legal

y axis indicates fluorescence intensity of GFP (log). The right-upper square represents the ANO4 transfected, annexin A5-positive cell fraction.

(J) Comparison of annexin A5 surface expression between different transfection conditions. The experiments were carried out four times ($n = 4$). Values are given as mean ± SEM. Multiple comparisons were performed by ANOVA with Dunn's post hoc test. Data points that were statistical outliers were eliminated by the Grubbs outlier test. ns, not significant

guardians. All sharable genomic data are published in the supplement. Other data will be provided upon legitimate requests.

## Supplemental information

Supplemental information can be found online at https://doi.org/10.1016/j.ajhg.2024.04.014.


## Acknowledgments

The work was supported by the Swiss National Science Foundation (SNSF 320020_179547 to A.R.), the University Research Priority Program of the University of Zurich *Innovative Therapies in Rare Diseases* (ITINERARE to A.R.), the Clinical Research Priority Program of the University of Zurich Clarification of genetic variants for personalized prenatal and reproductive medicine (*praeclare* to A.R.), and by the Deutsche Forschungsgemeinschaft (DFG STR480/14-1 to O.S.). S.W. received support from FWO (1861424N and G056122N). This study makes use of data generated by the DECIPHER community. A full list of centers that contributed to the generation of the data is available from https://deciphergenomics.org/about/stats and via email from contact@deciphergenomics.org. Funding for the DECIPHER project was provided by Wellcome (grant number WT223718/Z/21/Z).


## Author contributions

F.Y.: investigation, formal analysis, writing – original draft preparation, writing – review & editing. A.B.: investigation, data curation, writing – original draft preparation, writing – review & editing. N.R.: resources, methodology, writing – review & editing. A.H.: methodology, writing – review & editing. K.S.: investigation, writing – review & editing. E.S.: supervision, methodology, writing – review & editing. R.F.S.: writing – review & editing. M.B.: investigation, writing – review & editing. S.B.: writing – original draft preparation, writing – review & editing. P.B.: investigation, writing – review & editing.T.C.: investigation, writing – review & editing. B.D.: investigation, writing – review & editing. B.G.: investigation, writing – review & editing. K.H.: investigation, writing – review & editing. M.J.: investigation, writing – review & editing. L.L.: investigation, writing – review & editing. T.L.: investigation, writing – review & editing. C.P.: investigation, writing – review & editing. N.S.: investigation, writing – review & editing. M.S.: investigation, writing – review & editing. S.P.T.: investigation, writing – review & editing. K.V.G.: investigation, writing – review & editing. W.V.P.: investigation, writing – review & editing. N.V.: investigation, writing – review & editing. A.Z.: investigation, writing – review & editing. M.Z.: Investigation, Writing – review & editing. A.H.C.H.: investigation, writing – review & editing. H.S.: investigation, supervision, writing – review & editing. H.L.: conceptualization, writing – review & editing. S.W.: investigation, methodology, supervision, writing – review & editing. O.S.: conceptualization, methodology, funding acquisition, supervision, project administration, writing – original draft preparation, writing – review & editing. A.R.: conceptualization, methodology, funding acquisition, supervision, project administration, writing – original draft preparation, writing – review & editing.

## Declaration of interests

K.H. is currently employed by Janssen Research & Development, Janssen Pharmaceutica N.V., Turnhoutseweg 30, Beerse B-2340, Belgium.


Received: November 17, 2023
Accepted: April 18, 2024
Published: May 13, 2024


## Web resources

AlphaFold Protein Structure Database, alphafold.ebi.ac.uk/entry/Q32M45
Biorender online software, biorender.com
Genome Aggregation Database (gnomAD), gnomad.broadinstitute.org
Human Brain Transcriptome database, hbatlas.org
Mouse Genome Database (MGD), informatics.jax.org
Protein Data Bank (PDB), rcsb.org/structure/6qp6
The Human Protein Atlas, proteinatlas.org/ENSG00000151572-ANO4

## References


1. Sheidley, B.R., Malinowski, J., Bergner, A.L., Bier, L., Gloss, D.S., Mu, W., Mulhern, M.M., Partack, E.J., and Poduri, A. (2022). Genetic testing for the epilepsies: A systematic review. Epilepsia *63*, 375–387. https://doi.org/10.1111/epi.17141.
2. Grether, A., Ivanovski, I., Russo, M., Begemann, A., Steindl, K., Abela, L., Papik, M., Zweier, M., Oneda, B., Joset, P., and Rauch, A. (2023). The current benefit of genome sequencing compared to exome sequencing in patients with developmental or epileptic encephalopathies. Mol. Genet. Genomic Med. *11*, e2148. https://doi.org/10.1002/mgg3.2148.
3. Musante, L., Costa, P., Zanus, C., Faletra, F., Murru, F.M., Bianco, A.M., La Bianca, M., Ragusa, G., Athanasakis, E., d'Adamo, A.P., et al. (2022). The Genetic Diagnosis of Ultrarare DEEs: An Ongoing Challenge. Genes *13*, 500. https://doi.org/10.3390/genes13030500.
4. Papuc, S.M., Abela, L., Steindl, K., Begemann, A., Simmons, T.L., Schmitt, B., Zweier, M., Oneda, B., Socher, E., Crowther, L.M., et al. (2019). The role of recessive inheritance in early-onset epileptic encephalopathies: a combined whole-exome sequencing and copy number study. Eur. J. Hum. Genet. *27*, 408–421. https://doi.org/10.1038/s41431-018-0299-8.
5. Brunner, J.D., Lim, N.K., Schenck, S., Duerst, A., and Dutzler, R. (2014). X-ray structure of a calcium-activated TMEM16 lipid scramblase. Nature *516*, 207–212. https://doi.org/10.1038/nature13984.
6. Brunner, J.D., Schenck, S., and Dutzler, R. (2016). Structural basis for phospholipid scrambling in the TMEM16 family. Curr. Opin. Struct. Biol. *39*, 61–70. https://doi.org/10.1016/j.sbi.2016.05.020.
7. Kunzelmann, K., Nilius, B., Owsianik, G., Schreiber, R., Ousingsawat, J., Sirianant, L., Wanitchakool, P., Bevers, E.M., and Heemskerk, J.W.M. (2014). Molecular functions of anoctamin 6 (TMEM16F): a chloride channel, cation channel, or phospholipid scramblase? Pflügers Archiv *466*, 407–414. https://doi.org/10.1007/s00424-013-1305-1.
8. Paulino, C., Neldner, Y., Lam, A.K., Kalienkova, V., Brunner, J.D., Schenck, S., and Dutzler, R. (2017). Structural basis for

anion conduction in the calcium-activated chloride channel TMEM16A. Elife *6*, e26232. https://doi.org/10.7554/eLife.26232.

9. Pedemonte, N., and Galietta, L.J.V. (2014). Structure and function of TMEM16 proteins (anoctamins). Physiol. Rev. *94*, 419–459. https://doi.org/10.1152/physrev.00039.2011.
10. Picollo, A., Malvezzi, M., and Accardi, A. (2015). TMEM16 proteins: unknown structure and confusing functions. J. Mol. Biol. *427*, 94–105. https://doi.org/10.1016/j.jmb.2014.09.028.
11. Falzone, M.E., Malvezzi, M., Lee, B.C., and Accardi, A. (2018). Known structures and unknown mechanisms of TMEM16 scramblases and channels. J. Gen. Physiol. *150*, 933–947. https://doi.org/10.1085/jgp.201711957.
12. Nagata, S., Suzuki, J., Segawa, K., and Fujii, T. (2016). Exposure of phosphatidylserine on the cell surface. Cell Death Differ. *23*, 952–961. https://doi.org/10.1038/cdd.2016.7.
13. Schreiber, R., Uliyakina, I., Kongsuphol, P., Warth, R., Mirza, M., Martins, J.R., and Kunzelmann, K. (2010). Expression and function of epithelial anoctamins. J. Biol. Chem. *285*, 7838–7845. https://doi.org/10.1074/jbc.M109.065367.
14. Tian, Y., Schreiber, R., and Kunzelmann, K. (2012). Anoctamins are a family of Ca2+-activated Cl- channels. J. Cell Sci. *125*, 4991–4998. https://doi.org/10.1242/jcs.109553.
15. Billig, G.M., Pál, B., Fidzinski, P., and Jentsch, T.J. (2011). Ca2+-activated Cl- currents are dispensable for olfaction. Nat. Neurosci. *14*, 763–769. https://doi.org/10.1038/nn.2821.
16. Caputo, A., Caci, E., Ferrera, L., Pedemonte, N., Barsanti, C., Sondo, E., Pfeffer, U., Ravazzolo, R., Zegarra-Moran, O., and Galietta, L.J.V. (2008). TMEM16A, a membrane protein associated with calcium-dependent chloride channel activity. Science *322*, 590–594. https://doi.org/10.1126/science.1163518.
17. Schroeder, B.C., Cheng, T., Jan, Y.N., and Jan, L.Y. (2008). Expression cloning of TMEM16A as a calcium-activated chloride channel subunit. Cell *134*, 1019–1029. https://doi.org/10.1016/j.cell.2008.09.003.
18. Stöhr, H., Heisig, J.B., Benz, P.M., Schöberl, S., Milenkovic, V.M., Strauss, O., Aartsen, W.M., Wijnholds, J., Weber, B.H.F., and Schulz, H.L. (2009). TMEM16B, a novel protein with calcium-dependent chloride channel activity, associates with a presynaptic protein complex in photoreceptor terminals. J. Neurosci. *29*, 6809–6818. https://doi.org/10.1523/JNEUROSCI.5546-08.2009.
19. Yang, Y.D., Cho, H., Koo, J.Y., Tak, M.H., Cho, Y., Shim, W.S., Park, S.P., Lee, J., Lee, B., Kim, B.M., et al. (2008). TMEM16A confers receptor-activated calcium-dependent chloride conductance. Nature *455*, 1210–1215. https://doi.org/10.1038/nature07313.
20. Schreiber, R., Faria, D., Skryabin, B.V., Wanitchakool, P., Rock, J.R., and Kunzelmann, K. (2015). Anoctamins support calcium-dependent chloride secretion by facilitating calcium signaling in adult mouse intestine. Pflügers Archiv *467*, 1203–1213. https://doi.org/10.1007/s00424-014-1559-2.
21. Suzuki, J., Fujii, T., Imao, T., Ishihara, K., Kuba, H., and Nagata, S. (2013). Calcium-dependent phospholipid scramblase activity of TMEM16 protein family members. J. Biol. Chem. *288*, 13305–13316. https://doi.org/10.1074/jbc.M113.457937.
22. Gyobu, S., Miyata, H., Ikawa, M., Yamazaki, D., Takeshima, H., Suzuki, J., and Nagata, S. (2016). A Role of TMEM16E Carrying a Scrambling Domain in Sperm Motility. Mol. Cell Biol. *36*, 645–659. https://doi.org/10.1128/MCB.00919-15.
23. Scudieri, P., Caci, E., Venturini, A., Sondo, E., Pianigiani, G., Marchetti, C., Ravazzolo, R., Pagani, F., and Galietta, L.J.V. (2015). Ion channel and lipid scramblase activity associated with expression of TMEM16F/ANO6 isoforms. J. Physiol. *593*, 3829–3848. https://doi.org/10.1113/JP270691.
24. Yang, H., Kim, A., David, T., Palmer, D., Jin, T., Tien, J., Huang, F., Cheng, T., Coughlin, S.R., Jan, Y.N., and Jan, L.Y. (2012). TMEM16F forms a Ca2+-activated cation channel required for lipid scrambling in platelets during blood coagulation. Cell *151*, 111–122. https://doi.org/10.1016/j.cell.2012.07.036.
25. Cenedese, V., Betto, G., Celsi, F., Cherian, O.L., Pifferi, S., and Menini, A. (2012). The voltage dependence of the TMEM16B/anoctamin2 calcium-activated chloride channel is modified by mutations in the first putative intracellular loop. J. Gen. Physiol. *139*, 285–294. https://doi.org/10.1085/jgp.201110764.
26. Cruz-Rangel, S., De Jesús-Pérez, J.J., Contreras-Vite, J.A., Pérez-Cornejo, P., Hartzell, H.C., and Arreola, J. (2015). Gating modes of calcium-activated chloride channels TMEM16A and TMEM16B. J. Physiol. *593*, 5283–5298. https://doi.org/10.1113/JP271256.
27. Ferrera, L., Caputo, A., Ubby, I., Bussani, E., Zegarra-Moran, O., Ravazzolo, R., Pagani, F., and Galietta, L.J.V. (2009). Regulation of TMEM16A chloride channel properties by alternative splicing. J. Biol. Chem. *284*, 33360–33368. https://doi.org/10.1074/jbc.M109.046607.
28. O'Driscoll, K.E., Pipe, R.A., and Britton, F.C. (2011). Increased complexity of Tmem16a/Anoctamin 1 transcript alternative splicing. BMC Mol. Biol. *12*, 35. https://doi.org/10.1186/1471-2199-12-35.
29. Xiao, Q., and Cui, Y. (2014). Acidic amino acids in the first intracellular loop contribute to voltage- and calcium- dependent gating of anoctamin1/TMEM16A. PLoS One *9*, e99376. https://doi.org/10.1371/journal.pone.0099376.
30. Xiao, Q., Yu, K., Perez-Cornejo, P., Cui, Y., Arreola, J., and Hartzell, H.C. (2011). Voltage- and calcium-dependent gating of TMEM16A/Ano1 chloride channels are physically coupled by the first intracellular loop*108*, pp. 8891–8896. https://doi.org/10.1073/pnas.1102147108.
31. Al Sharie, A.H., Abu Mousa, B.M., Al Zu'bi, Y.O., Al Qudah, M.A., Jaradat, S.A., Barakat, A., and Altamimi, E. (2023). A Novel ANO1 Gene Variant is Associated with Intestinal Dysmotility Syndrome Masquerading as Hirschsprung Disease: A Case Report. JPGN Rep. *4*, e317. https://doi.org/10.1097/PG9.0000000000000317.
32. Park, J.H., Ousingsawat, J., Cabrita, I., Bettels, R.E., Große-Onnebrink, J., Schmalstieg, C., Biskup, S., Reunert, J., Rust, S., Schreiber, R., et al. (2021). TMEM16A deficiency: a potentially fatal neonatal disease resulting from impaired chloride currents. J. Med. Genet. *58*, 247–253. https://doi.org/10.1136/jmedgenet-2020-106978.
33. Pinard, A., Ye, W., Fraser, S.M., Rosenfeld, J.A., Pichurin, P., Hickey, S.E., Guo, D., Cecchi, A.C., Boerio, M.L., Guey, S., et al. (2023). Rare variants in ANO1, encoding a calcium-activated chloride channel, predispose to moyamoya disease. Brain *146*, 3616–3623. https://doi.org/10.1093/brain/awad172.
34. Charlesworth, G., Plagnol, V., Holmström, K.M., Bras, J., Sheerin, U.M., Preza, E., Rubio-Agusti, I., Ryten, M., Schneider, S.A., Stamelou, M., et al. (2012). Mutations in ANO3 cause dominant craniocervical dystonia: ion channel implicated in pathogenesis. Am. J. Hum. Genet. *91*, 1041–1050. https://doi.org/10.1016/j.ajhg.2012.10.024.

35. Stamelou, M., Charlesworth, G., Cordivari, C., Schneider, S.A., Kägi, G., Sheerin, U.M., Rubio-Agusti, I., Batla, A., Houlden, H., Wood, N.W., and Bhatia, K.P. (2014). The phenotypic spectrum of DYT24 due to ANO3 mutations. Mov. Disord. *29*, 928–934. https://doi.org/10.1002/mds.25802.
36. Di Zanni, E., Gradogna, A., Scholz-Starke, J., and Boccaccio, A. (2018). Gain of function of TMEM16E/ANO5 scrambling activity caused by a mutation associated with gnathodiaphyseal dysplasia. Cell. Mol. Life Sci. *75*, 1657–1670. https://doi.org/10.1007/s00018-017-2704-9.
37. Griffin, D.A., Johnson, R.W., Whitlock, J.M., Pozsgai, E.R., Heller, K.N., Grose, W.E., Arnold, W.D., Sahenk, Z., Hartzell, H.C., and Rodino-Klapac, L.R. (2016). Defective membrane fusion and repair in Anoctamin5-deficient muscular dystrophy. Hum. Mol. Genet. *25*, 1900–1911. https://doi.org/10.1093/hmg/ddw063.
38. Marconi, C., Brunamonti Binello, P., Badiali, G., Caci, E., Cusano, R., Garibaldi, J., Pippucci, T., Merlini, A., Marchetti, C., Rhoden, K.J., et al. (2013). A novel missense mutation in ANO5/TMEM16E is causative for gnathodiaphyseal dyplasia in a large Italian pedigree. Eur. J. Hum. Genet. *21*, 613–619. https://doi.org/10.1038/ejhg.2012.224.
39. Mizuta, K., Tsutsumi, S., Inoue, H., Sakamoto, Y., Miyatake, K., Miyawaki, K., Noji, S., Kamata, N., and Itakura, M. (2007). Molecular characterization of GDD1/TMEM16E, the gene product responsible for autosomal dominant gnathodiaphyseal dysplasia. Biochem. Biophys. Res. Commun. *357*, 126–132. https://doi.org/10.1016/j.bbrc.2007.03.108.
40. Bolduc, V., Marlow, G., Boycott, K.M., Saleki, K., Inoue, H., Kroon, J., Itakura, M., Robitaille, Y., Parent, L., Baas, F., et al. (2010). Recessive mutations in the putative calcium-activated chloride channel Anoctamin 5 cause proximal LGMD2L and distal MMD3 muscular dystrophies. Am. J. Hum. Genet. *86*, 213–221. https://doi.org/10.1016/j.ajhg.2009.12.013.
41. Hicks, D., Sarkozy, A., Muelas, N., Köehler, K., Huebner, A., Hudson, G., Chinnery, P.F., Barresi, R., Eagle, M., Polvikoski, T., et al. (2011). A founder mutation in Anoctamin 5 is a major cause of limb-girdle muscular dystrophy. Brain *134*, 171–182. https://doi.org/10.1093/brain/awq294.
42. Liewluck, T., Winder, T.L., Dimberg, E.L., Crum, B.A., Heppelmann, C.J., Wang, Y., Bergen, H.R., 3rd, and Milone, M. (2013). ANO5-muscular dystrophy: clinical, pathological and molecular findings. Eur. J. Neurol. *20*, 1383–1389. https://doi.org/10.1111/ene.12191.
43. Milone, M., Liewluck, T., Winder, T.L., and Pianosi, P.T. (2012). Amyloidosis and exercise intolerance in ANO5 muscular dystrophy. Neuromuscul. Disord. *22*, 13–15. https://doi.org/10.1016/j.nmd.2011.07.005.
44. Penttila, S., Vihola, A., Palmio, J., and Udd, B. (1993). ANO5 Muscle Disease. In GeneReviews((R)), M.P. Adam, G.M. Mirzaa, R.A. Pagon, S.E. Wallace, L.J.H. Bean, K.W. Gripp, and A. Amemiya, eds.
45. Fujii, T., Sakata, A., Nishimura, S., Eto, K., and Nagata, S. (2015). TMEM16F is required for phosphatidylserine exposure and microparticle release in activated mouse platelets*112*, pp. 12800–12805. https://doi.org/10.1073/pnas.1516594112.
46. Balreira, A., Boczonadi, V., Barca, E., Pyle, A., Bansagi, B., Appleton, M., Graham, C., Hargreaves, I.P., Rasic, V.M., Lochmüller, H., et al. (2014). ANO10 mutations cause ataxia and coenzyme Q(1)(0) deficiency. J. Neurol. *261*, 2192–2198. https://doi.org/10.1007/s00415-014-7476-7.
47. Renaud, M., Anheim, M., Kamsteeg, E.J., Mallaret, M., Mochel, F., Vermeer, S., Drouot, N., Pouget, J., Redin, C., Salort-Campana, E., et al. (2014). Autosomal recessive cerebellar ataxia type 3 due to ANO10 mutations: delineation and genotype-phenotype correlation study. JAMA Neurol. *71*, 1305–1310. https://doi.org/10.1001/jamaneurol.2014.193.
48. Vermeer, S., Hoischen, A., Meijer, R.P.P., Gilissen, C., Neveling, K., Wieskamp, N., de Brouwer, A., Koenig, M., Anheim, M., Assoum, M., et al. (2010). Targeted next-generation sequencing of a 12.5 Mb homozygous region reveals ANO10 mutations in patients with autosomal-recessive cerebellar ataxia. Am. J. Hum. Genet. *87*, 813–819. https://doi.org/10.1016/j.ajhg.2010.10.015.
49. Jun, I., Park, H.S., Piao, H., Han, J.W., An, M.J., Yun, B.G., Zhang, X., Cha, Y.H., Shin, Y.K., Yook, J.I., et al. (2017). ANO9/TMEM16J promotes tumourigenesis via EGFR and is a novel therapeutic target for pancreatic cancer. Br. J. Cancer *117*, 1798–1809. https://doi.org/10.1038/bjc.2017.355.
50. Li, C., Cai, S., Wang, X., and Jiang, Z. (2015). Identification and characterization of ANO9 in stage II and III colorectal carcinoma. Oncotarget *6*, 29324–29334. https://doi.org/10.18632/oncotarget.4979.
51. Athanasiu, L., Mattingsdal, M., Kähler, A.K., Brown, A., Gustafsson, O., Agartz, I., Giegling, I., Muglia, P., Cichon, S., Rietschel, M., et al. (2010). Gene variants associated with schizophrenia in a Norwegian genome-wide study are replicated in a large European cohort. J. Psychiatr. Res. *44*, 748–753. https://doi.org/10.1016/j.jpsychires.2010.02.002.
52. Sherva, R., Tripodis, Y., Bennett, D.A., Chibnik, L.B., Crane, P.K., de Jager, P.L., Farrer, L.A., Saykin, A.J., Shulman, J.M., Naj, A., et al. (2014). Genome-wide association study of the rate of cognitive decline in Alzheimer's disease. Alzheimers Dement. *10*, 45–52. https://doi.org/10.1016/j.jalz.2013.01.008.
53. Terracciano, A., Sanna, S., Uda, M., Deiana, B., Usala, G., Busonero, F., Maschio, A., Scally, M., Patriciu, N., Chen, W.M., et al. (2010). Genome-wide association scan for five major dimensions of personality. Mol. Psychiatr. *15*, 647–656. https://doi.org/10.1038/mp.2008.113.
54. Webb, B.T., Guo, A.Y., Maher, B.S., Zhao, Z., van den Oord, E.J., Kendler, K.S., Riley, B.P., Gillespie, N.A., Prescott, C.A., Middeldorp, C.M., et al. (2012). Meta-analyses of genome-wide linkage scans of anxiety-related phenotypes. Eur. J. Hum. Genet. *20*, 1078–1084. https://doi.org/10.1038/ejhg.2012.47.
55. Sjöstedt, E., Zhong, W., Fagerberg, L., Karlsson, M., Mitsios, N., Adori, C., Oksvold, P., Edfors, F., Limiszewska, A., Hikmet, F., et al. (2020). An atlas of the protein-coding genes in the human, pig, and mouse brain. Science *367*, eaay5947. https://doi.org/10.1126/science.aay5947.
56. Kang, H.J., Kawasawa, Y.I., Cheng, F., Zhu, Y., Xu, X., Li, M., Sousa, A.M.M., Pletikos, M., Meyer, K.A., Sedmak, G., et al. (2011). Spatio-temporal transcriptome of the human brain. Nature *478*, 483–489. https://doi.org/10.1038/nature10523.
57. Maniero, C., Scudieri, P., Haris Shaikh, L., Zhao, W., Gurnell, M., Galietta, L.J.V., and Brown, M.J. (2019). ANO4 (Anoctamin 4) Is a Novel Marker of Zona Glomerulosa That Regulates Stimulated Aldosterone Secretion. Hypertension *74*, 1152–1159. https://doi.org/10.1161/HYPERTENSIONAHA.119.13287.
58. Maniero, C., Zhou, J., Shaikh, L.H., Azizan, E.A.B., McFarlane, I., Neogi, S., Scudieri, P., Galietta, L.J., and Brown, M.J. (2015). Role of ANO4 in regulation of aldosterone secretion in the zona glomerulosa of the human adrenal gland. Lancet *385*, S62. https://doi.org/10.1016/S0140-6736(15)60377-4.

59. Hendrickx, D.A.E., van Scheppingen, J., van der Poel, M., Bossers, K., Schuurman, K.G., van Eden, C.G., Hol, E.M., Hamann, J., and Huitinga, I. (2017). Gene Expression Profiling of Multiple Sclerosis Pathology Identifies Early Patterns of Demyelination Surrounding Chronic Active Lesions. Front. Immunol. *8*, 1810. https://doi.org/10.3389/fimmu.2017.01810.
60. Wittkowski, K.M., Dadurian, C., Seybold, M.P., Kim, H.S., Hoshino, A., and Lyden, D. (2018). Complex polymorphisms in endocytosis genes suggest alpha-cyclodextrin as a treatment for breast cancer. PLoS One *13*, e0199012. https://doi.org/10.1371/journal.pone.0199012.
61. Reichhart, N., Schöberl, S., Keckeis, S., Alfaar, A.S., Roubeix, C., Cordes, M., Crespo-Garcia, S., Haeckel, A., Kociok, N., Föckler, R., et al. (2019). Anoctamin-4 is a bona fide Ca(2+)-dependent non-selective cation channel. Sci. Rep. *9*, 2257. https://doi.org/10.1038/s41598-018-37287-y.
62. Tu, L., Bean, J.C., He, Y., Liu, H., Yu, M., Liu, H., Zhang, N., Yin, N., Han, J., Scarcelli, N.A., et al. (2023). Anoctamin 4 channel currents activate glucose-inhibited neurons in the mouse ventromedial hypothalamus during hypoglycemia. J. Clin. Invest. *133*, e163391. https://doi.org/10.1172/JCI163391.
63. Sobreira, N., Schiettecatte, F., Valle, D., and Hamosh, A. (2015). GeneMatcher: a matching tool for connecting investigators with an interest in the same gene. Hum. Mutat. *36*, 928–930. https://doi.org/10.1002/humu.22844.
64. Depondt, C., Van Paesschen, W., Matthijs, G., Legius, E., Martens, K., Demaerel, P., and Wilms, G. (2002). Familial temporal lobe epilepsy with febrile seizures. Neurology *58*, 1429–1433. https://doi.org/10.1212/wnl.58.9.1429.
65. Claes, L., Audenaert, D., Deprez, L., Van Paesschen, W., Depondt, C., Goossens, D., Del-Favero, J., Van Broeckhoven, C., and De Jonghe, P. (2004). Novel locus on chromosome 12q22-q23.3 responsible for familial temporal lobe epilepsy associated with febrile seizures. J. Med. Genet. *41*, 710–714. https://doi.org/10.1136/jmg.2004.019257.
66. Firth, H.V., Richards, S.M., Bevan, A.P., Clayton, S., Corpas, M., Rajan, D., Van Vooren, S., Moreau, Y., Pettett, R.M., and Carter, N.P. (2009). DECIPHER: Database of Chromosomal Imbalance and Phenotype in Humans Using Ensembl Resources. Am. J. Hum. Genet. *84*, 524–533. https://doi.org/10.1016/j.ajhg.2009.03.010.
67. Tien, J., Lee, H.Y., Minor, D.L., Jr., Jan, Y.N., and Jan, L.Y. (2013). Identification of a dimerization domain in the TMEM16A calcium-activated chloride channel (CaCC)*110*, pp. 6352–6357. https://doi.org/10.1073/pnas.1303672110.
68. Jumper, J., Evans, R., Pritzel, A., Green, T., Figurnov, M., Ronneberger, O., Tunyasuvunakool, K., Bates, R., Žídek, A., Potapenko, A., et al. (2021). Highly accurate protein structure prediction with AlphaFold. Nature *596*, 583–589. https://doi.org/10.1038/s41586-021-03819-2.
69. Varadi, M., Anyango, S., Deshpande, M., Nair, S., Natassia, C., Yordanova, G., Yuan, D., Stroe, O., Wood, G., Laydon, A., et al. (2022). AlphaFold Protein Structure Database: massively expanding the structural coverage of protein-sequence space with high-accuracy models. Nucleic Acids Res. *50*, D439–D444. https://doi.org/10.1093/nar/gkab1061.
70. Alvadia, C., Lim, N.K., Clerico Mosina, V., Oostergetel, G.T., Dutzler, R., and Paulino, C. (2019). Cryo-EM structures and functional characterization of the murine lipid scramblase TMEM16F. Elife *8*, e44365. https://doi.org/10.7554/eLife.44365.
71. Humphrey, W., Dalke, A., and Schulten, K. (1996). VMD: visual molecular dynamics. J. Mol. Graph. *14*, 33-28. https://doi.org/10.1016/0263-7855(96)00018-5.
72. Sievers, F., Wilm, A., Dineen, D., Gibson, T.J., Karplus, K., Li, W., Lopez, R., McWilliam, H., Remmert, M., Söding, J., et al. (2011). Fast, scalable generation of high-quality protein multiple sequence alignments using Clustal Omega. Mol. Syst. Biol. *7*, 539. https://doi.org/10.1038/msb.2011.75.
73. Haeckel, A., Appler, F., Figge, L., Kratz, H., Lukas, M., Michel, R., Schnorr, J., Zille, M., Hamm, B., and Schellenberger, E. (2014). XTEN-annexin A5: XTEN allows complete expression of long-circulating protein-based imaging probes as recombinant alternative to PEGylation. J. Nucl. Med. *55*, 508–514. https://doi.org/10.2967/jnumed.113.128108.
74. Sim, N.L., Kumar, P., Hu, J., Henikoff, S., Schneider, G., and Ng, P.C. (2012). SIFT web server: predicting effects of amino acid substitutions on proteins. Nucleic Acids Res. *40*, W452–W457. https://doi.org/10.1093/nar/gks539.
75. Schwarz, J.M., Cooper, D.N., Schuelke, M., and Seelow, D. (2014). MutationTaster2: mutation prediction for the deep-sequencing age. Nat. Methods *11*, 361–362. https://doi.org/10.1038/nmeth.2890.
76. Adzhubei, I.A., Schmidt, S., Peshkin, L., Ramensky, V.E., Gerasimova, A., Bork, P., Kondrashov, A.S., and Sunyaev, S.R. (2010). A method and server for predicting damaging missense mutations. Nat. Methods *7*, 248–249. https://doi.org/10.1038/nmeth0410-248.
77. Cheng, J., Novati, G., Pan, J., Bycroft, C., Žemgulytė, A., Applebaum, T., Pritzel, A., Wong, L.H., Zielinski, M., Sargeant, T., et al. (2023). Accurate proteome-wide missense variant effect prediction with AlphaMissense. Science *381*, eadg7492. https://doi.org/10.1126/science.adg7492.
78. Feenstra, B., Pasternak, B., Geller, F., Carstensen, L., Wang, T., Huang, F., Eitson, J.L., Hollegaard, M.V., Svanström, H., Vestergaard, M., et al. (2014). Common variants associated with general and MMR vaccine-related febrile seizures. Nat. Genet. *46*, 1274–1282. https://doi.org/10.1038/ng.3129.
79. Skotte, L., Fadista, J., Bybjerg-Grauholm, J., Appadurai, V., Hildebrand, M.S., Hansen, T.F., Banasik, K., Grove, J., Albiñana, C., Geller, F., et al. (2022). Genome-wide association study of febrile seizures implicates fever response and neuronal excitability genes. Brain *145*, 555–568. https://doi.org/10.1093/brain/awab260.
80. Smith, R.S., and Walsh, C.A. (2020). Ion Channel Functions in Early Brain Development. Trends Neurosci. *43*, 103–114. https://doi.org/10.1016/j.tins.2019.12.004.
81. Cabrita, I., Benedetto, R., Fonseca, A., Wanitchakool, P., Sirianant, L., Skryabin, B.V., Schenk, L.K., Pavenstädt, H., Schreiber, R., and Kunzelmann, K. (2017). Differential effects of anoctamins on intracellular calcium signals. Faseb. J. *31*, 2123–2134. https://doi.org/10.1096/fj.201600797RR.
82. Henshall, D.C. (2007). Apoptosis signalling pathways in seizure-induced neuronal death and epilepsy. Biochem. Soc. Trans. *35*, 421–423. https://doi.org/10.1042/BST0350421.
83. Henshall, D.C., and Simon, R.P. (2005). Epilepsy and apoptosis pathways. J. Cerebr. Blood Flow Metabol. *25*, 1557–1572. https://doi.org/10.1038/sj.jcbfm.9600149.
84. Reichhart, N., Milenkovic, V.M., Wetzel, C.H., and Strauß, O. (2021). Prediction of Functional Consequences of Missense Mutations in ANO4 Gene. Int. J. Mol. Sci. *22*, 2732. https://doi.org/10.3390/ijms22052732.

The American Journal of Human Genetics, Volume *111*

## Supplemental information

# Missense variants in *ANO4* cause sporadic encephalopathic or familial epilepsy with evidence for a dominant-negative effect

**Fang Yang, Anais Begemann, Nadine Reichhart, Akvile Haeckel, Katharina Steindl, Eyk Schellenberger, Ronja Fini Sturm, Magalie Barth, Sissy Bassani, Paranchai Boonsawat, Thomas Courtin, Bruno Delobel, EuroEPINOMICS-RES Dravet working group, Boudewijn Gunning, Katia Hardies, Mélanie Jennesson, Louis Legoff, Tarja Linnankivi, Clément Prouteau, Noor Smal, Marta Spodenkiewicz, Sandra P. Toelle, Koen Van Gassen, Wim Van Paesschen, Nienke Verbeek, Alban Ziegler, Markus Zweier, Anselm H.C. Horn, Heinrich Sticht, Holger Lerche, Sarah Weckhuysen, Olaf Strauß, and Anita Rauch**

**Supplemental Note: Case Reports**

**Individual 1**

Individual 1 was born to healthy non-consanguinous parents after an uneventful pregnancy. He was a floppy child and developed epilepsy at age 2m. The initial seizure type was multifocal clonic with cloni of eyebrows, arms, tongue and unresponsiveness. Initial EEG showed rare sharp waves median frontal and central region as well in the right centroparietal region.

During disease course, observed seizure semiology was an initial cry, stiff legs then cloni of arms and cyanosis, occurring often during sleep, about 1x/week. Another documented seizure type was shorter hypermotoric seizures with multifocal cloni occurring about 1-2x/week, sometimes in clusters. Multiple EEG's at follow ups showed multifocal sharp waves, polyspike waves, and abnormal background activity. Seizures were fever sensitive. Until the age of 4-5 years, he had a strong susceptibility to infections, which improved afterwards.

Individual 1 was first referred for genetic evaluation at age 12 months due to severe muscular hypotonia, developmental delay and epilepsy, but no diagnosis could be established. We saw the child again at the age of 2y 11m. Growth parameters were weight 12.8 kg (P50-75), length 100 cm (P90) and head circumference 48.8 cm (P3-10). He showed pectus excavatum and finger pads as minor morphologic features. He was able to grip better and turn from the supine to the prone position on his own, but continued to have marked hypotonia. He was not able to sit or eat independently. He did not speak and pointed to things he wanted. He had at that moment a seizure-free period on antiseizure treatment with Levetiracetam. EEG did not show epileptic discharges. Sequencing of *POLG* and chromosomal microarray analysis and metabolic workup were unremarkable. He was included in a study of genetic causes of epileptic encephalopathy and a *de novo* missense variant in *ANO4* NM_001286615.1:c.1688T>A p.(Met563Lys) was detected. Additionally, he had an incidental finding of compound-heterozygous variants in *GJB2* NM_004004.5:c.[35del];[101T>C] potentially associated with late-onset hearing loss.

At last investigation at age 12 2/12 years, he showed a content demeanour. Head circumference was 52.1 cm (P3-10, z -1.75). He could neither move nor speak. He had a hyperkinetic movement disorder with ataxia and myoclonia, a pectus excavatum, and a mild flexion contracture of the right knee. Some

but not all of the myoclonia had a correlation in the EEG. He had several EEG's without any epileptic activity despite observed pathologic movements of the limbs. Myoclonia were therefore interpreted as being due to a movement disorder with myoclonia rather than myoclonic epilepsy. The parents reported that he had generalized tonic-clonic seizures with head rotation to the side, cyanosis, drooling and then cloni of the arms, occurring on average 2 times a week, and more frequently during teething and infections. Furthermore, he had reflux with occasional vomiting. While selective eating habits were present in early childhood, he now ate with appetite and ate almost everything in small pieces. He received a laxative because of mild constipation. He had hippotherapy which led to significant improvement of his truncal muscle tone, so that he could now be transferred to the toilet chair. He had hypermetropia and a strabism corrected with glasses. His hearing was not tested formally but was good in daily life, e.g. he responded to whispering. He had a pronounced need for sleep with a night sleep of about 14 hours, and difficulties to wake up in the morning. A detailed endocrinological examination was unremarkable except for  central hypothyroidism.

**Individual 2**

Individual 2 was the second boy from non-consanguineous parents and was born at 41 gestational weeks by primary C-section after an uneventful pregnancy. Growth parameters at birth were: weight 3310g, length 49.5 cm and OFC 36cm. APGAR score 10/10/10. In the neonatal period, axial hypotonia and peripheral hypertonia, sporadic rolling movements of the limbs, lack of eye contact, and feeding difficulties were noted. Seizure onset was at age 1 month. Brain MRI at that time was normal. Reported seizure types included generalized tonic clonic and focal impaired awareness. Seizures were therapy resistant typically occuring multiple times per week, and repeatedly excacerbated during infections. Antiepileptic treatments employed over the years included Phenobarbital, Valproate, Carbamazepine, Levetiracetam, Clonazepam, Phenytoin, and Topiramate. Ketogenic diet was given from age 1y 6m until 4y 4m when considered uneffective. At age 9y the child was reported to be seizure free during 1.5 years. At last follow up, antiepileptic treatment consisted of Levetiracetam, Clobazam, and Valproate.

Development was severely delayed from birth. Eye contact and babbling was noted at age 1y 6m. The child never acquired sitting and remained non-verbal.

Brain MRI at age 1y 2m showed severely delayed myelinisation, and follow up at age 2y 7m showed a cortico-subcortical atrophy with some white matter anomalies and cerebellar atrophy. The child suffered from severe spasticity and dystonia, treated with Hydrocortisone, Baclofen, Levodopa / Benserazide, and Clonidine. Associated pain was treated with Morphine, 5-Hydroxytryptophan and Amitriptyline. At age 6.5 months chronic vomiting and feeding difficulties led to gastrostomy. Vomiting episodes continued and could not be controlled by various medications. At age 1y 2m, failure to thrive was still a problem. Exacerbation of spasms and dystonia were reported during vomiting episodes.

At age 3y, optic atrophy and severe kyphosis were first reported. The child developed very severe scoliosis at the T5-T11 levels resulting in ventilation troubles i.e. a hypercapnia requiring oxygen therapy. Sacrum angulation S3-S4 is at 90°. At the pelvic level, the left hip is dislocated with a flexion of 60° and the right hip flexion is completely abolished. Furthermore, the child has bilateral knee contractures and feet deformation. At age 9y diffuse osteoporosis was diagnosed after a pathological femoral shaft fracture.

Trio exome sequencing analysis showed a *de novo* heterozygous NM_001286615.1:c.1674C>A p.(Asn558Lys) variant in *ANO4*.

The child deceased at age 12y 6m due to neurological and respiratory decompensation.

**Individual 4**

This 22-year-old male is the second child of healthy non consanguineous parents. He was born at term and his early developmental milestones were normal.

At the age of 11 months he had the first cluster of tonic/tonic- clonic seizures during a febrile infection. Afebrile seizures followed and focal impaired awareness seizures and myoclonias were observed from age 16 months. Seizures were triggered by fever, heat, infection and later by physical exercise. His psychomotor development was normal until 1,5 years of age, then slowed and stagnated during the third year of life, speech production being the most affected area.

EEG was normal at seizure onset. From 1,5 years of age, slowing of background activity and scarse interictal epileptiform findings, including frontocentral spikes independently form both hemispheres and generalized spike- and polyspike-and-wave discharges during sleep, were observed. At 3 and 9 years of

age, several seizures were caught in a video-EEG; 1. Focal impaired awareness seizures, evolving to right or left sided motor seizures. 2. sequential seizures starting with impaired awareness, followed by right-sided tonic-clonic and then generalized tonic-clonic and finally left sided motor seizure. EEG discharge was first non- lateralizing —then evolved to left hemisphere — generalized and finally shifted to right hemisphere. Myoclonia were associated with generalized polyspike-and-wave discharges and arousals from sleep with right centroparietal discharges. A diagnosis of Dravet-like syndrome was given, but no pathogenic variant of *SCN1A* was found.

During childhood, his seizure frequency varied from multiple per week -with clustering- to seizure freedom up to one year despite multiple anti- seizures medications (Table 2). Sodium channel blockers were not effective and were associated with gait instability. He has been seizure- free since age 17 years, when bromide was added to valproate, topiramate and clobazam. Presently, he continues with valproate and topiramate.

His psychomotor development was normal until 1,5 years of age, then slowed and stagnated during the third year of life. Speak production even regressed and he used signs and gestures to communicate. He learned to run and jump but had muscular hypotonia and balance problems. In a neuropsychological examination at the age of 5 years he performed at the level of mild intellectual disability and by 12 years of age he had declined cognitively to the level of moderate intellectual disability. According to parents, his skills have further regressed during early adulthood years; speech production is only a few words, speech reception is impaired, and he is uncapable of assembling puzzles, which previously used to be the best skill level.

Trio exome sequencing analysis showed a *de novo* heterozygous NM_001286615.1:c.1807A>G p.(Asn603Asp) variant in *ANO4*.

**Individual 5**

This 20-year-old male is the first child of healthy non-consanguineous parents. He was born at 36+3 gestational weeks. His early development was normal. At the age of 3,5 years, he had a febrile seizure. Soon other non-fever sensitive seizure types occurred, including tonic-clonic seizures (with afterwards shaking of his left hand), tonic seizures, focal seizures with impaired awareness and twitching in his

face, and myoclonia. He has frequent nocturnal seizures. His epilepsy was therapy resistant and classified as probably (multi)focal.

Antiepileptic treatment initially consisted of Valproate, Rufinamide and Clobazam. Ketogenic diet was introduced at age 5y and initially had a beneficial effect on the seizures, until about age 7y when it was no longer effective. At age 8y he received Valproate, Phenytoin, Lacosamide, and Clobazam. He started with vagal nerve stimulation which was effective to stop generalized tonic clonic seizures. After stopping Phenytoin his locomotion improved. Levetiracetam, Topiramate, and Zonisamide had had little effect on his seizures. At age 11y he received ketogenic diet again, making him more alert but without effect on the seizures. At age 16y Rufinamide was added, improving the generalized tonic clonic seizures, so that Lacosamide could be stopped. At last follow up at age 23y he was treated with Valproate, Rufinamide and Clobazam in addition to the vagus nerve stimulation.

MRI scans of his brain showed stable periventricular white matter anomalies. An MRI scan of the brain at age 16 years showed progressive cerebral atrophy.

Until age 4 years his cognitive development was normal. At age 4y 9m he scored an IQ of 100 in formal assessment. At age 7y he no longer benefited from ketogenic diet, and with many more seizures there was a cognitive decline at the age of 8y 1m (verbal IQ of 78 and performance IQ of 72 on WISC-III). At age 8y 6m he was diagnosed with epileptic encephalopathy. At age 14y he was diagnosed with autism spectrum disorder. At age 14 and 16 years his IQ score was 51.

He had low energy level. He used CPAP during the night due to sleep apnea. He had a normal height (186 cm, +0.4 SD) with a relatively large arm span (192 cm; ratio to height 1,03) and a relatively large head circumference (60.5 cm, +1.9 SD). He had (familial) hypertelorism and showed striae around the lower back and armpits.

Genetic analysis showed a *de novo* heterozygous NM_001286615.1:c.387C>G p.(Asn129Lys) variant in *ANO4*.

**Individual 7**

Individual 7 is a 39-year-old female from a non-consanguineous family. She was known to have a coarcatio aorta and was followed since the age of 8 years at the child neurology department because of

gait difficulties. These were caused by a dopa responsive dystonia due to a heterozygous deletion of the GTP cyclohydrolase I gene, and were controlled with a low dose of levodopa. Neurodevelopment was further normal. From the age of 18 years, she had recurrent short episodes that started with a déjà-vu or intrusive thoughts, followed by staring. Afterwards there was mild confusion, tiredness, and headache. By the age of 23 years, these episodes occurred up to 6 times a month. MRI of the brain showed an unspecific white matter lesion in the right centrum semiovale. EEG and 24-hours EEG were normal. A clinical diagnosis of temporal lobe epilepsy was made, and treatment with carbamazepine was started, after which the episodes disappeared. Carbamazepine was switched to levetiracetam since she planned a pregnancy. She discontinued levetiracetam at the age of 27 and has remained seizure free since. The brother of her maternal grandmother had died at the age of 16 years of severe epilepsy.

Individual 7 had an inconspicuous epilepsy panel analysis and was included in a cohort of individuals with febrile seizures and/or temporal lobe epilepsy for a *ANO4* candidate gene screening, which revealed the rare missense variant NM_001286615.1: c.2174T>C p.(Ile725Thr). The variant was inherited from her asymptomatic mother. DNA of other family members was not available for further segregation analysis.

**Supplemental Figures**

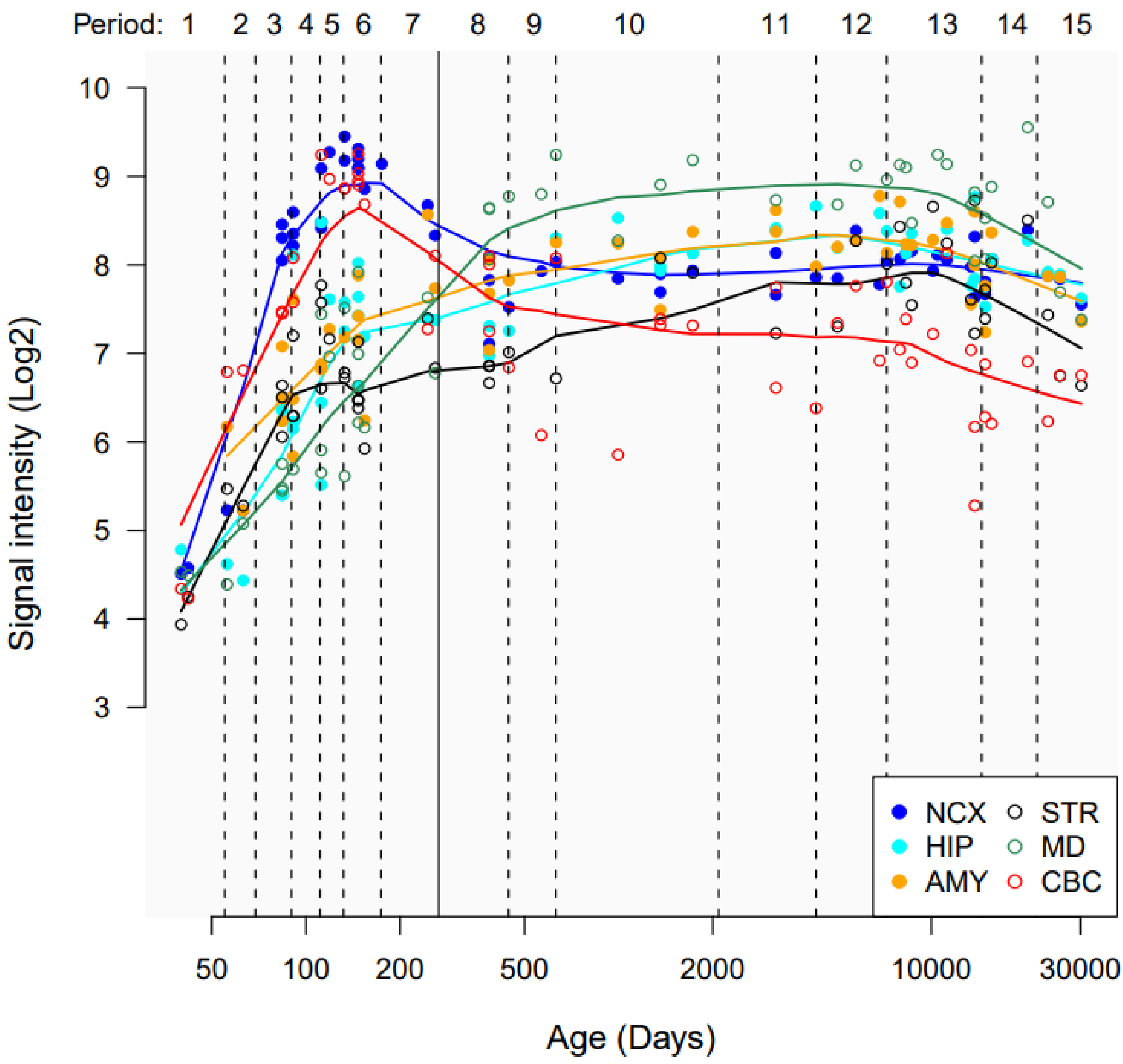


**Figure S1. *ANO4* mRNA expression in humans retrieved from the human brain transcriptome database.** Levels of human *ANO4* mRNA are depicted in different brain regions during pre- and postnatal periods.

Brain regions are abbreviated as follows: NCX = neocortex, HIP = Hippocampus, AMY = Amygdala, STR = Striatum, MD = Mediodorsal nucleus of the thalamus, CBC = Cerebellar cortex. On the top X axis, periods 1-7 denote embryonic and fetal development, specifically period 1 from post-conceptional weeks (PCW) 4 to 8, period 2 from PCW 8 to 10, period 3 from PCW 10 to 13, period 4 from PCW 13 to 16, period 5 from PCW 16 to 19, period 6 from PCW 19 to 24, and period 7 from PCW 24 to 38. Period 8 denotes neonatal and early infancy until age 6 months, period 9 refers to late infancy from age

6 months to 12 months, period 10 refers to early childhood from age 1 to 6 years, period 11 represents middle and late childhood from age 6 to 12 years, period 12 denotes adolescence from age 12 to 20 years, period 13 refers to young adulthood from age 20 to 40 years, period 14 represents middle adulthood from age 40 to 60 years, and period 15 denotes late adulthood from age 60 years onwards. Figure retrieved from the Human Brain Transcriptome database[1] at hbatlas.org on January 17 2024.

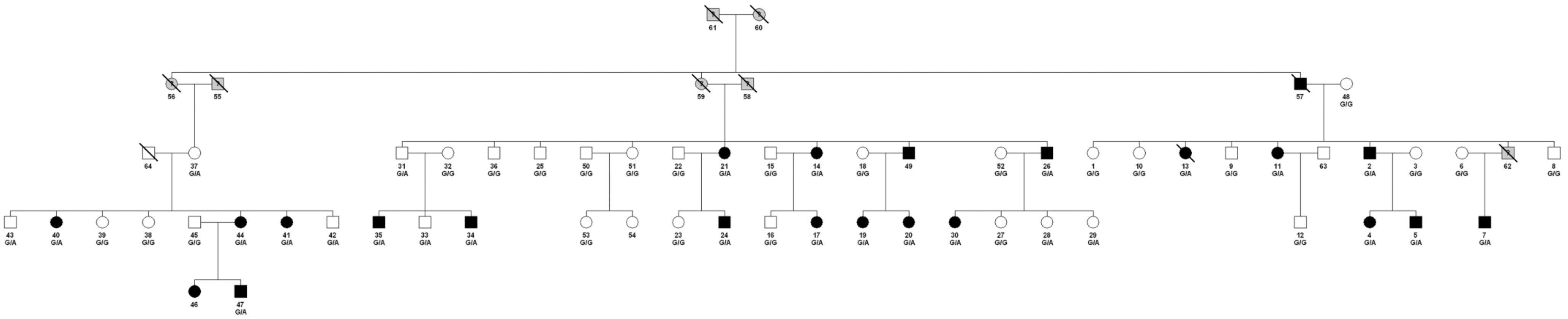

**Figure S2. Pedigree of Family 6.** Pedigree of members of family 6 that were available for linkage analysis and subsequent *ANO4* variant testing showing 23 individuals affected by GEFS+ (black filled symbols) with an autosomal-dominant inheritance pattern. Clinical information on the extemded family was previously published in Depondt et al. 2002[2]. Grey filled symbols with question mark denote unknown phenotype. If DNA was available for analysis the genotype is given below the individual (G/G wildtype, G/A heterozygous for the *ANO4* variant NM_001286615.1:c.1582G>A). The identified segregating *ANO4* variant was present in all 20 affected family members for whom DNA was available and had an 73% penetrance (22 affected [obligate] carriers/30 total [obligate] carriers).

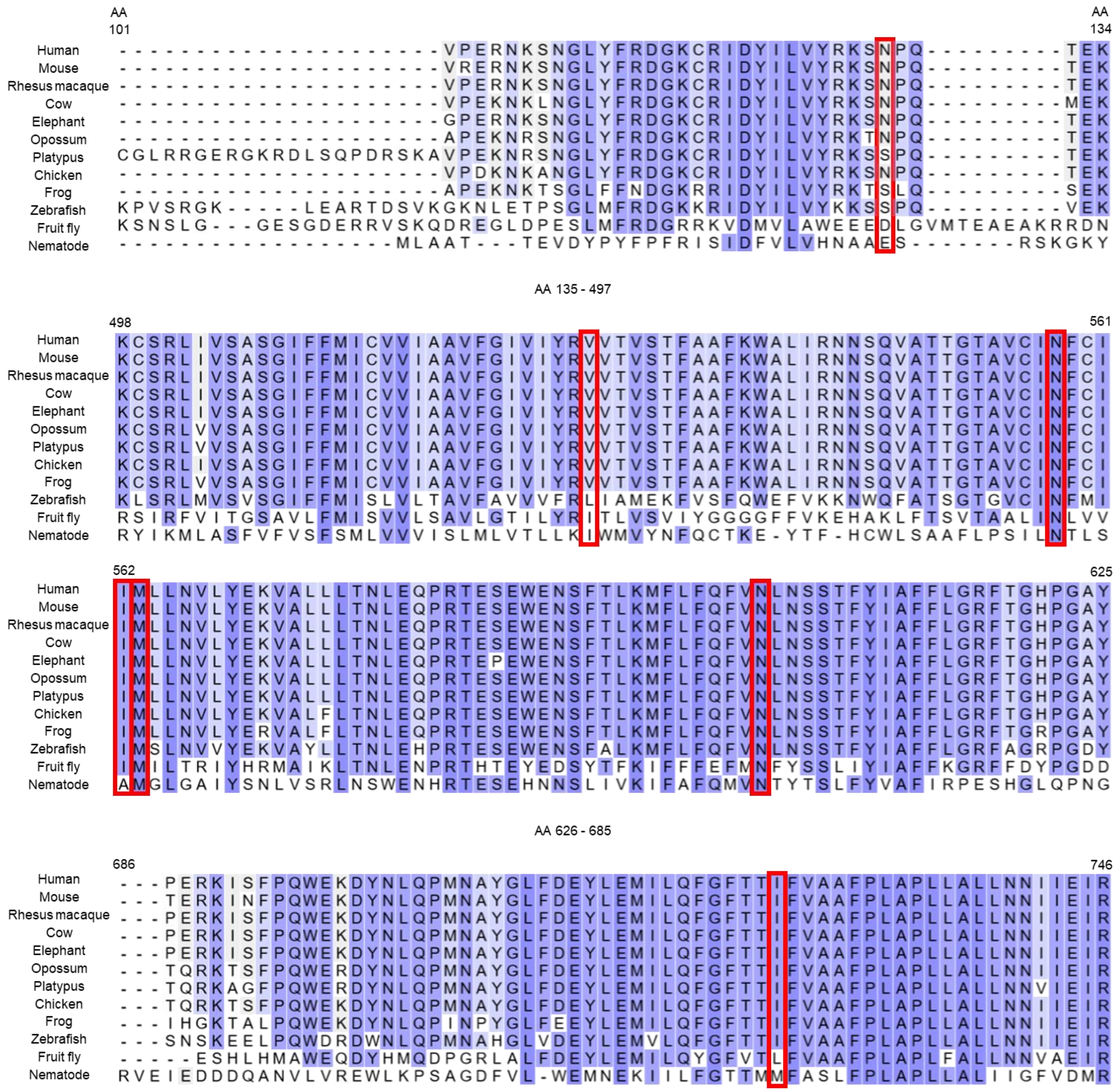


**Figure S3. Multiple sequence alignment of human *ANO4* with orthologues.** Multiple sequence alignment performed with Clustal Omega[3] between human *ANO4* and orthologues indicates sequence homology with mouse *Ano4* and evolutionary conservation at sites of the seven disease-associated variants investigated in this study highlighted in red.

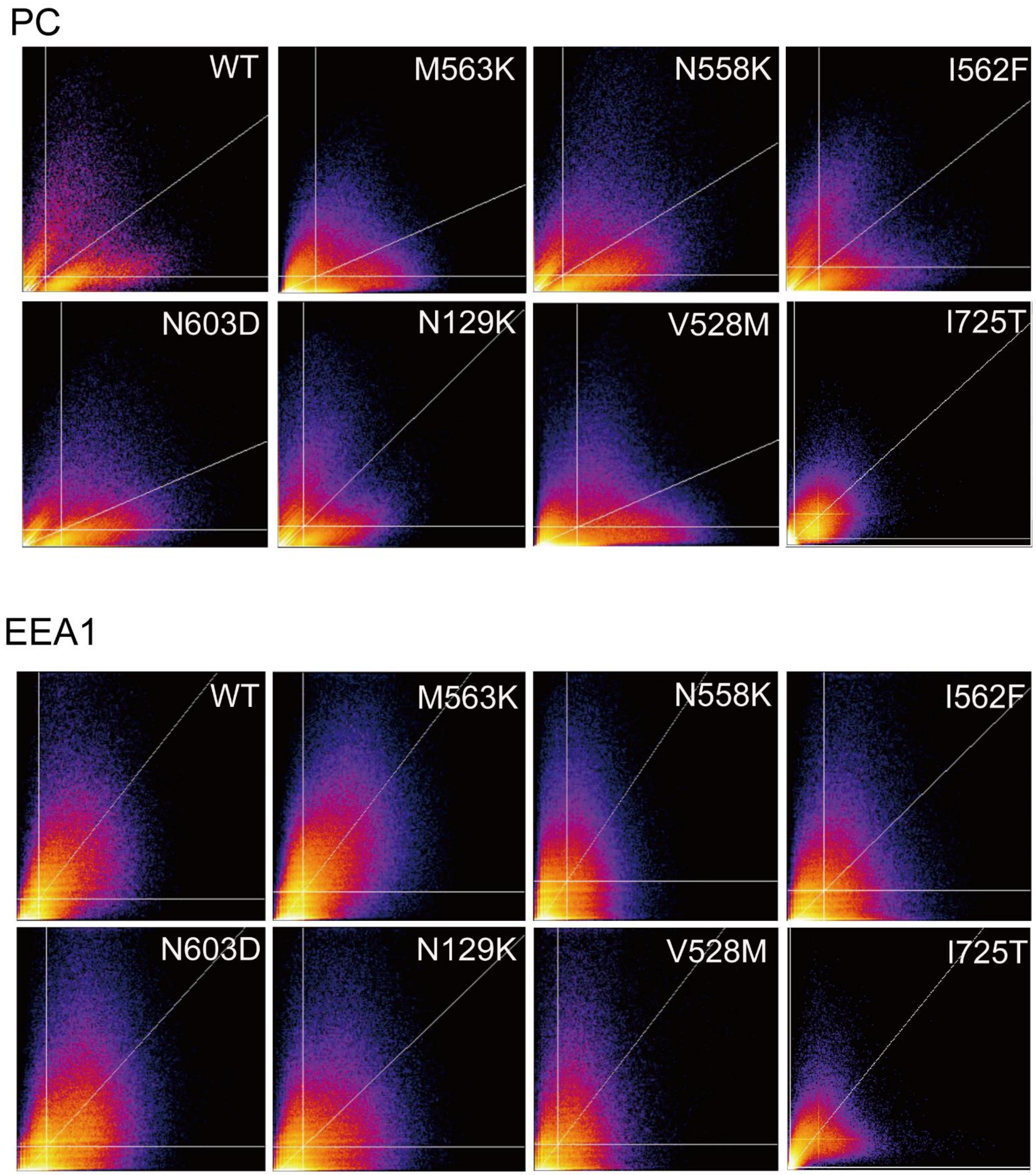


**Figure S4. Examples of pixel color spectra used for the calculation of Pearson's co-localization coefficient.** Pixel spectra for the co-localization analysis of ANO4 wildtype or mutants Met563Lys (M563K), Asn558Lys (N558K), Ile562Phe (I562F), Asn603Asp (N603D), Asn129Lys (N129K), Val528Met (V528M), and Ile725Thr (I725T) with pan-cadherin (PC) (upper panel) or the early endosome marker EEA1 (lower panel).

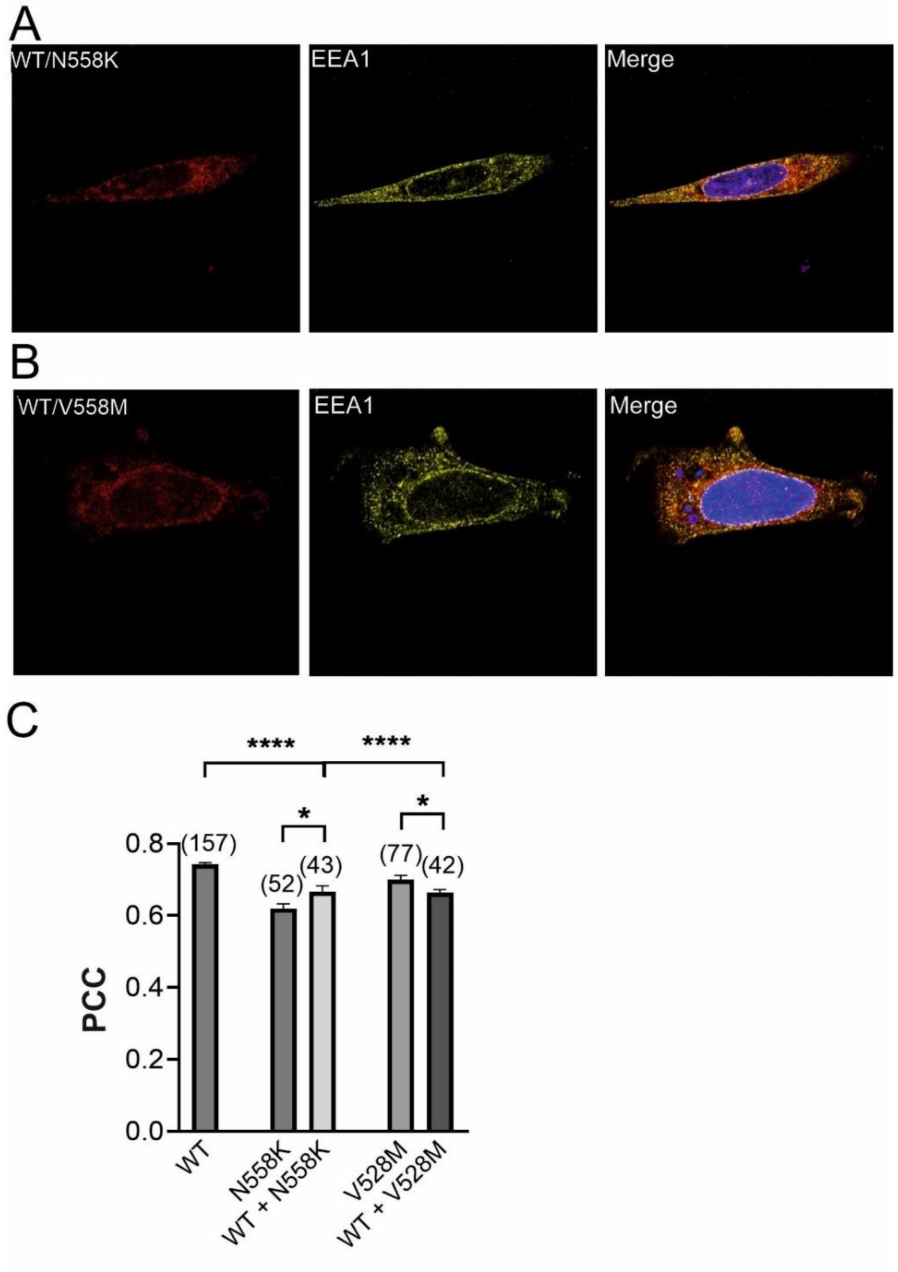


**Figure S5. Co-localization of ANO4 wildtype/Asn558Lys or wildtype/Val528Met with the early endosome marker EEA1 in HEK293 cells.** (A, B) HEK293 cells co-expressing wildtype and either Asn558Lys (N558K; A) or Val528Met (V528M; B) mutated *ANO4* constructs were stained with antibodies for ANO4 (left panel, red) and EEA1 (middle panel, yellow). Merged images (right panel) show the co-localization of ANO4 and EEA1. Nuclei were counterstained with DAPI (blue). (C) Pearson's correlation coefficient (PCC) analysis of EEA1 and mutant ANO4 (transfection and immunostaining according to (A-H). The number inside each bar represents n per group. Values are given as mean ± SEM. Multiple comparisons were performed by ANOVA with Tuckey's post hoc test. $^{*}p < 0.05$; $^{**}p < 0.01$; $^{***}p < 0.001$.

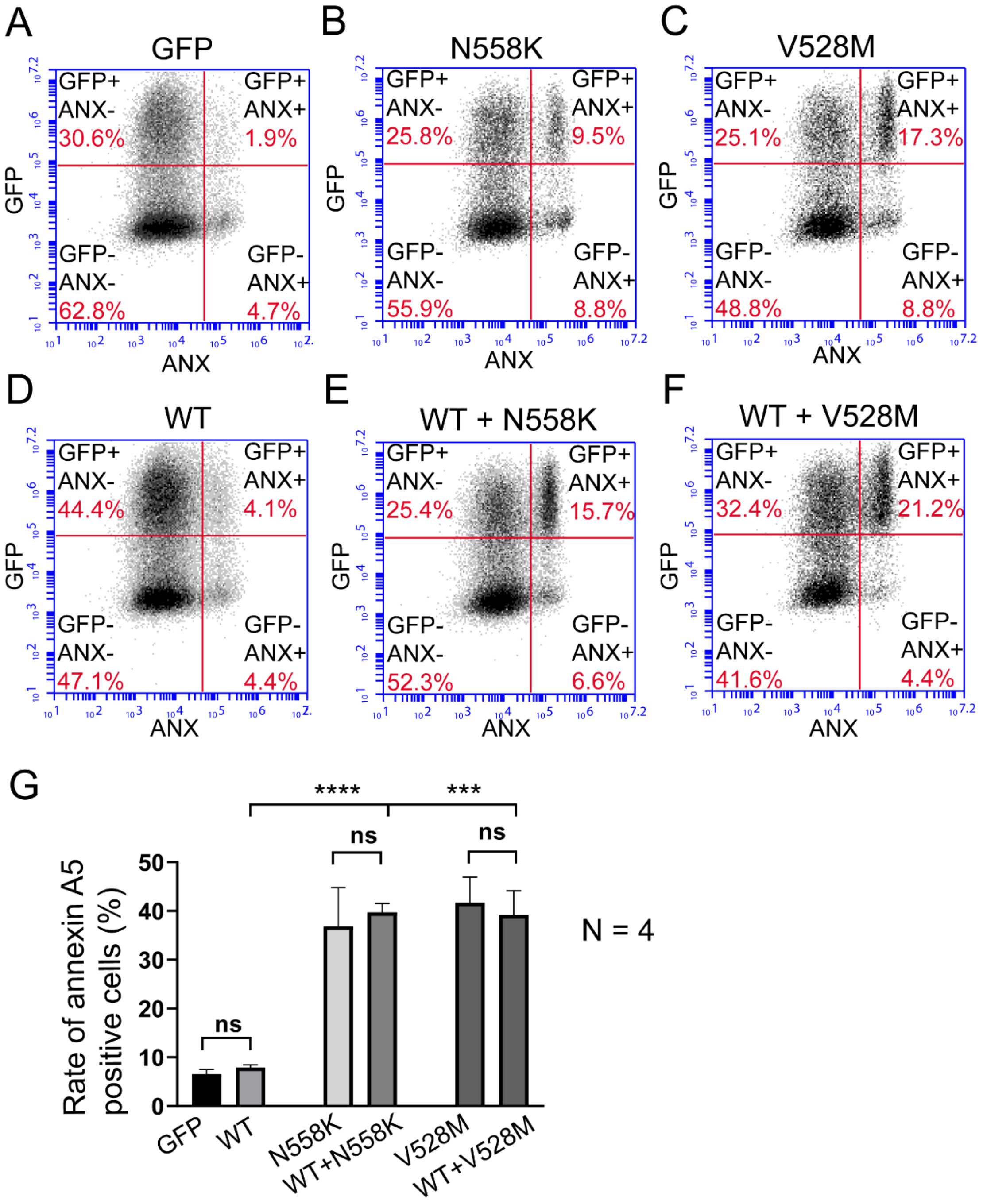


**Figure S6. Scramblase activity in HEK293 cells co-expressing wildtype and mutant ANO4 at physiological $Ca^{2+}$ levels.** Scramblase activity was assessed by FACS sorting of Annexin A5-labeled HEK293 cells that were not treated by ionomycin and transfected with GFP alone (A), ANO4 mutant Asn558Lys (N558K) plus GFP (B), ANO4 mutant Val528Met (V528M) plus GFP (C), wildtype ANO4 plus GFP (D), ANO4 wildtype and Asn558Lys (N558K) mutant plus GFP (E), or ANO4 wildtype and Val528Met (V528M) mutant plus GFP (F). X-axis: Fluorescence intensity of Anx A5-6S-IDCC (log); Y-axis: Fluorescence intensity of GFP (log). The right upper square represents the ANO4 transfected, Annexin A5-positive cell fraction. (G) Comparison of annexin A5 surface

expression between different transfection conditions. The experiments were carried out four times (N = 4). Values are given as mean ± SEM. Multiple comparisons were performed by ANOVA with Tuckey's post hoc test. *p < 0.05; **p < 0.01; ***p < 0.001.

## Supplemental References


1. Kang, H.J., Kawasawa, Y.I., Cheng, F., Zhu, Y., Xu, X., Li, M., Sousa, A.M.M., Pletikos, M., Meyer, K.A., Sedmak, G., et al. (2011). Spatio-temporal transcriptome of the human brain. Nature *478*, 483-489. 10.1038/nature10523.
2. Depondt, C., Van Paesschen, W., Matthijs, G., Legius, E., Martens, K., Demaerel, P., and Wilms, G. (2002). Familial temporal lobe epilepsy with febrile seizures. Neurology *58*, 1429-1433. 10.1212/wnl.58.9.1429.
3. Sievers, F., Wilm, A., Dineen, D., Gibson, T.J., Karplus, K., Li, W., Lopez, R., McWilliam, H., Remmert, M., Soding, J., et al. (2011). Fast, scalable generation of high-quality protein multiple sequence alignments using Clustal Omega. Mol Syst Biol *7*, 539. 10.1038/msb.2011.75.
